# Clarifying the Taxonomy of the Finch Louse Fly *Ornithomya Fringillina* (Curtis) (Diptera: Hippoboscidae) – An Analysis of Morphotypes

**DOI:** 10.1007/s11686-025-01113-z

**Published:** 2025-08-08

**Authors:** Denise C. Wawman, Abigail S. Bailey, Steven R. Fiddaman, Ben P. Jones, Nicholas Johnson, Adrian L. Smith

**Affiliations:** 1 Department of Biology, Edward Grey Institute of Field Ornithology, University of Oxford, South Parks Road, Oxford, OX1 3RB UK; 2 The John Krebs Field Station, Department of Biology, University of Oxford, Wytham, Oxford, OX2 8QJ UK; 3 Peter Medawar Building, Department of Biology, University of Oxford, Mansfield Road, Oxford, OX1 3SY UK; 4 Vector-Borne Disease Workgroup, Virology Department, Animal and Plant Health Agency, Woodham Lane, Addlestone, KT15 3NB UK; 5 Faculty of Health and Medical Sciences, University of Surrey, Guildford, GU2 7XH UK

**Keywords:** Taxonomy, Phylogeny, Morphometric analysis, Louse fly, Ectoparasite

## Abstract

**Background:**

The louse flies in the genus *Ornithomya* Latreille are avian ectoparasites. The patterns of alar microtrichia on the wings of the Ornithomyae are commonly used to help distinguish the various species, with the patterns in most species found to be constant between individuals. The Finch Louse Fly *Ornithomya fringillina* (Curtis) in the United Kingdom, Ireland and the Isle of Man, is unusual in that the several patterns have been described. Consequently it has a complicated taxonomic history and there is some confusion about species identification.

**Methods:**

Louse flies were collected by licensed bird ringers and an analysis of the simple morphological features, phenology and geographical distribution of these traits was performed.

**Results:**

No significant differences were found between the three main types, and it was concluded that the differences were not due to sexual dimorphism and did not provide evidence that the different forms were separate species. Analysis of COX1 DNA sequences confirmed this result and proved that these are indeed morphotypes and not distinct species. There was no geographical separation between COX1 sequences from the United Kingdom and those from flies sampled in other parts of the world. The molecular analysis also suggested that *Ornithomya bequaerti* (Maa) and *Ornithomya candida* (Maa) may not be valid species, but represent morphotypes of *O. fringillina*.

**Conclusions:**

The three patterns of alar microtrichia are morphotypes of a single species, Ornithomya fringillina. Further research is necessary to determine the status of some other species in the genus *Ornithomya*.

**Supplementary Information:**

The online version contains supplementary material available at 10.1007/s11686-025-01113-z.

## Introduction

The genus *Ornithomya* belongs to the family Hippoboscidae, which are ectoparasites of birds and mammals. Worldwide around 30 species are recognised in the genus [1, 2], of which four species are found within the United Kingdom (UK), Ireland and the Isle of Man (hereafter called “the region”) [3, 4]. While the recent UK colonist *Ornithomya biloba* (Dufour) is generally considered to be monoxenous on Barn Swallow *Hirundo rustica*, and is sometimes found on other Hirundines, and occasionally on the raptors which predate them [3–6], the other three species are more generalist, being found on a range of bird hosts [3, 7]. The Bird Louse Fly or Common Louse Fly *Ornithomya avicularia* (Linnaeus) is the largest of the group and is sympatric in the region with the smallest, *Ornithomya fringillina* (Curtis), both have a more southerly and lower altitudinal distribution than the Grouse Louse Fly *Ornithomya chloropus* (Bergroth) [7, 8].

*Ornithomya fringillina* is found in across the Palearctic Region, and into Africa North of the Sahara [9]. It is also found in East Asia; Korea [10] and Japan [11]. It may also occur in North America [12] and Iceland [13] but in both of these cases it was not distinguished from other species such as *O. chloropus*.

The taxonomy of *Ornithomya fringillina* and the other similar species of the genus *Ornithomya* was a source of disagreement between entomologists for many years in the twentieth century and remains a source of confusion today. The key historical events for these species in the Northern Hemisphere, excluding Asia, are summarised in Table 1 and briefly summarised here.

**Table 1:**
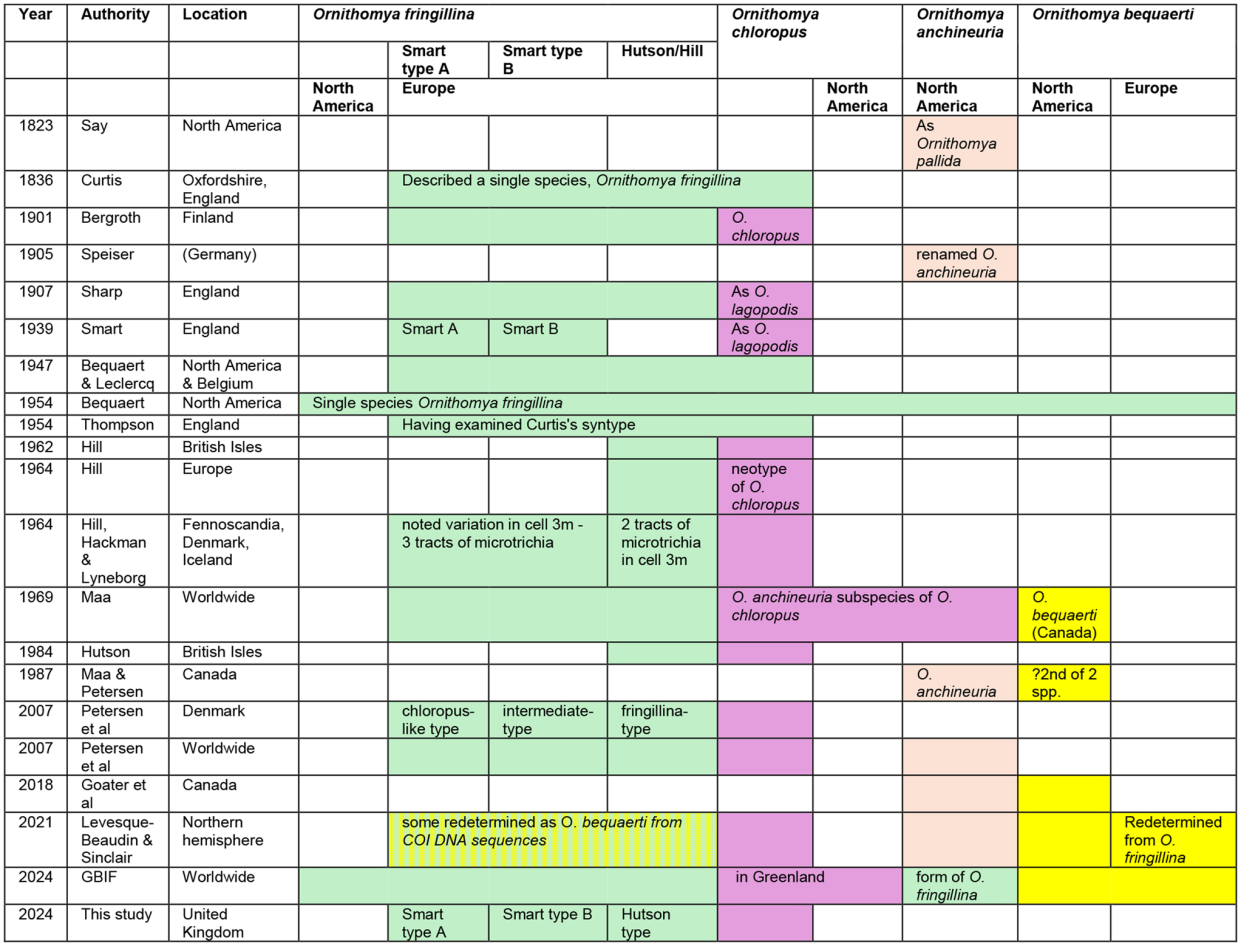
Timeline showing the major events in the classification of the smaller species of louse flies of the genus *Ornithomya* in Europe and North america. Each species is indicated by a colour: *O. fringillina*, green; *O. chloropus*, mauve; *O. anchineuria*, light brown; *O. bequaerti*, yellow. The morphotypes of *O. fringillina* are given separate columns and Europe and for each species there are separate columns for Europe and North America

In Europe, *O. fringillina* and *O. chloropus* were initially considered to be the same species, *O. fringillina* [14]. *Ornithomya chloropus* was first described in Finland by Bergroth in 1901 [15]. In 1907, Sharp, working in England, separated the smaller *Ornithomya* spp. in the region into *Ornithomya fringillina* and *O. lagopodis* (Sharp) [16] and Smart also recognised these two species [17]. *O. lagopodis* was later shown to be conspecific with *O. chloropus* [5]. In the 1940’s and 1950’s Bequaert, working alone in America [12] and with Leclercq in Belgium [18], and Thompson working in Great Britain and Ireland [19] recognised only one small species in the genus *Ornithomya* across the northern hemisphere until Hill revised the genus in the British Isles [16], reviewed specimens from Northern Europe [5] and described a neotype of *O. chloropus* – the original syntypes having been lost [15]. In North America *Ornithomya anchineuria* (Speiser) which was initially described from America as *Ornithomya pallida* (Say) in 1823 was renamed by Speiser due to another species having the same name [20]. Maa concluded that *Ornithomya anchineuria* should be considered a subspecies of *Ornithomya chloropus* as they were so closely related but also described *O. bequaerti* from Canada [21]. Later, Maa seems to have accepted *O. anchineuria* as a valid taxon and considered it to be one of two species present in North America, the other being *O. bequaerti* [22]. *Ornithomya anchineuria* was confirmed as a separate species by mitochondrial cytochrome oxidase I (COXI) DNA sequencing of a specimen from Canada [23]. However, the same study found that other specimens from both Europe and North America, morphologically identified as *O. fringillina*, fell into a group with *O. bequaerti*, considered by the authors to be distinct from *O. fringillina* [23] despite a relatively small phylogenetic distance calculated from a single short DNA sequence from a fly identified as *O. bequaerti*.

The Global Biodiversity information Facility (GBIF) website currently lists records of *O. fringillina* in North America and Australia, but considers *O. anchineuria* (Speiser), a North American species, and *O. variegata* (Bigot), an Australasian species, as synonyms of *O. fringillina* [24]. “*Ornithomya pallida* Say 1823*”*, is also listed as a synonym of *O. fringillina* on the GBIF website, whereas “*Ornithomya pallida* Latreille 1812”, is a synonym for the Swift Louse Fly *Crataerina pallida* (Latreille). *Ornithomya candida* (Maa), a species described from Japan [25] is also considered a valid species in GBIF [26].

**Fig. 1:**
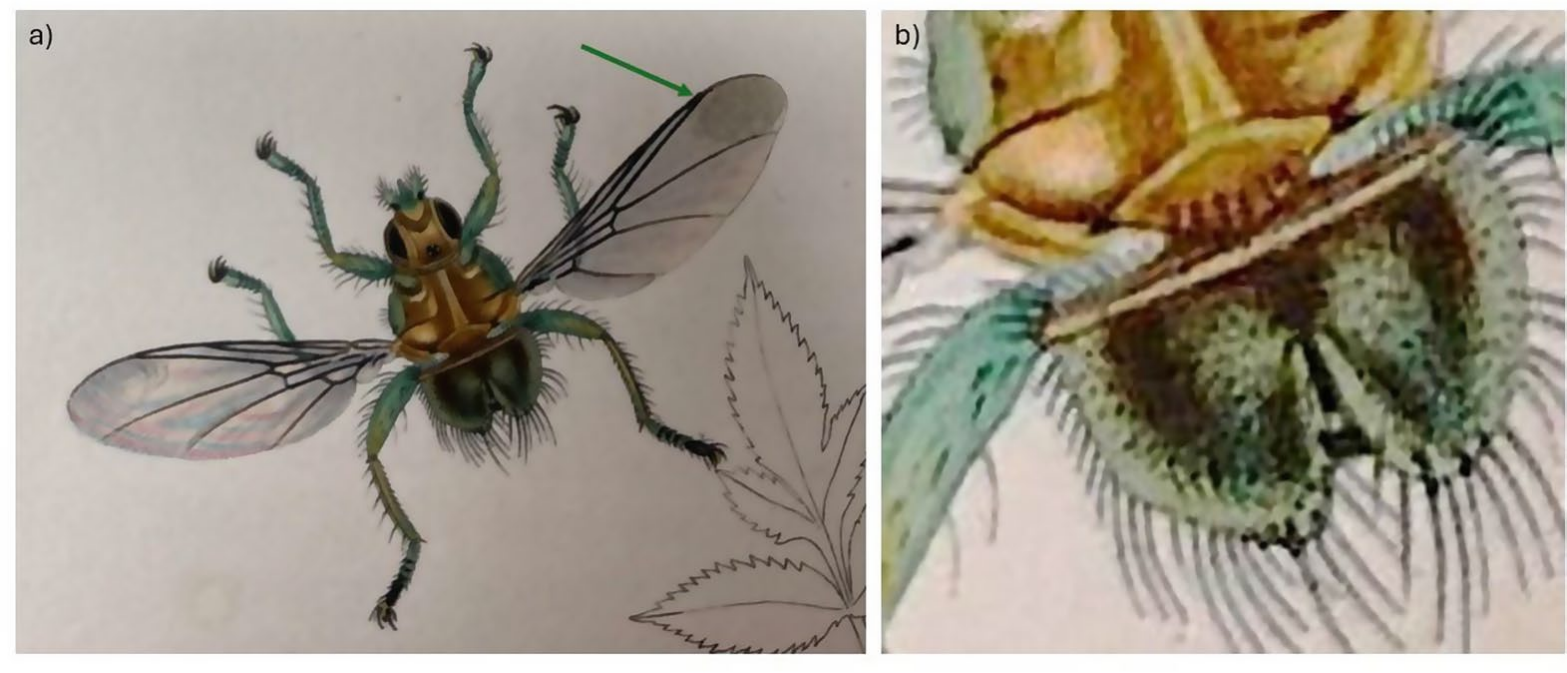
Images of the original illustration from John Curtis’s description of *Ornithomya fringillina* [14]: **a**, dorsal habitus, the right wing of which lacks a clear area adjacent to where the most distal vein reaches the costa (arrow) and the left wing appears to have been drawn to indicate iridescence; **b**) Close up of the scutellum, showing six strong bristles

**Fig. 2:**
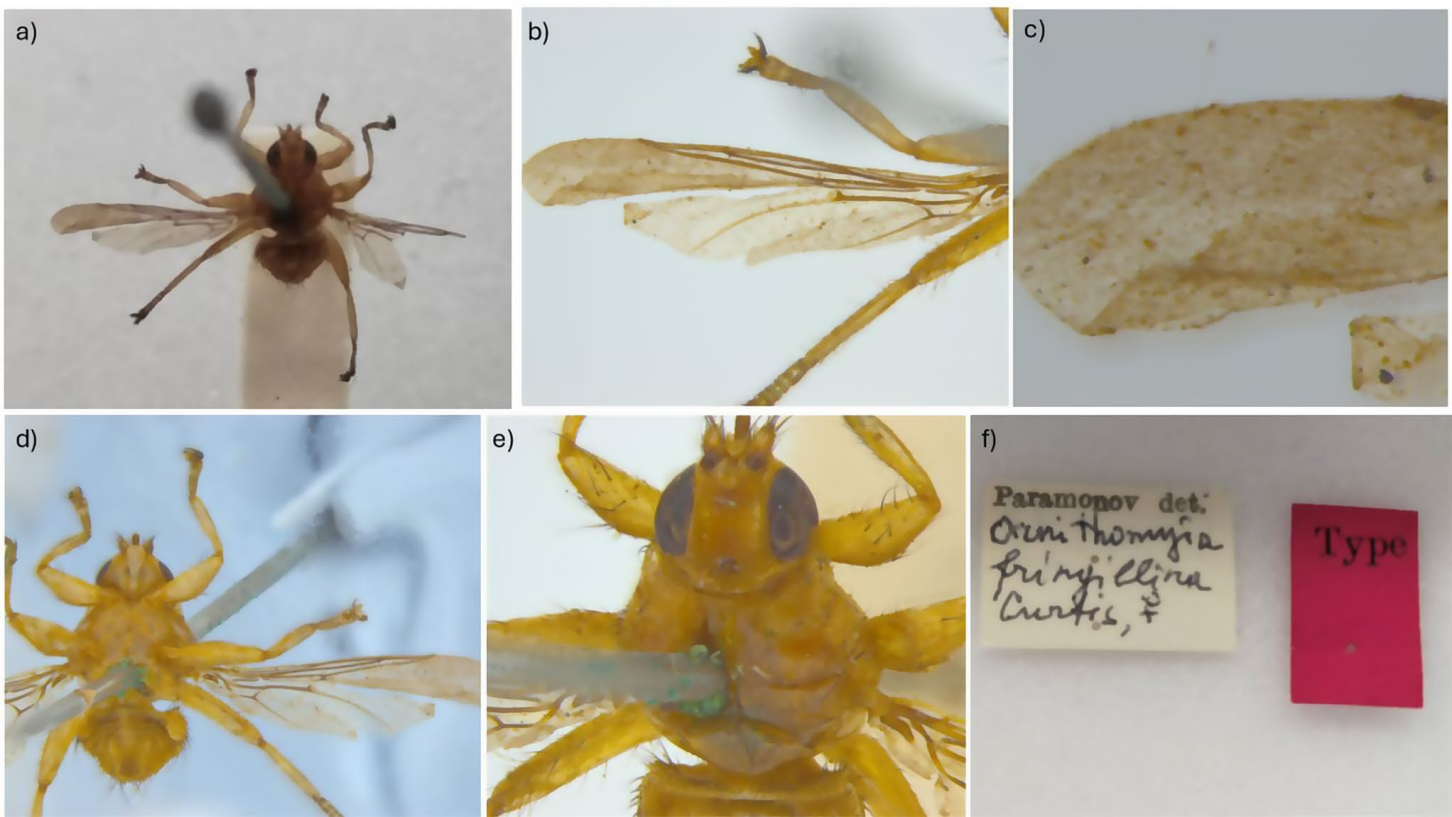
The syntype of *Ornithomya fringillina* held at Museums Victoria, Melbourne, Australia as photographed by Simon Hinkley, Museums Victoria, on 16th January 2023. **a**, dorsal habitus; **b**, left wing; **c**, distal part of left wing, with no clear area at the distal end of the vein adjacent to the costa; **d**, ventral abdomen; **e**, head and thorax; **f**, specimen labels

**Fig. 3:**
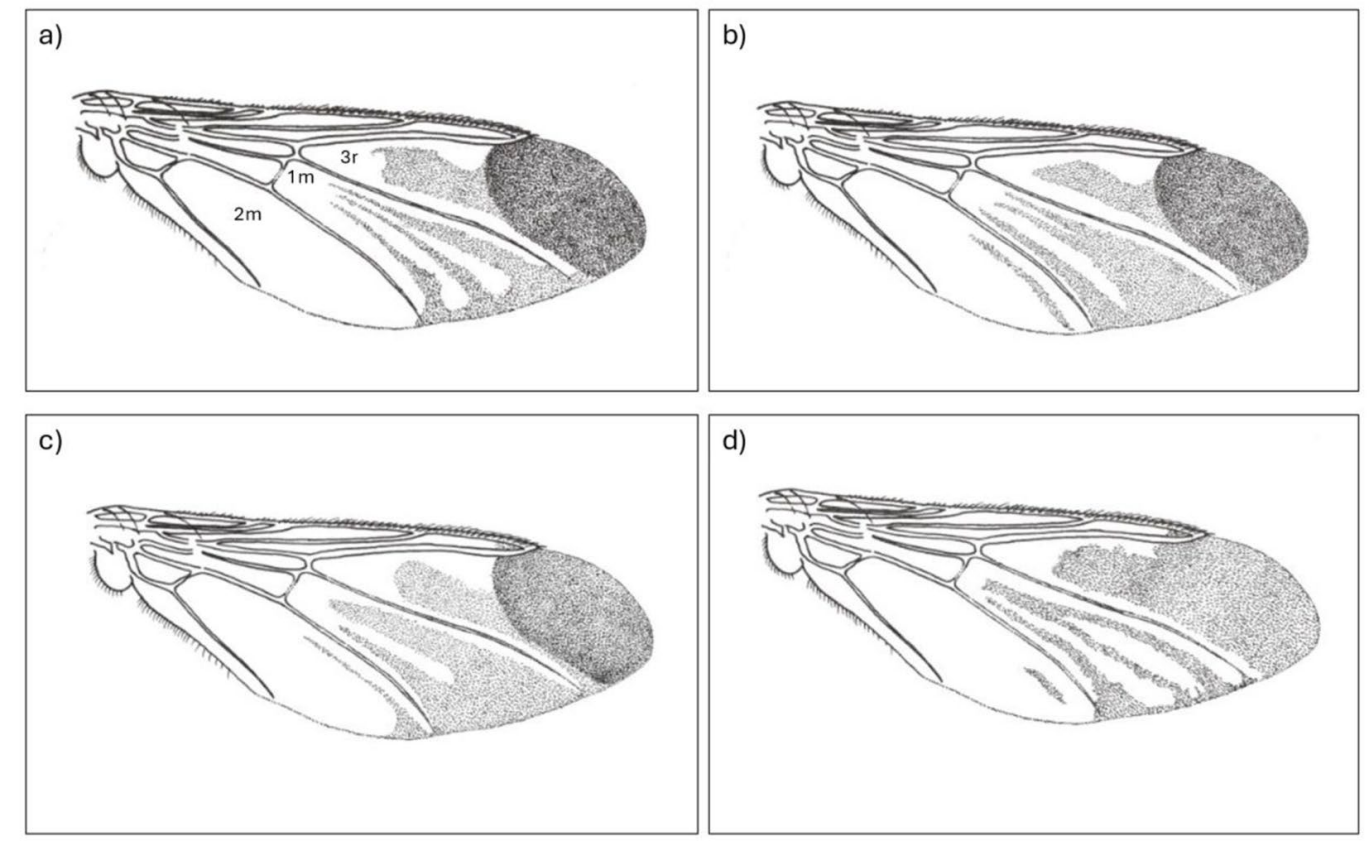
**a**-**c**. Wings of *Ornithomya fringillina*. **a**, **b**, redrawn from Smart (1939) showing three tracts of microtrichia in cell 1 m, referred to as Smart A and Smart B in the text; **c**, redrawn from a combination of from Hutson (1984) and Hill (1962b) showing only two tracts of microtrichia in cell 1 m; **d**, *Ornithomya anchineuria* redrawn from Maa (1969)

Part of the confusion would appear to lie with the brevity of the original written description of *O. fringillina*, its illustration (Fig. 1) [14] and the chosen type specimens, from Robin *Erithacus rubecula*, Great Tit *Parus major* and Yellowhammer *Emberiza citrinella*, collected at Weston-on-the-Green, near Oxford in the United Kingdom. Assuming there were three syntypes, one from each host species, two would appear to be lost and the third is now at the Melbourne Museum in Australia. Photographs of this extant syntype show a female fly (Fig. 2), with a reported wing length of just under 5 mm (Simon Hinkley, Museums Victoria, pers. comms.), that has sadly suffered some damage over the years making further redetermination difficult. It has acquired an undated additional determination label by the Russian entomologist Paramonov of “*Ornithomyia fringillina*” (sic.), presumably between 1947 and 1967 when he was working in Australia [27]. *Ornithomyia* and *Ornithomya* have both been used as the name for the genus [28]. This syntype was examined by Thompson, in the UK, who had the specimen posted from Australia, and his illustration [19] is of a wing with three lines of microtrichia in wing cell 1 m (See Fig. 3a for the cell numbers used), and a pattern of microtrichia in cell 3r part way between those seen in the two species *O. fringillina* and *O. chloropus*, which he thought “agreed in every respect with the common, smaller species of *Ornithomyia* (sic.), described by Bergroth as *O. chloropus* and Sharp as *O. lagopodis*” [12].

The illustration in the original description [14], shows a fly (Fig. 1) with six scutellar bristles – as are normally found in *O. chloropus* [3], and without an area clear of microtrichia in cell 3r towards the end of the veins at the leading edge of the wing, which is also a feature of *Ornithomya chloropus*. It should be noted that John Curtis was known for his skill as an illustrator and engraver and for the accuracy of his work [29]. Note was made in his obituary of his attention to detail, reporting his complaint to an illustrator that there were only 12 hairs on a fly’s tail not 13 [30]. Thus it is likely that the illustration is an accurate rendering of the actual fly and it may be that two species were present amongst the group of syntypes.

**Fig. 4:**
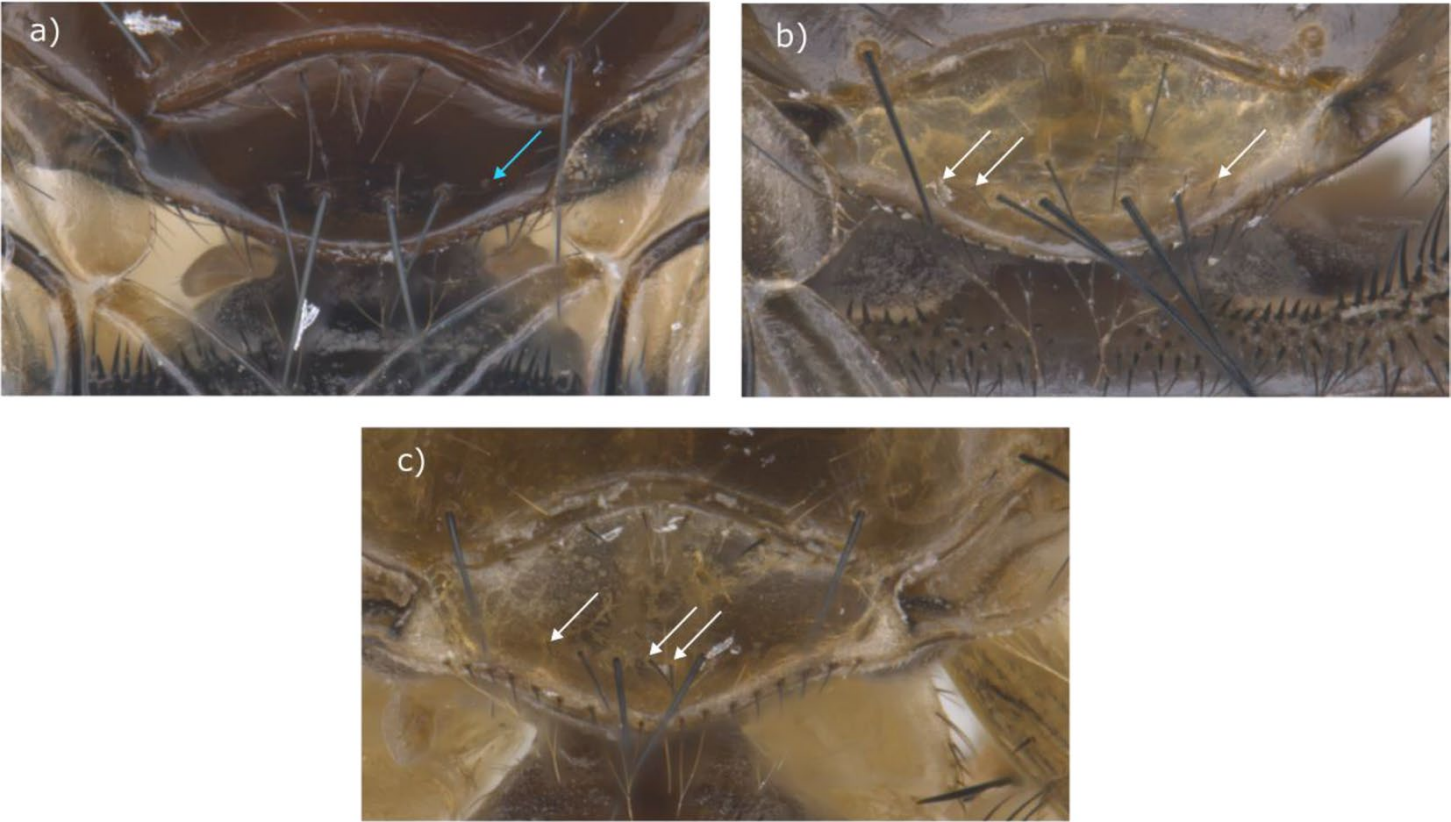
Scutellar bristles of *Ornithomya fringillina*. **a**, clearly has 4 bristles along the posterior edge of the scutellum, although it has the suggestion of a partially developed bristle pit (blue arrow). However, **b**, has three additional weak bristles, indicated by white arrows. Most entomologists would only count the stronger bristles, following Hutson [3], but some bristles can be ambiguous as seen in **c**, which has two strong bristles, three weak bristles (indicated by white arrows) and one bristle of medium strength

Taxonomic confusion between the two species has continued, despite the advent of DNA sequencing techniques. DNA sequencing has produced different results to the morphological identification of some individual specimens [23] probably because many morphological features exist across a range rather than as two distinct groupings [31]. The number of scutellar bristles may be difficult to define due to the missing bristles and the presence of weaker ones (Fig. 4) - and dark marks on the inferior surface of the head and thorax - have a tendency to fade over time, especially if specimens are preserved in ethanol [5].

The patterns of small hairs, variously called microtrichia or setulae, on the wings of the *Ornithomya* spp. have long been considered an important identification feature [3, 6, 9, 15–17, 21] and are considered to be one of the two most important features by some current researchers in North America (Valerie Levesque-Beaudin, pers. comm.), together with the shape of the tergites on the female which may not be well preserved in specimens [23]. Amongst the Ornithomyae in the region, the patterns of alar microtrichia are almost constant between individuals of each of three species, *O. avicularia*,* O. chloropus* and *O. biloba*, but highly variable in the fourth species, *O. fringillina.* In England, Smart [17] described two patterns of microtrichia in *O. fringillina*, both with three tracts of microtrichia towards the proximal end of cell 1 m, (Fig. 3a, b) but Hutson and Hill described a different form [3, 16] with only two tracts (Fig. 3c). One of the wing forms of *O. fringillina* described by Smart [17] is very similar, to that described for the North American species *O. anchineuria* [21] (Fig. 3d), with only slight variations in wing cell 3r which seems to be quite variable.

When checking morphologically separated *O. chloropus* and *O. fringillina* from Denmark, Petersen found a grouping of specimens, considered to be *O. fringillina*, which were genetically distinct from *O. chloropus* and had three wing types, described as “fringillina-type, chloropus-type and intermediate” [31], that would appear to correspond to the types Hutson, Smart A and Smart B, respectively. Doubt has been cast upon the identification of some of Petersen’s specimens (Levesque-Beaudin & Sinclair, 2021) used to produce a phylogeny [32], with Petersen’s *O. anchineuria* now grouped with *O. chloropus* and his *O. fringillina* now classed as *O. bequaerti* (Maa) in Levesque-Beaudin’s phylogeny.

The issues around the patterns of alar microtrichia have been further complicated by a key published in 2022 from work in Slovakia, which states that *Ornithomya fringillina* has four longitudinal stripes of microtrichia in the hind part of the wing, while *Ornithomya chloropus* has three [33], which contradicts earlier work and, when taken with other features in the key, would render a large number of Ornithomyae in the region unidentifiable.

This study aims to determine whether the variations amongst flies identified as *Ornithomya fringillina* on the basis of previously published accounts of the morphology of *Ornithomya fringillina* in the region [3, 17], are morphotypes of the same species or separate species, by examining a variety of morphological, ecological and biogeographic data, alongside DNA sequence analysis.

## Materials and Methods

The specimens of *Ornithomya fringillina* were obtained between 2020 and 2024 from UK licensed bird ringers collecting louse flies for the “Mapping the UK’s Flat Fly Project”. The collection methods have been previously published [4].

The louse flies were provisionally identified to species level using a key [3] and additional information as required [15–17, 21, 31]. The number of strong scutellar bristles were recorded. The wing length of each fly was measured to an accuracy of 0.1 mm with callipers, with the fly positioned on its back to straighten the wings. Notes were made of characteristics such as dark markings, on the mesothoracic basisternum, other areas of the ventral thorax, or on the lateral gena, medial to the eye, which have been used to separate *Ornithomya chloropus* from *Ornithomya fringillina* [3, 16, 31]. Although indistinct triangular markings on the lateral gena were not considered to be significant, and only those which were clearly defined were considered specific for *Ornithomya chloropus* by Petersen, these were recorded separately from clearer markings. Where possible the sex of each fly was recorded, by examination of the external genitalia or the darker patterns made by the tergites on the dorsal abdominal segments including the shape of tergite six.

Flies identified as *Ornithomya fringillina* were analysed on the basis of two classifications. All flies identified as *Ornithomya fringillina sensu lato (s.l.)* were divided into two groups, with either two or three tracts of microtrichia at the proximal end of cell 1 m. Additionally, from 1st August 2022 onwards, *Ornithomya fringillina s.l.* were divided into three groups, Smart type A, Smart type B and Hutson/Hill types based on those authors’ illustrations of the species [3, 16, 17]. The Hutson/Hill type will be referred to as the Hutson type as Hutson’s guide is freely available and widely used.

All grid references were checked using the website, Curaera [34], and Ordnance Survey grid references were converted to latitude and longitude using the website, Batch Convert Tool [35] for Great Britain and the Isle of Man, and Batch Coordinate Converter [36] for Ireland. The masses of host species were obtained from the British Trust for Ornithology’s “BTO Ringers Info App” [37].

The results were analysed in R version 4.1.2 [38]. Initial data checking and the addition of Julian Day and separate columns for day, month and year took place using the packages dplyr [39] and lubridate [40]. Principal component analyses (PCAs) were performed and the results plotted using the R packages factoextra [41], MASS [42], and ggplot2 [43].

Two separate sets of analyses were performed. The first compared flies in two groups, those with two and three tracts of microtrichia in cell 1 m, with the number of tracts being counted at the proximal end of the wing, thus combining morphotypes Smart A and Smart B. The second set of comparisons was made between all three described morphotypes (Smart A, Smart B and Hutson). The groups of flies were compared on the basis of simple morphological features (wing length and number of scutellar bristles), the sex of the fly, and biogeographical and ecological factors (latitude, longitude, altitude, phenology (Julian Day of collection), and host species body mass). Flies were excluded from these analyses if the wings were too damaged to be classified into morphotypes, the patterns of microtrichia were ambiguous (for example, if its two wings were different morphotypes) or if some of the data were missing.

For each PCA the data were sorted into suitable datasets and all non-numeric columns were removed, leaving just those listed above. Sex was coded as a categorical variable and the analyses were run twice: with sex included and excluded. Rows with missing values were excluded and the PCA was performed using a correlation matrix method with scaled data. PCA plots were checked for evidence of clustering of individual flies of the various morphotypes into groups.

Mitochondrial COXI sequences were obtained from a selected sample of flies collected by the project. Table 2 lists details of the accession numbers, species and morphotypes, host species, date and place of collection for all the specimens.

**Table 2:** Details of the specimens sequenced, including accession numbers, species, sex and morphotype, host species, date and location of collection, name of collector and laboratory where the flies were sequenced

Study specimen number	Accession number	Species & wing morphotype	Sex	Host species	Date	Site	Collector	Sequenced by
5366	Bold Systems UKAN1813-23	*Ornithomya avicularia*	♀	Starling *Sturnus vulgaris*	26.vi.2021	England, Norfolk, Thetford	Jo Lashwood	UKBoL
5371	Bold Systems UKAN1821-23	*Ornithomya avicularia*	♂	Starling *Sturnus vulgaris*	28.vi.2021	England, Norfolk, Thetford	Jo Lashwood	UKBoL
7979	Bold Systems UKAN1820-23	*Ornithomya chloropus*	♀	Meadow Pipit *Anthus pratensis*	8.ix.2021	Scotland, Highland, Carse of Ardersier	Hugh Insley	UKBoL
4152	Bold Systems UKAN1816-23	*Ornithomya chloropus*	♂	Siskin *Spinus spinus*	25.vi.2021	Scotland, Skye, Portree	Jonathan Jones	UKBoL
7755	Bold Systems UKAN1815-23	*Ornithomya fringillina* Hutson	♂	Goldcrest *Regulus regulus*	17.vii.2022	Scotland, Highland, Carse of Ardersier	Hugh Insley	UKBoL
5994	Bold Systems UKAN1819-23	*Ornithomya fringillina* Smart A	♀	Treecreeper *Certhia familiaris*	18.ix.2021	Scotland, Skye, Portree	Jonathan Jones	UKBoL
FF154	GenBank CAXBTB000000000.1	*Ornithomya fringillina* Hutson	♂	Great Tit *Passer domesticus*	16.vi.2022	England, Somerset, Minehead, Bratton	Denise Wawman	DToL
FF233/OF048	GenBank PQ068340	*Ornithomya fringillina* Hutson	♂	Blue Tit *Cyanistes caeruleus*	20.vii.2023	England, Somerset, Minehead, Bratton	Denise Wawman	APHA
FF260/OF080	GenBank PQ066342	*Ornithomya fringillina* Hutson	♀	Great Tit *Parus major*	21.viii.2023	England, Somerset, Withiel Florey	Denise Wawman	APHA
FF213/OF028	GenBank PQ066339	*Ornithomya fringillina* Smart B	♂	Robin *Erithacus rubecula*	15.vii.2023	England, Somerset, Minehead, Bratton	Denise Wawman	APHA
FF258/OF078	GenBank PQ066341	*Ornithomya fringillina* Smart B	♀	Goldfinch *Carduelis carduelis*	21.viii.2023	England, Somerset, Minehead, Bratton	Denise Wawman	APHA
FF194/OF009	GenBank PQ066338	*Ornithomya fringillina* Smart B	♂	House Sparrow *Passer domesticus*	7.vii.2023	England, Somerset, Minehead, Bratton	Denise Wawman	APHA
LIZZ/CP060	GenBank PQ066331	*Crataerina pallida*		Swift *Apus apus*	23.vii.2023	Wales, Powys, Hay on Wye	Lizzie Harper	APHA
FF199/OC014	GenBank PQ066335	*Ornithomya chloropus*	♂	House Sparrow *Passer domesticus*	7.vii.2023	England, Somerset, Minehead, Bratton	Denise Wawman	APHA
FF234/OA049	GenBank PQ066337	*Ornithomya avicularia*	♀	Great Tit *Parus major*	20.vii.2023	England, Somerset, Minehead, Bratton	Denise Wawman	APHA
FF223/OA038	GenBank PQ066336	*Ornithomya avicularia*	♀	Blackbird *Turdus merula*	18.vii.2023	England, Somerset, Minehead, Bratton	Denise Wawman	APHA
HJ247/OA057	GenBank PQ066333	*Ornithomya avicularia*	♂	Jackdaw *Coloeus monedula*	24.vii.2023	England, Lancashire, Mellor, Reaps	Hugh Jones	APHA
FF196/OA011	GenBank PQ066334	*Ornithomya avicularia*	♀	House Sparrow *Passer domesticus*	7.vii.2023	England, Somerset, Minehead, Bratton	Denise Wawman	APHA
JT01/OA101	GenBank PQ066332	*Ornithomya avicularia*	♀	Dunnock *Prunella modularis*	14.vii.2023	England, Merseyside, Wirral, Hoylake	Jane Turner	APHA
5291	GenBank PQ785575	*Crataerina* *pallida*	♂	Sparrowhawk *Accipiter nisus*	06.vii.2022	England, Yorkshire, Shirebrook Valley	Bryn Roberts	Oxford
1077	GenBank PQ785576	*Ornithomya* *avicularia*	♀	Chaffinch *Fringilla coelebs*	21.vii.2022	England, Lincolnshire, Owmby	Jenny Dunn	Oxford
1571	GenBank PQ785577	*Ornithomya* *fringillina* *Hutson*	♀	Whitethroat *Sylvia communis*	06.viii.2022	England, Northamptonshire, Stanford Reservoir,	Stanford Ringing Group	Oxford
5295	GenBank PQ785578	*Ornithomya* *biloba*	♀	Swallow *Hirundo rustica*	01.ix.2022	England, Derbyshire, Avenue Washlands Nature Reserve,	Bryn Roberts	Oxford
3235	GenBank PQ785579	*Ornithomya* *chloropus*	♀	Wren *Troglodytes troglodytes*	29.ix.2022	Scotland, Argyllshire, Glen Euchar	Rob Lightfoot	Oxford
H926	GenBank PQ785580	*Ornithomya* *avicularia*	♂	Robin *Erithacus rubecula*	12.vii.2022	England, East Sussex, Icklesham	Rye Bay Ringing Group	Oxford
1073	GenBank PQ785581	*Ornithomya* *fringillina* *Smart B*	♀	Whitethroat *Sylvia communis*	15.vii.2022	England, Lincolnshire, Owmby	Jenny Dunn	Oxford
H106	GenBank PQ785582	*Stenepteryx* *hirundinis*	♂	House Martin *Delichon urbica*	09.vii.2023	England, Northamptonshire, Lamport Hall	Northants Ringing Group	Oxford
A29	GenBank PQ799306	*Ornithomya* *chloropus*	♂	Red Grouse *Lagopus scotica*	04.viii.2022	Scotland, Angus, near Glen Esk	Anon.	Oxford
1003	GenBank PQ799307	*Ornithomya* *chloropus*	♂	Merlin *Falco columbarius*	25.vi.2022	England, County Durham	Y. Townsend	Oxford
SB37	GenBank PQ799308	*Ornithomya* *fringillina* *Smart A*	♀	Robin *Erithacus rubecula*	11.vii.2022	Ireland, Kerry, Killarney National Park	Sam Bayley	Oxford
7091D	GenBank PQ799309	*Ornithomya* *chloropus*	♀	Kestrel *Falco tinnunculus*	09.vii.2023	Wales, Montgomeryshire, Llanerfyl, Wern-Fach	Mid Wales Ringing Group	Oxford
X352	GenBank PQ799310	*Ornithomya* *fringillina* *Smart B*	♂	Robin *Erithacus rubecula*	10.viii.2023	England, Devon, Bridford	Samuel Gray	Oxford
1011	GenBank PQ799312	*Ornithomya* *chloropus*	♂	Merlin *Falco columbarius*	02.vi.2022	England, County Durham	Y. Townsend	Oxford

The genome of one specimen of *Ornithomya fringillina* was fully sequenced [44] by the Darwin Tree of Life Project (DToL) [45].

The COXI gene from six louse flies, two of each of *Ornithomya avicularia*, *Ornithomya chloropus* and *Ornithomya fringillina* – one with two tracts of microtrichia (Hutson type) and one with three (Smart A type) - were sequenced as part of the UK Barcode of Life Project (UKBoL) [46].

One specimen of *O. chloropus*, five specimens of *O. avicularia* and five specimens of *O. fringillina* and one *Crataerina pallida*, that had been preserved in RNAlater, were sequenced by BPJ using shotgun Illumina NGS sequencing at the Animal and Plant Health Agency laboratories (APHA). The fly was prepared by washing in phosphate buffered saline (PBS) to remove the RNAlater, then in 5% sodium hypochlorite solution to remove contaminants, and finally twice more in PBS to remove the sodium hypochlorite. DNA and RNA were isolated separately from the fly using the QIAgen AllPrep DNA/RNA MiniKit (Qiagen, Manchester, United Kingdom). Briefly, the fly was homogenized in 300 µl RLT buffer using a single 5 mm steel bead in a TissueLyserII (Qiagen) for 5 min at 30 Hz. After this, manufacturer’s instructions were followed and DNA was eluted into 60 µl buffer EB and RNA was eluted into 50 µl RNase-free water. Sequencing libraries were prepared using Nexetra XT kits (Illumina, Cambridge, UK) and sequencing using a Nextseq sequencer (Illumina, Cambridge, UK). Illumina) to generate 2 × 150 base paired-end reads. The raw data were filtered to remove adaptors and low quality reads using the programs fastp version 0.23.4 [47] and multiqc v 1.19 [48]. The sequences were aligned and non-Dipteran sequences removed in the program Bowtie2 [49]. Sequences were assembled using MEGAHIT [50]. Taxonomic classification was carried out using the program Kracken2 [51], with the program Bracken [52] to estimate species abundance and facilitate removal of poor quality and low abundance sequences. Sequences identified using these methods were isolated and the identity was confirmed using BLAST+ [53].

The DNA from the legs of a further 12 specimens, preserved in 70% ethanol, was extracted in Oxford by ASB and SRF. The legs were disrupted under liquid nitrogen and DNA was extracted using the Qiagen Blood and Tissue kit as per the manufactures instructions. The COXI gene fragments were amplified by PCR using primers LEPF1 ATT CAA CCA ATC ATA AAG ATA TTG G and LEPR1 TAA ACT TCT GGA TGT CCA AAA AAT CA (Integrated DNA Technologies, Belgium) at the manufacturer-recommended concentration of 0.5 μm, using the following cycling conditions, 5 min at 98 °C, 40 × (30 s at 98 °C; 30 s at 60 °C; 30 s at 72 °C) then 7 min at 72 °C.PCR products were visualised after separation on a 1% agarose gel, purified using the Qiagen PCR Clean up kit according to manufacturer’s instructions and then Sanger sequenced by Source BioScience, UK.

Additional sequences for the *Ornithomya* species, were obtained from NCBI GenBank and BoldSystems with the addition of the moth *Cabera pusaria* as an outgroup. The relationships between the species were initially explored using the R packages: seqinR [54], ape [55], adegenet [56], viridis [57], RSQLite [58] and DECIPHER [59], and a phylogeny produced from a 446 bp alignment using MrBayes [60] with 1,000,000 MCMC under HKY + G model (determined by ng-modeltest).

The final phylogeny was produced using a reduced number of the *Ornithomya avicularia*, retaining only the most distantly related sequence (OR064831 from Russia) and new sequences from the United Kingdom, to show the overall width of the clade. This final phylogeny was produced from sequences aligned and trimmed in R using the package DECIPHER and imported into MEGA version 12 [61]. The analysis in MEGA used three computing threads, 1000 replicates and the standard bootstrapping setting to produce a maximum likelihood tree, using a nearest neighbour interchange (NNI) and uniform rates in the Tamura-Nei model [62] of nucleotide substitutions.

## Results

A total of 848 *Ornithomya fringillina* were collected. Details of the specimens examined are given in Online Resource 1: Table S1. Summaries of the results for each group in the two analyses (using either two morphotypes or three morphotypes) are presented separately below. Photographs of the wings of the louse flies from the study, illustrating the different wing morphotypes, can be seen in the Online Resource 2: Figure S1.

Flies with two tracts of microtrichia at the proximal end of cell 1 m (Hutson type) were less frequently observed than those with three tracts of microtrichia (Smart A or Smart B types) being 15.4% of the total (116/753). There was no significant difference between the wing lengths, number of scutellar bristles, Julian Day and peak month of capture, or capture site characteristics of altitude, latitude and longitude, or host mass between either the two morphotypes (two and three tracts of wing microtrichia) or the three morphotypes (Smart A, Smart B and Hutson). To allow comparisons with the clearly defined species in the genus, the other species of *Ornithomyae* identified in the study are also included in the boxplots illustrating these results.

Teneral louse flies of all three morphotypes were identified from a single site (Table 3). Flies of different morphotypes were observed on the same host species, and found together on the same bird. The host species associations can be seen in Online Resource 3: Table S2.

**Table 3:** Teneral *Ornithomya fringillina* examined

Specimen number	Species	Sex	Site	Date	Host Species	Morphotype
**FF265**	*Ornithomya fringillina*	♀	Bratton, Minehead, Somerset, UK	25.viii.2023	Unknown	Smart B
**FF261**	*Ornithomya fringillina*	♀	Bratton, Minehead, Somerset, UK	25.viii.2023	Dunnock *Prunella modularis*	Smart A
**FF262**	*Ornithomya fringillina*	♀	Bratton, Minehead, Somerset, UK	25.viii.2023	Dunnock *Prunella modularis*	Smart B
**FF423**	*Ornithomya fringillina*	♂	Bratton, Minehead, Somerset, UK	10.vii.2024	House Sparrow *Passer domesticus*	Hutson
**FF424**	*Ornithomya fringillina*	♀	Bratton, Minehead, Somerset, UK	10.vii.2024	House Sparrow *Passer domesticus*	Smart B
**FF425**	*Ornithomya fringillina*	♂	Bratton, Minehead, Somerset, UK	10.vii.2024	Great Tit *Parus major*	Hutson
**FF428**	*Ornithomya fringillina*	♂	Bratton, Minehead, Somerset, UK	12.vii.2024	Unknown	Smart B

### Comparison of Two Versus Three Tracts of Microtrichia in Cell 1m

**Fig. 5:**
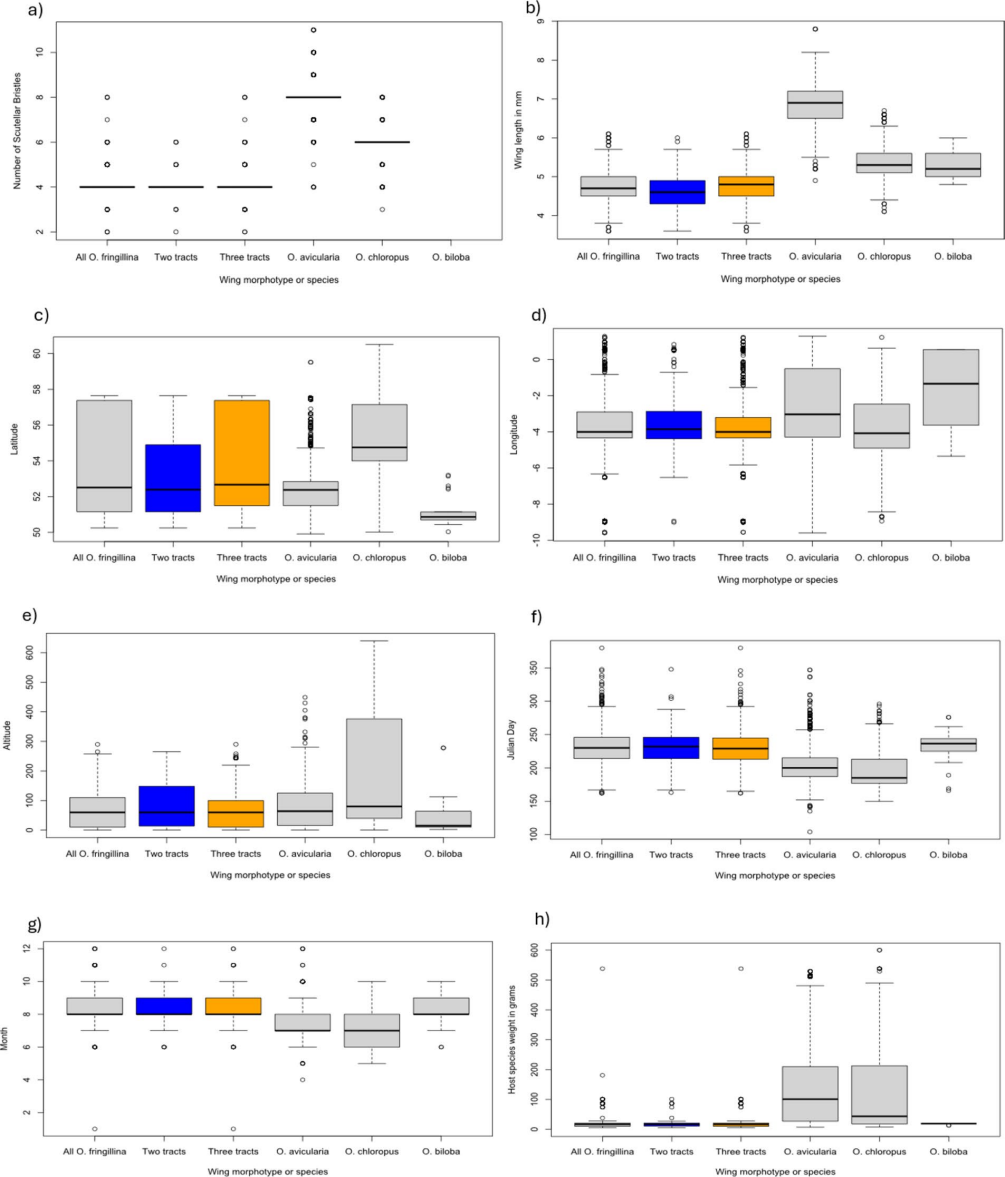
Boxplots showing the results of the analyses for two morphotypes of *Ornithomya fringillina*, that is, those with two and three tracts of microtrichia in wing cell 1 m. **a**, number of scutellar bristles; **b**, wing length; **c**, latitude of capture site; **d**, longitude of capture site; **e**, altitude of capture site; **f**, Julian Day on which the fly was taken; **g**, month; **h**, host mass

**Fig. 6:**
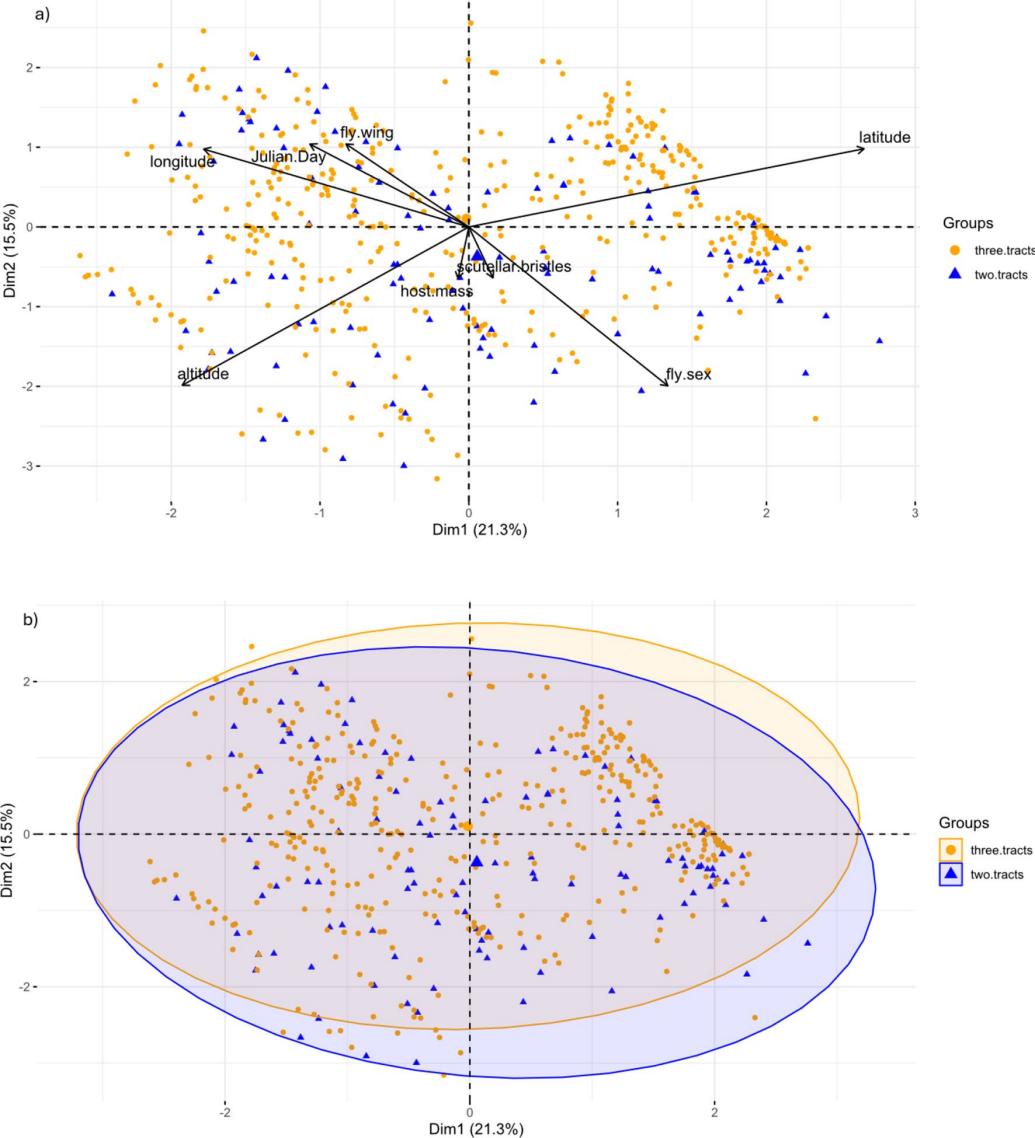
Results of the PCA comparing two morphotypes of *Ornithomya fringillina*, those with two and three tracts of microtrichia in wing cell 1 m. **a**, PCA biplot showing the relationship between the vectors representing the original variables and the first two principal components; **b**) individuals PCA, with group means represented by the larger symbols and concentration ellipses showing the similarity between the two groups

A total of 512 flies were included in this analysis, 266 were excluded from the PCA because of missing data, and four because one wing had three tracts of microtrichia and the other two. No significant differences were found between the two groups and there was no evidence that sexual dimorphism was responsible for the observed differences. A summary of these data can be seen in Table 4, the boxplots in Fig. 5, and the results of the PCA in Fig. 6, Online Resource 3: Table S2 and Online Resource 4:Fig. S2 and Table S3).

**Table 4:** Results of the morphometric, biogeographic and phenological analysis of two morphotypes of *Ornithomya fringillina* with either two or three tracts of microtrichia in wing cell 1m

	Two tracts of microtrichia	Three tracts of microtrichia	Total both groups
Number in sample	112	400	512
Fly wing length (mm)	4.6 (3.6–5.9)	4.8 (3.6–6.1)	4.7 (3.6–6.1)
Number of Scutellar Bristles	4 (3–6)	4 (2–8)	4 (2–8)
Latitude	53.57 (50.23–57.64)	53.98 (50.25–57.64)	53.89 (50.25–57.64)
Longitude	-3.82 (-9.01–0.64)	-3.58 (-9.56–1.23)	-3.63 (-9.56–1.23)
Altitude (metres)	81 (0–265)	72 (0–258)	74 (0–265)
Julian Day	232 (163–348)	230 (162–339)	230 (162–348)
Host mass (grams)	18 (5–101)	17 (5 -101)	17 (5–101)
Proportion of females	0.67	0.44	0.62

### Comparison of Three Patterns of Microtrichia as Described by Smart (1939) and Hutson (1984)

**Fig. 7:**
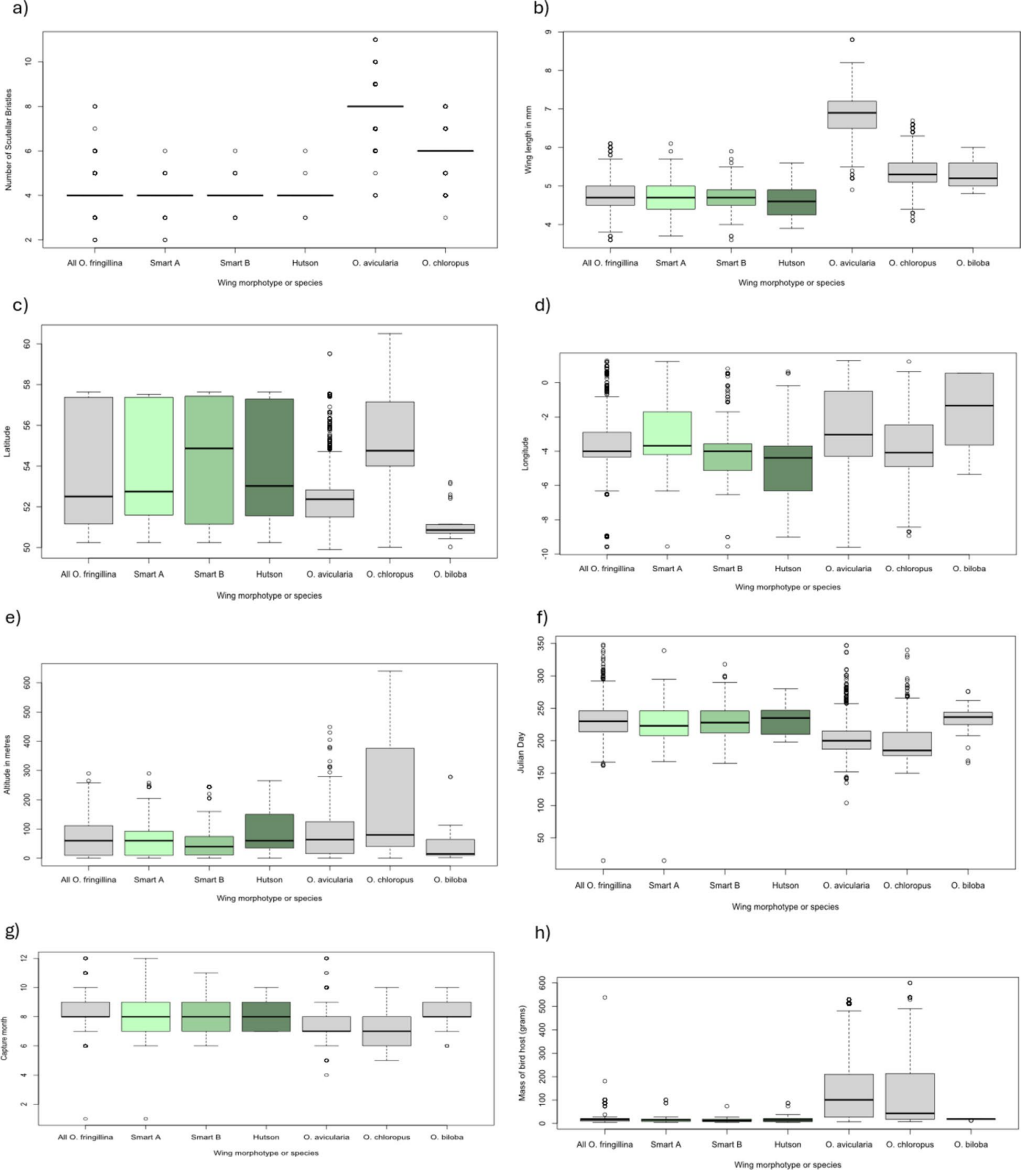
Boxplots showing the results of the analyses for three morphotypes of *Ornithomya fringillina*, that is, those with two and three tracts of microtrichia in wing cell 1m. **a**, number of scutellar bristles; **b**, wing length; **c**, latitude of capture site; **d**, longitude of capture site; **e**, altitude of capture site; **f**, Julian Day on which the fly was taken; **g**, month; **h**, host mass (capped at 600 g)

**Fig. 8:**
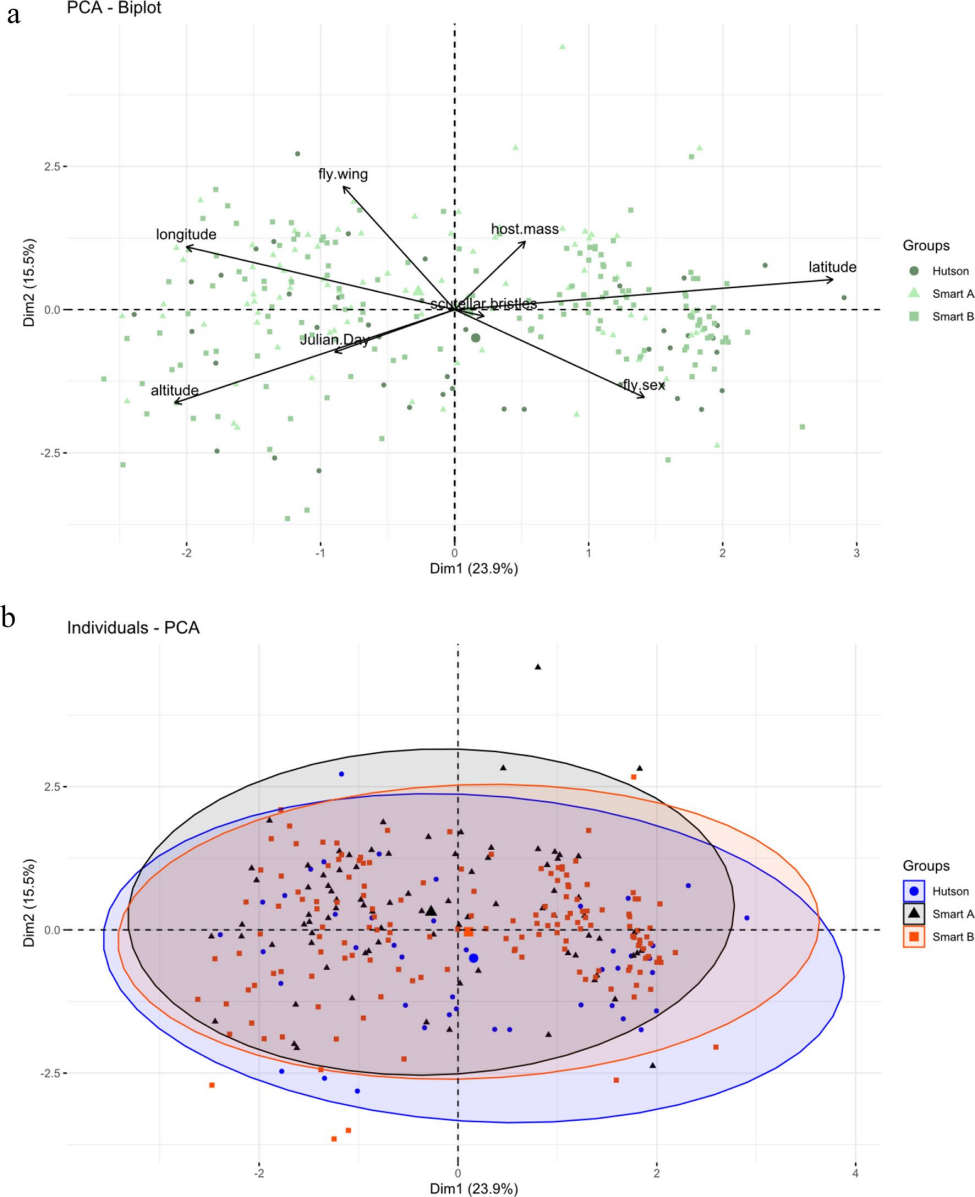
Results of the PCA comparing three morphotypes of *Ornithomya fringillina*, with different tracts of microtrichia in wing cell 1 m as described my Smart (1939) and Hutson (1984). **a**, PCA biplot showing the relationship between the vectors representing the original variables and the first two principal components; **b**) individuals PCA, with group means represented by the larger symbols and concentration ellipses showing the similarity between the three groups

A total of 299 flies were used in this analysis. 24 flies were excluded because of missing data and two because they had one wing of the Hutson morphotype and one of the Smart B morphotype. There was no significant difference between these morphotypes (Table 5. Figures 7 and 8, Online Resource 3:Table S2 and Online Resource 4: Table S4, Figure S3).

**Table 5:** Results of the morphometric, biogeographic and phenological analysis of the three morphotypes of *Ornithomya fringillina* based on the descriptions of the tracts of microtrichia in wing cell 1m by smart (1939) and Hutson (1984)

	Hutson	Smart A	Smart B	Total
Number in sample	44	96	159	299
Fly wing length (mm)	4.6 (3.9–5.6)	4.7 (3.7–6.1)	4.7 (3.6–5.9)	4.7 (3.6–6.1)
Number of Scutellar Bristles	4 (3–6)	4 (2–6)	4 (3–6)	4 (2–6)
Latitude	53.92 (50.25–57.64)	53.99 (50.25–57.52)	54.42 (50.25–57.64)	54.21 (50.25–57.64)
Longitude	-4.25 (-9.01–0.64)	-3.69 (-9.56–1.23)	-3.8 (-9.56–0.81)	-3.76 (-9.56–1.23)
Altitude (metres)	94 (0–265)	59 (0–258)	68 (0–244)	72 (0–265)
Julian Day	232 (198–280)	227 (168–339)	230 (165–318)	230 (165–339)
Host mass (grams)	15 (5–87)	15 (5–101)	13 (5–74)	16 (5–101)
Proportion of females	0.41 (*n* = 44)	0.79 (*n* = 88)	0.64 (*n* = 86)	0.65 ( *n* = 284)

### Analyses of DNA Sequences

**Fig. 9:**
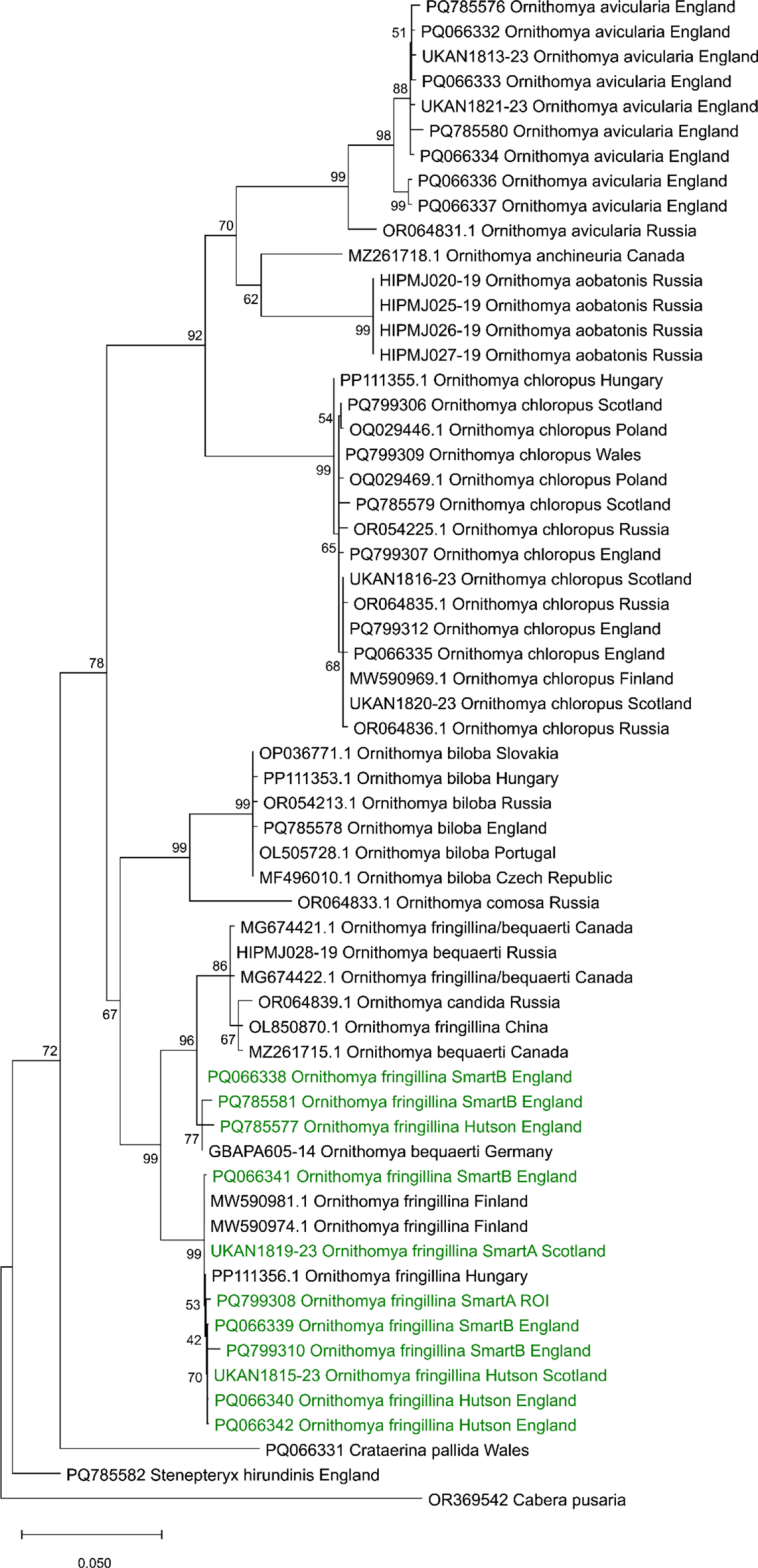
Phylogeny of the genus *Ornithomya*, produced from the COXI sequences, showing the morphotypes of *Ornithomya fringillina*, other species from the United Kingdom and Ireland (in green) compared to other sequences of the *Ornithomya* sp. from across their ranges. The nodes are labelled with the bootstrap values and the scale bar shows the percentage difference between sequences. The moth *Cabera pusaria* included as an outgroup. This phylogeny was produced from sequences aligned in R using the package DECIPHER and imported into MEGA version 12 (Kumar et al., 2024) for analysis, using three computing threads, 1000 replicates and the standard bootstrapping setting to produce a maximum likelihood tree, using a nearest neighbour interchange (NNI) and uniform rates in the Tamura-Nei model (Tamura and Nei, 1993) of nucleotide substitutions The tree with the highest log likelihood (-50,313.95) is shown

A phylogenetic analysis (Fig. 9) showed that the COX1 sequences obtained all the *Ornithomya fringillina* morphotypes lay within a single clade, with less than 2% variation between the sequences, and no clustering of the morphotypes, or by geographical location. Within this main clade, there are two subclades, one containing only *O. fringillina* (but including all three morphotypes), and the second containing specimens (*n* = 3) identified as *O. bequaerti*, two initially identified as *O. fringillina* but later redetermined as *O. bequaerti*, *O. candida* and two of the morphotypes (Hutson and Smart B) from the United Kingdom. It should be noted that only two specimens of the Smart A morphotype were successfully sequenced, so no conclusions can be drawn from their absence from the second clade. *Ornithomya anchineuria* falls within a different distinct clade.

## Discussion

Smart described two wing forms of *Ornithomya fringillina* [17] which were similar to those of the North American species, *Ornithomya anchineuria* described by Maa [21] whereas Hill and Hutson described one contrasting pattern [3, 16]. This study looked at the differences between these three morphotypes and between flies with either two or three tracts of microtrichia in wing cells 1 m and found no significant difference in their other morphological features, nor in their ecological or biogeographical traits. Louse flies of different morphotypes were found on the same host species, and on the same individual birds. The wing length, number of scutellar bristles, geographic and altitudinal distributions and phenology are almost identical. The differences were not due to sexual dimorphism and teneral flies of all three morphotypes were caught at a single site suggesting that the differences were not due to either wing wear or geographical variation. Six flies had one wing of one morphotype and the other of a different morphotype. The morphotype COX1 sequences grouped together in the phylogenetic tree further supporting that these are variants of a single species (based on 12 flies: two of the Smart A, five of Smart B and five of the Hutson morphotypes).

It is known that the wings of flies of some species wear as they age, for example, the related Tsetse flies *Glossina* spp [63]. The wing fray of flies such as *Lucilia sericata* has been used as a technique to calculate the post-mortem interval in forensic pathology [64]. Differential patterns of wear, could result in the loss of microtrichia from some areas of the wing, resulting the appearance of morphotypes, from the Hutson-type, via Smart type B, to Smart type A, and some older flies appear to have bald patches (Wawman, pers. obs). However, the absence of differences in the phenology between the various morphotypes, together with teneral flies being caught of all three morphotypes, is good evidence that the differences are not due to wear.

Taken together, the results the analyses of morphological features (the number of scutellar bristle and wing length), phenology (Julian Day of capture) and geographical distribution (latitude, longitude and altitude of capture site), all of which showed no significant differences in this study, would support the hypothesis that the flies with three patterns of microtrichia are morphotypes of the same species, *Ornithomya fringillina*, and not separate species. This was confirmed by DNA barcoding. This makes *Ornithomya fringillina* unusual amongst the other species in the genus Ornithomya which have more consistent patterns of wing microtrichia.

Although based only on very short sequences, the results of DNA sequencing suggested that the North American taxon *Ornithomya bequaerti* and the Japanese species *Ornithomya candida* may not be separate species. Further research is needed to confirm this observation, perhaps including more comprehensive sequence data (e.g. full mitochondria or whole genome sequencing), and dissection of male genitalia of multiple specimens of all of the relevant species, all of which is beyond the scope of this study.

## Supplementary Information

Below is the link to the electronic supplementary material.

Supplementary Material 1

Supplementary Material 2

Supplementary Material 3

Supplementary Material 4

## Data Availability

Details of all of the flies examined can be found in table S1. New DNA sequences are available in GenBank accession numbers PQ066331-42, PQ799306-12 and PQ785575-82. All sequences are listed in Table 2.

## References

[1] Dick CW Checklist of the world Hippoboscidae (Diptera: Hippoboscoidea). 2018;(January). Available from: https://www.researchgate.net/publication/322579074_CHECKLIST_OF_WORLD_NYCTERIBIIDAE_DIPTERA_HIPPOBOSCOIDEA

[2] Yatsuk AA, Nartshuk EP, Matyukhin AV (2024) Description of a new louse fly species of the genus Ornithomya Latreille, 1802 (Diptera: Hippoboscidae) from Irkutsk, Russia. Cauc Entomol Bull [Internet].;20(1):83–7. Available from: https://zenodo.org/records/10894260

[3] Hutson AM, Keds (1984) Flat-flies and Bat-Flies: Diptera, Hippoboscidae and Nycteribiidae. Handbooks Identif Br Insects [Internet].;10(7):40. Available from: https://www.royensoc.co.uk/sites/default/files/Vol10_Part07_Hutson.pdf

[4] Wawman DC (2024) Ornithomya biloba, Pseudolynchia garzettae and Pseudolynchia canariensis (Diptera: Hippoboscidae): three new United Kingdom colonists and potential disease vectors. Med Vet Entomol [Internet].;38(2):160–71. Available from: https://resjournals.onlinelibrary.wiley.com/doi/full/10.1111/mve.12703 10.1111/mve.12703 38059689

[5] Hill DS, Hackman W, Lyneborg L (1964) The genus Ornithomya (Diptera: Hippoboscidae) in fennoscandia, Denmark and Iceland. Not Entomol XLIV:33–52

[6] Hutson AM (1981) A new species of the Ornithomya biloba-group (Dipt., Hippoboscidae) from Crag Martin (Ptyonoprogne rupestris (Aves, Hirundinidae). Mitteilungen der Schweiserischen Entomol Gesellshaft; Bull la Soc Entomol Suisse [Internet].;54:157–62. Available from: https://www.e-periodica.ch/digbib/view?lang=en%26pid=seg-001%3A1981%3A54%3A%3A455#166

[7] Hill DS (1962) A study of the distribution and host preferences of three, species of Ornithomyia (Diptera: Hippoboscidae) in the British Isles.;37(4–6):37–48. Available from: http://doi.wiley.com/10.1111/j.1365-3032.1962.tb00286.x

[8] Wawman DC (2025) Citizen scientists mapping the United Kingdom’s and Republic of Ireland’s flat flies (louse flies) (Diptera: Hippoboscidae) reveal a vector’s range shift. Med Vet Entomol [Internet].;1–17. Available from: https://resjournals.onlinelibrary.wiley.com/doi/full/10.1111/mve.12795 10.1111/mve.12795 PMC12323749 39921336

[9] Maa TC (1964) On the genus Ornithomya Latreille from Africa (Diptera: Hippoboscidae). J Med Ent I(2):197–205

[10] Iwasa M, Choi CY (2013) Contribution to the Knowledge of the Hippoboscidae (Diptera) From the Republic of Korea. J Med Entomol [Internet].;50(2):231–6. Available from: https://academic.oup.com/jme/article-lookup/doi/10.1603/ME12179 10.1603/me12179 23540108

[11] Maa TC (1969) A revised checklist and concise host index of Hippoboscidae (Diptera). Pacific Insects Monogr [Internet].;20:261–99. Available from: http://hbs.bishopmuseum.org/fiji/pdf/maa1969b.pdf

[12] Bequaert JC (1954) The Hippoboscidae or louse-flies (Diptera) of mammals and birds part II. Taxonomy, evolution and revision of American genera and species. Entomol Am XXXIV:3–181

[13] Messersmith DH (1982) A report on a collection of Diptera from Iceland and Greenland. Fauna nor Ser B nor J Entomol 29(1):38

[14] Curtis J (1936) Ornithomyia fringillina. Br Entomol 13:582–583

[15] Hill DS (1964) A neotype for Ornithomya chloropus Bergroth, 1901 (Dipt. Hipp). Not Entomol XLIV:105–112

[16] Hill DS, Revision of the British, species of Ornithomyia Latreille (Diptera: Hippoboscidae) (1962). Proc R Entomol Soc London Ser B, Taxon [Internet].;31(1–2):11–8. Available from: 10.1111/j.1365-3113.1962.tb01163.xhttp://doi.wiley.com/

[17] Smart J (1939) Hippoboscidae. In: Edwards FF, Oldroyd H, editors. Blood-sucking flies [Internet]. England: Printed by order of the Trustees of the British Museum; pp. 118–24. Available from: https://babel.hathitrust.org/cgi/pt?id=umn.319510004583588%26view=1up%26seq=7%26skin=2021

[18] Bequaert J, Leclercq M (1947) Revision des Hippoboscidae de Belgique. Bull Ann La Société Entomol Belgique 83:77–84

[19] Thompson GB (1954) V.—Contributions toward a study of the ectoparasites of British birds and mammals.—No. 2. Ann Mag Nat Hist [Internet].;7(73):17–39. Available from: https://www.tandfonline.com/doi/full/10.1080/00222935508651820

[20] Speiser P (1905) Beiträge zur Kenntnis der Hippobosciden. (Dipt). Zeitschrift für Syst hymenopterologie und dipterologie [Internet].;5:347–60. Available from: https://www.biodiversitylibrary.org/part/41769

[21] Maa TC, Pacific Insects (1969) Notes on the Hippoboscidae (Diptera), II [Internet]. Vol. 20,. Available from: http://scholar.google.com/scholar?hl=en%26btnG=Search%26q=intitle:NOTES+ON+THE+HIPPOBOSCIDAE+(Diptera),+1#0

[22] Maa TC, Petersen BV (1987) Hippoboscidae. In: McApline JF (ed) Manual of Nearctic Diptera agriculture Canada monograph no 28. biosystematics research centre (formerly Institute). Research Branch, Agriculture Canada, Ottawa, ontario, pp 1271–1281

[23] Levesque-Beaudin V, Sinclair BJ (2021) Louse fly (Diptera, Hippoboscidae) associations with raptors in southern Canada, with new North American and European records. Int J Parasitol Parasites Wildl [Internet].;16(July):168–74. Available from: 10.1016/j.ijppaw.2021.09.007 10.1016/j.ijppaw.2021.09.007 PMC8476438 34611511

[24] GBIF.org [Internet] (2022) GBIF

[25] Maa TC (1967) A synopsis of Diptera Pupipara of Japan. Pacif 9(4):727–760

[26] GBIF. GBIF Backbone Taxonomy. Checklist dataset. 2023 [cited 2025 Jan 22]. Ornithomya candida Maa (1967) Available from: 10.15468/39omei

[27] Wikipedia [Internet] (2024) [cited 2024 May 5]. Sergy Paramonov (entomologist). Available from: https://en.wikipedia.org/wiki/Sergey_Paramonov_(entomologist)

[28] Maa TC (1965) On the name Ornithomya vs. Ornithomyia J Med Ent 1(4):394 10.1093/jmedent/1.4.394 14280493

[29] French A (2022) The colouring of John Curtis’s British entomology (1834–1839): Joseph Standish and the Paragon of perfection. Arch Nat Hist 49(1):62–77

[30] Wikipedia (2024) John Curtis (entomologist). https://en.wikipedia.org/wiki/John_Curtis_(entomologist

[31] Petersen FT, Damgaard J, Meier RDNA, Taxonomy (2007) How many DNA Sequences are needed for solving a Taxonomic Problem? The Case of two Parapatric Species of Louse Flies (Diptera: Hippoboscidae: Ornithomya Latreille, 1802). Arthropod Syst Phylogeny [Internet].;65(2):111–7. Available from: https://www.researchgate.net/publication/237303838_DNA_Taxonomy_How_many_DNA_Sequences_are_needed_for_solving_a_Taxonomic_Problem_The_Case_of_two_Parapatric_Species_of_Louse_Flies_Diptera_Hippoboscidae_Ornithomya_Latreille_1802

[32] Petersen FT, Meier R, Kutty SN, Wiegmann BM (2007) The phylogeny and evolution of host choice in the Hippoboscoidea (Diptera) as reconstructed using four molecular markers. Mol Phylogenet Evol [Internet].;45(1):111–22. Available from: https://pubmed.ncbi.nlm.nih.gov/17583536/#:~:text=Thephylogenyandevolutionofhostchoicein,ofbatflies%2CtheStreblidaeandtheNycteribiidae 10.1016/j.ympev.2007.04.023 17583536

[33] Oboňa J, Fogašová K, Fulín M, Greš S, Manko P, Repaský J et al (2022) Updated taxonomic keys for European Hippoboscidae (Diptera), and expansion in Central Europe of the bird louse fly Ornithomya comosa (Austen, 1930) with the first record from Slovakia. Zookeys [Internet].;1115:81–101. Available from: https://zookeys.pensoft.net/article/80146/10.3897/zookeys.1115.80146 PMC9848778 36761073

[34] Curaera [Internet]. 2022 [cited 2024 May 13]. Available from: https://www.cucaera.co.uk/grp/?refs=%26map=ordnance%26vcs=true%26graticule=true%26nsgrids=false%26zoom=5=53.30462107510271%26lon=0.6811523437500001%26distinct=false%26bedrock=false%26superficial=false%26sketches=

[35] Batch Convert Tool [Internet] (2022) [cited 2022 Jul 25]. Available from: https://gridreferencefinder.com/batchConvert/batchConvert.php

[36] Hunter B Batch Coordinate Converter [Internet]. 2022 [cited 2022 Jul 25]. Available from: http://ww2.scenic-tours.co.uk/serve.php?t=WoNlbJvoVlhuJL5405objaa8jVO8atNuwZV

[37] BTO IS and Demography Team. BTO Ringers Info [Internet]. British Trust for Ornithology (2016) Available from: https://www.bto.org/about-bto/press-releases/app-aid-ringers

[38] R Development Core Team. R: A language and environment for statistical computing. [Internet]. R Foundation for Statistical Computing, Vienna, Austria (2022) Available from: https://www.r-project.org/

[39] Wickham H, Francois R, Henry L, Muller K, Package (2023) ’dplyr’: A Grammar of Data Manipulation [Internet]. Available from: https://cran.r-project.org/package=dplyr

[40] Grolemund G, Wickham H (2011) Dates and Times Made Easy with lubridate. J Stat Softw [Internet].;40(3):1–25. Available from: https://www.jstatsoft.org/v40/i03/

[41] Kassambara A, Mundt F, factoextra (2020) Extract and Visualize the Results of Multivariate Data Analyses. R package version 1.0.1 [Internet]. Available from: https://rpkgs.datanovia.com/factoextra/index.html

[42] Ripley B, Venables B, Bates DM, Hornik K, Gebhardt A, Firth D, Package ‘ (2024) MASS.’; Available from: http://www.stats.ox.ac.uk/pub/MASS4/ Contact

[43] Wickham H, Chang W, Henry L, Pedersen TL, Takahashi K, Wilke C et al (2023) Package ‘ggplot2’: Elegant Graphics for Data [Internet]. New York: Springer-Verlag; Available from: https://ggplot2.tidyverse.org

[44] Wawman DC, University of Oxford and Wytham Woods Genome Acquisition Lab (2025), Darwin Tree of Life Barcoding collective, Wellcome Sanger Institute Tree of Life Management: Samples Laboratory Team, Wellcome Sanger Institute Scientific Operations: Sequencing Operations, Wellcome Sanger Institute Tree of Life Core Informatics team, The genome sequence of the Finch Louse Fly, Ornithomya fringillina (Curtis, 1836). Wellcome Open Res [Internet].;10:185. Available from: https://wellcomeopenresearch.org/articles/10-185/v1

[45] Blaxter M, Mieszkowska N, Di Palma F, Holland P, Durbin R, Richards T et al (2022) Sequence locally, think globally: The Darwin Tree of Life Project. Proc Natl Acad Sci [Internet].;119(4):1–7. Available from: 10.1073/pnas.2115642118 PMC8797607 35042805

[46] UK Barcode of Life [Internet] 2022 [cited 2024 Feb 20]. Available from: https://www.ukbol.org/

[47] Chen S, Zhou Y, Chen Y, Gu J (2018) fastp: an ultra-fast all-in-one FASTQ preprocessor. Bioinformatics [Internet].;34(17):i884–90. Available from: https://academic.oup.com/bioinformatics/article/34/17/i884/5093234 10.1093/bioinformatics/bty560 PMC6129281 30423086

[48] Ewels P, Magnusson M, Lundin S, Käller M (2016) MultiQC: summarize analysis results for multiple tools and samples in a single report. Bioinformatics [Internet].;32(19):3047–8. Available from: https://academic.oup.com/bioinformatics/article/32/19/3047/2196507 10.1093/bioinformatics/btw354 PMC5039924 27312411

[49] Langmead B, Salzberg SL (2012) Fast gapped-read alignment with Bowtie 2. Nat Methods [Internet].;9(4):357–9. Available from: https://www.nature.com/articles/nmeth.1923 10.1038/nmeth.1923 PMC3322381 22388286

[50] Li D, Liu CM, Luo R, Sadakane K, Lam TW (2015) MEGAHIT: an ultra-fast single-node solution for large and complex metagenomics assembly via succinct de Bruijn graph. Bioinformatics [Internet].;31(10):1674–6. Available from: https://academic.oup.com/bioinformatics/article/31/10/1674/177884 10.1093/bioinformatics/btv033 25609793

[51] Wood DE, Lu J, Langmead B (2019) Improved metagenomic analysis with Kraken 2. Genome Biol [Internet].;20(1):257. Available from: https://genomebiology.biomedcentral.com/articles/10.1186/s13059-019-1891-0 10.1186/s13059-019-1891-0 PMC6883579 31779668

[52] Lu J, Breitwieser FP, Thielen P, Salzberg SL (2017) Bracken: estimating species abundance in metagenomics data. PeerJ Comput Sci [Internet].;3(1):e104. Available from: https://peerj.com/articles/cs-104 10.7717/peerj-cs.104 PMC12016282 40271438

[53] Camacho C, Coulouris G, Avagyan V, Ma N, Papadopoulos J, Bealer K et al (2009) BLAST+: architecture and applications. BMC Bioinformatics [Internet].;10(1):421. Available from: https://bmcbioinformatics.biomedcentral.com/articles/10.1186/1471-2105-10-421 10.1186/1471-2105-10-421 PMC2803857 20003500

[54] Charif D, Lobry JR (2007) Seqin{R} 1.0–2: a contributed package to the {R} project for statistical computing devoted to biological sequences retrieval and analysis. [Internet]. New York: Springer-Verlag; Available from: http://seqinr.r-forge.r-project.org/

[55] Paradis E, Schiep K (2019) ape 5.0: an enviroment for modern pylogenetics and evolutionary analyses in R. Bioinformatics 35:526–528. 10.1093/bioinformatics/bty633 10.1093/bioinformatics/bty633 30016406

[56] Jombart T, Adegenet (2008) A R package for the multivariate analysis of genetic markers. Bioinformatics 24(11):1403–1405 18397895 10.1093/bioinformatics/btn129

[57] Garnier S (2024) Package ‘ viridis ’: Colorblind friendly color maps for R.; Available from: https://sjmgarnier.github.io/viridis/

[58] Muller K, Wickham H, James DA, Falcon S (2023) RSQLite: SQLite interface for R

[59] Wright ES (2014) Get Started DECIPHERing 3:1–9

[60] Ronquist F, Teslenko M, van der Mark P, Ayres DL, Darling A, Höhna S et al (2012) MrBayes 3.2: Efficient Bayesian Phylogenetic Inference and Model Choice Across a Large Model Space. Syst Biol [Internet].;61(3):539–42. Available from: https://academic.oup.com/sysbio/article/61/3/539/1674894 10.1093/sysbio/sys029 PMC3329765 22357727

[61] Kumar S, Stecher G, Suleski M, Sanderford M, Sharma S, Tamura K (2024) MEGA12: Molecular Evolutionary Genetic Analysis Version 12 for Adaptive and Green Computing. Battistuzzi FU, editor. Mol Biol Evol [Internet].;41(12). Available from: https://academic.oup.com/mbe/article/doi/10.1093/molbev/msae263/7930299 10.1093/molbev/msae263 PMC11683415 39708372

[62] Tamura K, Nei M (1993) Estimation of the number of nucleotide substitutions in the control region of mitochondrial DNA in humans and chimpanzees. Mol Biol Evol 10:512–526 8336541 10.1093/oxfordjournals.molbev.a040023

[63] Hargrove JW (2020) A model for the relationship between wing fray and chronological and ovarian ages in tsetse (Glossina spp). Med Vet Entomol [Internet].;34(3):251–63. Available from: https://resjournals.onlinelibrary.wiley.com/doi/10.1111/mve.12439 10.1111/mve.12439 32222085

[64] Beutler M, Hart A, Hall MJR (2020) The use of wing fray and sex ratios to determine the origin of flies at an indoor crime scene. Forensic Sci Int [Internet].;307:110104. Available from: 10.1016/j.forsciint.2019.110104 10.1016/j.forsciint.2019.110104 31918163

