# Supplementary material for: Clarifying the Taxonomy of the Finch Louse Fly Ornithomya Fringillina (Curtis) (Diptera: Hippoboscidae) – An Analysis of Morphotypes
Source: Acta Parasitol. 2025 Aug 8;70(4):175. doi: 10.1007/s11686-025-01113-z (PMC12334518; doi:10.1007/s11686-025-01113-z)
Supplement: Supplementary file 1 — Supplementary Material 1 [file 11686_2025_1113_MOESM1_ESM.docx]

Online Resource 1: Table S1. Specimens of *Ornithomya fringillina* examined.

| **No.** | | **Species** | | | | **Country** | **Sex** | **County** | **Morphotype** | **Location** | **Altitude** | **Date** | **Host species** | **Host binomial** | **Collected by** |
| --- | --- | --- | --- | --- | --- | --- | --- | --- | --- | --- | --- | --- | --- | --- | --- |
| 121 | | O.fringillina | | | | UNITED KINGDOM | ♀ | Cumbria | 3 | Watchtree Wetlands | 65 | 21.vii.2020 | Chaffinch | *Fringilla coelebs* | Watchtree RG |
| 122 | | O.fringillina | | | | UNITED KINGDOM | ♂ | Cumbria |  | Watchtree Wetlands | 65 | 21.vii.2020 | Reed Warbler | *Acrocephalus scirpaceus* | Watchtree RG |
| 125 | | O.fringillina | | | | UNITED KINGDOM |  | Cumbria | 2 | Watchtree | 73 | 20.ix.2020 | Dunnock | *Prunella modularis* | Watchtree RG |
| 129 | | O.fringillina | | | | UNITED KINGDOM | ♀ | Cumbria |  | Watchtree | 70 | 19.viii.2021 | redpoll | *Acanthis sp.* | Watchtree RG |
| 174 | | O.fringillina | | | | UNITED KINGDOM |  | County Durham | 3 | Butterknowle | 208 | 24.vii.2020 | Chaffinch | *Fringilla coelebs* | John Black |
| 175 | | O.fringillina | | | | UNITED KINGDOM |  | County Durham | 3 | Butterknowle | 208 | 3.viii.2020 | Dunnock | *Prunella modularis* | John Black |
| 177 | | O.fringillina | | | | UNITED KINGDOM | ♂ | County Durham | 3 | Butterknowle | 208 | 3.viii.2020 | Robin | *Erithacus rubecula* | John Black |
| 192 | | O.fringillina | | | | UNITED KINGDOM | ♀ | East Riding of Yorkshire | 3 | Flamborough | 45 | 18.vii.2021 | Dunnock | *Prunella modularis* | Andy Hood |
| 201 | | O.fringillina | | | | UNITED KINGDOM | ♀ | County Durham | 3 | Butterknowle | 208 | 18.viii.2020 | Dunnock | *Prunella modularis* | John Black |
| 202 | | O.fringillina | | | | UNITED KINGDOM | ♀ | County Durham | 3 | Butterknowle | 208 | 18.viii.2020 |  |  | John Black |
| 204 | | O.fringillina | | | | UNITED KINGDOM | ♀ | County Durham | 3 | Butterknowle | 208 | 18.viii.2020 | Goldfinch | *Carduelis carduelis* | John Black |
| 207 | | O.fringillina | | | | UNITED KINGDOM | ♀ | County Durham | 3 | Butterknowle | 208 | 24.viii.2020 | Willow Tit | *Poecile montanus* | John Black |
| 208 | | O.fringillina | | | | UNITED KINGDOM | ♀ | County Durham | 3 | Butterknowle | 208 | 24.viii.2020 | Great Tit | *Parus major* | John Black |
| 285 | | O.fringillina | | | | UNITED KINGDOM | ♂ | Norfolk | 3 | Cranwich | 10 | 12.ix.2020 | Long-tailed Tit | *Aegithalos caudatus* | Lee Barber |
| 343 | | O.fringillina | | | | UNITED KINGDOM | ♀ | Warwickshire | 3 | Whiteacre Heath | 72 | 1.ix.2021 |  |  | Dave Clifton |
| 347 | | O.fringillina | | | | UNITED KINGDOM | ♂ | Warwickshire | 2 | Whiteacre Heath | 72 | 17.viii.2021 | Reed Warbler | *Acrocephalus scirpaceus* | Dave Clifton |
| 355 | | O.fringillina | | | | UNITED KINGDOM |  | Staffordshire | damaged | Walsall | 139 | 23.x.2021 | Goldfinch | *Carduelis carduelis* | Dave Clifton |
| 421 | | O.fringillina | | | | UNITED KINGDOM |  | Berkshire | 3 | Wraysbury | 17 | 4.viii.2020 | Robin | *Erithacus rubecula* | Denise Lamsdell |
| 424 | | O.fringillina | | | | UNITED KINGDOM | ♀ | Greater London |  | South Harefield, Broadwater | 36 | 28.vii.2021 | Chiffchaff | *Phylloscopus collybita* | Denise Lamsdell |
| 425 | | O.fringillina | | | | UNITED KINGDOM | ♀ | Greater London | 3 | South Harefield, Broadwater | 36 | 28.vii.2021 | Robin | *Erithacus rubecula* | Denise Lamsdell |
| 461 | | O.fringillina | | | | UNITED KINGDOM | ♂ | Northamptonshire | 2 | Harrington | 162 | 2.viii.2020 | Yellowhammer | *Emberiza citrinella* | Chris Payne |
| 483 | | O.fringillina | | | | IRELAND |  | Wexford | 3 | Kilpierce | 55 | 11.xii.2020 | Coal Tit | *Periparus ater* | Mark Stanley |
| 561 | | O.fringillina | | | | UNITED KINGDOM | ♀ | Northamptonshire | 3 | Maidwell | 135 | 5.ix.2020 | Chaffinch | *Fringilla coelebs* | Helen Franklin |
| 637 | | O.fringillina | | | | UNITED KINGDOM |  | Gloucestershire | 3 | Bristol, Brenty | 61 | 18.ix.2021 | House Sparrow | *Passer domesticus* | Mark Dadds |
| 638 | | O.fringillina | | | | UNITED KINGDOM | ♂ | Gloucestershire | 2 | Bristol, Brenty | 61 | 16.viii.2021 | Goldfinch | *Carduelis carduelis* | Mark Dadds |
| 645 | | O.fringillina | | | | UNITED KINGDOM |  | Buckinghamshire | 2 | Marsworth | 125 | 2.ix.2020 | Robin | *Erithacus rubecula* | Lynne Lambert |
| 667 | | O.fringillina | | | | UNITED KINGDOM |  | Norfolk | 3 | Walsey Hill | 8 | 6.viii.2020 | Dunnock | *Prunella modularis* | Norfolk Ornithologists Association |
| 670 | | O.fringillina | | | | UNITED KINGDOM |  | Norfolk | 3 | Walsey Hill | 8 | 6.viii.2020 | Whitethroat | *Curruca communis* | Norfolk Ornithologists Association |
| 674 | | O.fringillina | | | | UNITED KINGDOM | ♀ | Norfolk | 3 | Walsey Hill | 8 | 16.viii.2020 | Greenfinch | *Chloris chloris* | Norfolk Ornithologists Association |
| 1068 | | O.fringillina | | | | UNITED KINGDOM | ♀ | Essex | 3 | Rayne | 69 | 17.vii.2021 | House Sparrow | *Passer domesticus* | Jenny Dunn |
| 1083 | | O.fringillina | | | | UNITED KINGDOM | ♀ | Shropshire | 2 | Whixall Moss | 90 | 31.x.2021 | Lesser Redpoll | *Acanthis cabaret* | Bob Harris |
| 1109 | | O.fringillina | | | | UNITED KINGDOM | ♂ | Lincolnshire | 3 | Nocton Fen, Wasps Nest | 8 | 12.x.2021 | Chaffinch | *Fringilla coelebs* | Carl Soulsbury |
| 1118 | | O.fringillina | | | | UNITED KINGDOM | ♂ | Nottinghamshire | 2 | Bevercotes Lagoon | 20 | 25.vii.2021 | Reed Warbler | *Acrocephalus scirpaceus* | Carl Soulsbury |
| 1281 | | O.fringillina | | | | UNITED KINGDOM | ♀ | Dumfries and Galloway | 3 | Cumrue | 58 | 5.ix.2021 | Reed Bunting | *Emberiza schoeniclus* | Ronnie Graham |
| 1289 | | O.fringillina | | | | UNITED KINGDOM | ♂ | Dumfries and Galloway | 3 | Rangebank | 48 | 12.viii.2021 | Treecreeper | *Certhia familiaris* | Ronnie Graham |
| 1305 | | O.fringillina | | | | ISLE OF MAN | ♂ |  | 2 | Lower Ballabeg | 60 | 19.viii.2021 | Goldcrest | *Regulus regulus* | Calf of Man Bird Observatory |
| 1306 | | O.fringillina | | | | ISLE OF MAN |  |  | 3 | Calf of Man | 80 | 23.x.2021 | Chaffinch | *Fringilla coelebs* | Calf of Man Bird Observatory |
| 1307 | | O.fringillina | | | | ISLE OF MAN |  |  | 3 | Calf of Man | 80 | 25.x.2021 | Goldfinch | *Carduelis carduelis* | Calf of Man Bird Observatory |
| 1444 | | O.fringillina | | | | UNITED KINGDOM | ♀ | Pembrokeshire | 2 | Skokholm Island | 35 | 14.ix.2021 | Robin | *Erithacus rubecula* | Skokholm Bird Observatory |
| 1532 | | O.fringillina | | | | UNITED KINGDOM |  | Isle of Wight | damaged | Bembridge | 15 | 28.x.2021 | Goldfinch | *Carduelis carduelis* | Isle of Wight Ringing Group |
| 1533 | | O.fringillina | | | | UNITED KINGDOM |  | Isle of Wight | damaged | Bembridge | 15 | 4.xi.2021 | Goldfinch | *Carduelis carduelis* | Isle of Wight Ringing Group |
| 1541 | | O.fringillina | | | | UNITED KINGDOM |  | Northamptonshire | 3 | Stanford Reservoir | 115 | 4.ix.2021 | Blackcap | *Sylvia atricapilla* | Stanford RG (David Neal) |
| 1561 | | O.fringillina | | | | UNITED KINGDOM |  | Northamptonshire |  | Stanford Reservoir | 115 | 29.viii.2021 |  |  | Stanford RG |
| 1564 | | O.fringillina | | | | UNITED KINGDOM | ♀ | Northamptonshire |  | Stanford Reservoir | 115 | 21.ix.2021 | Blackcap | *Sylvia atricapilla* | Stanford RG |
| 1629 | | O.fringillina | | | | UNITED KINGDOM | ♀ | Isle of Arran | 3 | Corriecravie | 1 | 10.viii.2021 | Robin | *Erithacus rubecula* | Clyde RG |
| 1632 | | O.fringillina | | | | UNITED KINGDOM | ♂ | Isle of Arran | 3 | Corriecravie | 1 | 1.viii.2021 | Robin | *Erithacus rubecula* | Clyde RG |
| 1640 | | O.fringillina | | | | UNITED KINGDOM |  | Isle of Arran | 3 | Corriecravie | 1 | 29.viii.2021 | Stonechat | *Saxicola rubicola* | Clyde RG |
| 1681 | | O.fringillina | | | | UNITED KINGDOM |  | Powys | 3 | Nantmel | 215 | 16.x.2021 | Dunnock | *Prunella modularis* | Mid-Wales Ringing Group |
| 1695 | | O.fringillina | | | | UNITED KINGDOM |  | Powys | 3 | Nantmel | 215 | 23.viii.2021 | Chaffinch | *Fringilla coelebs* | Mid-Wales Ringing Group |
| 1697 | | O.fringillina | | | | UNITED KINGDOM | ♀ | Powys | 3 | Nantmel | 215 | 20.viii.2021 | Great Tit | *Parus major* | Mid-Wales Ringing Group |
| 1698 | | O.fringillina | | | | UNITED KINGDOM | ♀ | Ceredigion | 3 | Aberystwyth, Ynyslas, Borth Bog | 2 | 22.viii.2021 | Sedge Warbler | *Acrocephalus schoenobaenus* | Mid-Wales Ringing Group |
| 1700 | | O.fringillina | | | | UNITED KINGDOM | ♂ | Ceredigion | 2 | Aberystwyth, Ynyslas, Borth Bog | 2 | 22.viii.2021 | Reed Warbler | *Acrocephalus scirpaceus* | Mid-Wales Ringing Group |
| 1787 | | O.fringillina | | | | UNITED KINGDOM | ♀ | North Yorkshire | 2&3 | Ripon, Ripon Parks | 40 | 4.ix.2021 | Reed Bunting | *Emberiza schoeniclus* | East Dales Ringing Group |
| 1861 | | O.fringillina | | | | UNITED KINGDOM |  | Leicestershire | 2 | Stanford Reservoir | 110 | 17.vii.2021 | Swallow | *Hirundo rustica* | Stanford Ringing Group |
| 1863 | | O.fringillina | | | | UNITED KINGDOM |  | Kent | 2 | Dungeness Bird Observatory | 6 | 23.vii.2021 | Dunnock | *Prunella modularis* | Stanford Ringing Group |
| 1864 | | O.fringillina | | | | UNITED KINGDOM | ♀ | Leicestershire | 3 | Stanford Reservoir | 110 | 19.viii.2021 | Swallow | *Hirundo rustica* | Stanford Ringing Group |
| 1963 | | O.fringillina | | | | UNITED KINGDOM | ♂ | Hertfordshire | 3 | Greys | 120 | 16.vii.2021 | Blackcap | *Sylvia atricapilla* | Ken Griffin |
| 1986 | | O.fringillina | | | | UNITED KINGDOM |  | Stirlingshire |  | Falkirk | 115 | 16.viii.2021 | House Sparrow | *Passer domesticus* | Liam Reid |
| 2196 | | O.fringillina | | | | UNITED KINGDOM | ♀ | Norfolk | 3 | Holme, Holme Bird Observatory | 2 | 16.ix.2021 | Reed Warbler | *Acrocephalus scirpaceus* | Norfolk Ornithologists' Association |
| 2221 | | O.fringillina | | | | UNITED KINGDOM | ♀ | Wiltshire | 3 | Warminster, Battle | 148 | 1.viii.2021 | Whitethroat | *Curruca communis* | ANON |
| 2222 | | O.fringillina | | | | UNITED KINGDOM | ♂ | Wiltshire | 2 | New Zealand Farm | 197 | 18.viii.2021 | Blackbird | *Turdus merula* | ANON |
| 2223 | | O.fringillina | | | | UNITED KINGDOM | ♂ | Wiltshire | 2 | New Zealand Farm | 197 | 18.viii.2021 | Yellowhammer | *Emberiza citrinella* | ANON |
| 2224 | | O.fringillina | | | | UNITED KINGDOM | ♂ | Wiltshire | 2 | Warminster, Battle | 148 | 27.viii.2021 | Yellowhammer | *Emberiza citrinella* | ANON |
| 2225 | | O.fringillina | | | | UNITED KINGDOM | ♀ | Wiltshire | 2 | Warminster, Battle | 148 | 27.viii.2021 | Yellowhammer | *Emberiza citrinella* | ANON |
| 2261 | | O.fringillina | | | | UNITED KINGDOM | ♀ | Norfolk | 3 | Holme, Holme Bird Observatory | 2 | 21.viii.2021 | Reed Warbler | *Acrocephalus scirpaceus* | Norfolk Ornithologists' Association |
| 2262 | | O.fringillina | | | | UNITED KINGDOM |  | Norfolk | 2 | Holme, Holme Bird Observatory | 2 | 22.viii.2021 | Reed Warbler | *Acrocephalus scirpaceus* | Norfolk Ornithologists' Association |
| 2263 | | O.fringillina | | | | UNITED KINGDOM | ♂ | Norfolk | 2 | Holme, Holme Bird Observatory | 2 | 27.viii.2021 | Whitethroat | *Curruca communis* | Norfolk Ornithologists' Association |
| 2264 | | O.fringillina | | | | UNITED KINGDOM | ♀ | Norfolk | 2 | Holme, Holme Bird Observatory | 2 | 21.ix.2021 | Reed Warbler | *Acrocephalus scirpaceus* | Norfolk Ornithologists' Association |
| 2403 | | O.fringillina | | | | UNITED KINGDOM | ♂ | Cheshire | 3 | Hoylake | 6 | 21.vii.2021 | House Sparrow | *Passer domesticus* | Jane Turner |
| 2412 | | O.fringillina | | | | UNITED KINGDOM |  | Cheshire | 3 | Hoylake | 6 | 26.viii.2021 | House Sparrow | *Passer domesticus* | Jane Turner |
| 2413 | | O.fringillina | | | | UNITED KINGDOM |  | Cheshire | 3 | Hoylake | 6 | 27.viii.2021 | Goldfinch | *Carduelis carduelis* | Jane Turner |
| 2414 | | O.fringillina | | | | UNITED KINGDOM |  | Cheshire | 3 | Hoylake | 6 | 10.xi.2021 | Dunnock | *Prunella modularis* | Jane Turner |
| 2495 | | O.fringillina | | | | UNITED KINGDOM |  | Dorset | 2 | Portland Bird Observatory | 16 | 7.viii.2021 | Whitethroat | *Curruca communis* | Portland Bird Observatory |
| 2602 | | O.fringillina | | | | UNITED KINGDOM | ♀ | Inverness-shire | 3 | Carse of Ardersier | 5 | 21.vii.2021 | Willow Warbler | *Phylloscopus trochilus* | Hugh Insley |
| 2606 | | O.fringillina | | | | UNITED KINGDOM | ♂ | Inverness-shire | 3 | Carse of Ardersier | 5 | 21.vii.2021 | Blackcap | *Sylvia atricapilla* | Hugh Insley |
| 2607 | | O.fringillina | | | | UNITED KINGDOM | ♂ | Inverness-shire | 3 | Inverness, Drummond | 34 | 19.vii.2021 | Great Tit | *Parus major* | Hugh Insley |
| 2609 | | O.fringillina | | | | UNITED KINGDOM | ♀ | Inverness-shire | 3 | Carse of Ardersier | 5 | 21.vii.2021 | Willow Warbler | *Phylloscopus trochilus* | Hugh Insley |
| 2613 | | O.fringillina | | | | UNITED KINGDOM |  | Inverness-shire | 3 | Inverness, Drummond | 34 | 20.vii.2021 | Bullfinch | *Pyrrhula pyrrhula* | Hugh Insley |
| 2614 | | O.fringillina | | | | UNITED KINGDOM | ♂ | Inverness-shire | 3 | Inverness, Drummond | 34 | 19.vii.2021 | Dunnock | *Prunella modularis* | Hugh Insley |
| 2619 | | O.fringillina | | | | UNITED KINGDOM | ♀ | Inverness-shire | 3 | Inverness, Drummond | 34 | 16.vii.2021 | Siskin | *Spinus spinus* | Hugh Insley |
| 2762 | | O.fringillina | | | | UNITED KINGDOM | ♂ | Wiltshire | 3 | Longbridge Deverill, The Marsh | 120 | 13.vii.2021 | Blackcap | *Sylvia atricapilla* | R. H. Creighton |
| 2763 | | O.fringillina | | | | UNITED KINGDOM | ♀ | Wiltshire | 3 | Longbridge Deverill, The Marsh | 120 | 17.viii.2021 | Blackcap | *Sylvia atricapilla* | R. H. Creighton |
| 2781 | | O.fringillina | | | | UNITED KINGDOM | ♀ | Lincolnshire | 3 | Wainfleet | 0 | 24.viii.2021 | Oystercatcher | *Haematopus ostralegus* | Rachel Taylor |
| 2788 | | O.fringillina | | | | UNITED KINGDOM | ♀ | Anglesey | 2 | Cors Ddyga | 4 | 14.viii.2021 | Reed Warbler | *Acrocephalus scirpaceus* | Rachel Taylor |
| 2809 | | O.fringillina | | | | UNITED KINGDOM |  | Hampshire | 3 | Hayling Island, Hayling Golf Course | 2 | 1.viii.2021 | Whitethroat | *Curruca communis* | Trevor Codlin |
| 2813 | | O.fringillina | | | | UNITED KINGDOM | ♂ | Hampshire | 3 | Funtley | 16 | 11.vi.2021 | Blue Tit | *Cyanistes caeruleus* | Trevor Codlin |
| 2814 | | O.fringillina | | | | UNITED KINGDOM | ♀ | Hampshire | 3 | Funtley | 16 | 11.vi.2021 | Blue Tit | *Cyanistes caeruleus* | Trevor Codlin |
| 2861 | | O.fringillina | | | | UNITED KINGDOM |  | Fife | 3 | Bow of Fife | 100 | 3.x.2021 | Dunnock | *Prunella modularis* | Dr C. McGuigan |
| 2884 | | O.fringillina | | | | UNITED KINGDOM | ♂ | Dumfries and Galloway | 3 | Stranraer, Leswalt | 35 | 15.viii.2021 | Goldfinch | *Carduelis carduelis* | Geoff & Jean Sheppard |
| 2885 | | O.fringillina | | | | UNITED KINGDOM | ♀ | Dumfries and Galloway | 3 | Stranraer, Leswalt | 35 | 15.viii.2021 | Goldfinch | *Carduelis carduelis* | Geoff & Jean Sheppard |
| 2889 | | O.fringillina | | | | UNITED KINGDOM | ♂ | Dumfries and Galloway | 3 | Stranraer, Leswalt | 35 | 20.viii.2021 | Chaffinch | *Fringilla coelebs* | Geoff & Jean Sheppard |
| 2890 | | O.fringillina | | | | UNITED KINGDOM | ♂ | Dumfries and Galloway | 3 | Stranraer, Leswalt | 35 | 20.viii.2021 |  |  | Geoff & Jean Sheppard |
| 2892 | | O.fringillina | | | | UNITED KINGDOM |  | Dumfries and Galloway | 3 | Stranraer, Leswalt | 35 | 23.viii.2021 | Chaffinch | *Fringilla coelebs* | Geoff & Jean Sheppard |
| 2895 | | O.fringillina | | | | UNITED KINGDOM | ♀ | Dumfries and Galloway | 3 | Stranraer, Leswalt | 35 | 27.viii.2021 | Blue Tit | *Cyanistes caeruleus* | Geoff & Jean Sheppard |
| 2896 | | O.fringillina | | | | UNITED KINGDOM | ♂ | Dumfries and Galloway | 3 | Stranraer, Leswalt | 35 | 3.ix.2021 | Robin | *Erithacus rubecula* | Geoff & Jean Sheppard |
| 2898 | | O.fringillina | | | | UNITED KINGDOM |  | Dumfries and Galloway | 3 | Stranraer, Leswalt | 35 | 8.ix.2021 | Goldfinch | *Carduelis carduelis* | Geoff & Jean Sheppard |
| 2900 | | O.fringillina | | | | UNITED KINGDOM | ♀ | Dumfries and Galloway | 3 | Stranraer, Leswalt | 35 | 20.ix.2021 | House Sparrow | *Passer domesticus* | Geoff & Jean Sheppard |
| 2983 | | O.fringillina | | | | UNITED KINGDOM |  | Norfolk | 3 | Walsey Hill | 8 | 20.viii.2021 | Blackcap | *Sylvia atricapilla* | Norfolk Ornithologists Association |
| 2989 | | O.fringillina | | | | UNITED KINGDOM | ♀ | Norfolk | 3 | Walsey Hill | 8 | 19.viii.2021 | Dunnock | *Prunella modularis* | Norfolk Ornithologists Association |
| 2991 | | O.fringillina | | | | UNITED KINGDOM | ♀ | Norfolk | 3 | Hempton Marsh | 36 | 28.ix.2021 | Robin | *Erithacus rubecula* | Norfolk Ornithologists Association |
| 2997 | | O.fringillina | | | | UNITED KINGDOM |  | Norfolk | 3 | Walsey Hill | 8 | 2.viii.2021 | Reed Warbler | *Acrocephalus scirpaceus* | Norfolk Ornithologists Association |
| 2998 | | O.fringillina | | | | UNITED KINGDOM | ♂ | Norfolk | 3 | Walsey Hill | 8 | 20.viii.2021 | Blackcap | *Sylvia atricapilla* | Norfolk Ornithologists Association |
| 3101 | | O.fringillina | | | | UNITED KINGDOM | ♀ | Somerset | 2 | Portishead, Gordano Valley | 0 | 20.viii.2021 | Robin | *Erithacus rubecula* | Gordano Valley RG |
| 3102 | | O.fringillina | | | | UNITED KINGDOM |  | Somerset | 2 | Portishead, Gordano Valley | 0 | 26.ix.2021 | Blackcap | *Sylvia atricapilla* | Gordano Valley RG |
| 3182 | | O.fringillina | | | | UNITED KINGDOM | ♂ | Devon | 2 | Ilfracombe, West Down, Buttercombe Barton | 160 | 19.vi.2021 | Bullfinch | *Pyrrhula pyrrhula* | Chris Dee |
| 3192 | | O.fringillina | | | | UNITED KINGDOM | ♂ | Devon | 2 | Ilfracombe, West Down, Buttercombe Barton | 160 | 15.vii.2021 | Siskin | *Spinus spinus* | Chris Dee |
| 3200 | | O.fringillina | | | | UNITED KINGDOM | ♂ | Devon | 3 | Ilfracombe, West Down, Buttercombe Barton | 160 | 2.viii.2021 |  |  | Chris Dee |
| 3364 | | O.fringillina | | | | UNITED KINGDOM |  | Kent | damaged | Sandwich Bay | 3 | 16.x.2021 | Goldfinch | *Carduelis carduelis* | Sandwich Bay Bird Observatory |
| 3406 | | O.fringillina | | | | UNITED KINGDOM | ♀ | County Durham | 3 | Butterknowle | 208 | 11.vii.2021 | House Sparrow | *Passer domesticus* | John Black |
| 3414 | | O.fringillina | | | | UNITED KINGDOM | ♀ | County Durham | 3 | Butterknowle | 208 | 5.viii.2021 | Dunnock | *Prunella modularis* | John Black |
| 3415 | | O.fringillina | | | | UNITED KINGDOM | ♂ | County Durham |  | Butterknowle | 208 | 5.viii.2021 | Dunnock | *Prunella modularis* | John Black |
| 3416 | | O.fringillina | | | | UNITED KINGDOM | ♀ | County Durham | 3 | Butterknowle | 208 | 5.viii.2021 | Blue Tit | *Cyanistes caeruleus* | John Black |
| 3417 | | O.fringillina | | | | UNITED KINGDOM | ♂ | County Durham | 3 | Butterknowle | 208 | 5.viii.2021 | Robin | *Erithacus rubecula* | John Black |
| 3418 | | O.fringillina | | | | UNITED KINGDOM | ♂ | County Durham | 3 | Butterknowle | 208 | 5.viii.2021 | House Sparrow | *Passer domesticus* | John Black |
| 3419 | | O.fringillina | | | | UNITED KINGDOM | ♂ | County Durham | 3 | Butterknowle | 208 | 5.viii.2021 | Chaffinch | *Fringilla coelebs* | John Black |
| 3581 | | O.fringillina | | | | UNITED KINGDOM | ♀ | County Durham | 3 | Butterknowle | 208 | 5.viii.2021 | House Sparrow | *Passer domesticus* | John Black |
| 3584 | | O.fringillina | | | | UNITED KINGDOM | ♂ | County Durham | 2 | Butterknowle | 208 | 19.viii.2021 | House Sparrow | *Passer domesticus* | John Black |
| 3585 | | O.fringillina | | | | UNITED KINGDOM |  | County Durham | 3 | Butterknowle | 208 | 24.viii.2021 | Chaffinch | *Fringilla coelebs* | John Black |
| 3587 | | O.fringillina | | | | UNITED KINGDOM |  | County Durham | 3 | Butterknowle | 208 | 24.viii.2021 | Blue Tit | *Cyanistes caeruleus* | John Black |
| 3589 | | O.fringillina | | | | UNITED KINGDOM |  | County Durham | 3 | Butterknowle | 208 | 30.viii.2021 |  |  | John Black |
| 3881 | | O.fringillina | | | | UNITED KINGDOM | ♀ | West Sussex | 3 | Cissbury | 97 | 3.x.2021 | Robin | *Erithacus rubecula* | Steyning RG |
| 3929 | | O.fringillina | | | | UNITED KINGDOM |  | Bedfordshire | 2 | Sandy, Waterloo Thorns | 21 | 29.viii.2021 | Blackcap | *Sylvia atricapilla* | Sara Miller |
| 4101 | | O.fringillina | | | | UNITED KINGDOM | ♂ | Highland | 2&3 | Fortrose | 20 | 12.ix.2021 | Great Tit | *Parus major* | Alister Clunas |
| 4102 | | O.fringillina | | | | UNITED KINGDOM |  | Highland | 3 | Fortrose | 20 | 18.ix.2021 | Goldfinch | *Carduelis carduelis* | Alister Clunas |
| 4125 | | O.fringillina | | | | UNITED KINGDOM | ♀ | Ceredigion | 2 | Llechryd, Llys y Wern | 60 | 4.x.2021 | Goldcrest | *Regulus regulus* | Wendy James |
| 4145 | | O.fringillina | | | | UNITED KINGDOM | ♂ | Skye | 2 | Portree | 40 | 22.vi.2021 | House Sparrow | *Passer domesticus* | Jonathan Jones |
| 4221 | | O.fringillina | | | | UNITED KINGDOM | ♀ | Staffordshire | 3 | Kingswinford, Wall Heath, Holbeache Gardens | 82 | 9.vii.2021 | Dunnock | *Prunella modularis* | Samuel Bradley |
| 4241 | | O.fringillina | | | | UNITED KINGDOM | ♂ | Moray | 3 | Orton By Fochabers | 60 | 21.viii.2021 | Robin | *Erithacus rubecula* | Alastair Young |
| 4244 | | O.fringillina | | | | UNITED KINGDOM | ♀ | Moray |  | Orton By Fochabers | 60 | 16.viii.2021 | Robin | *Erithacus rubecula* | Alastair Young |
| 4246 | | O.fringillina | | | | UNITED KINGDOM |  | Moray | 3 | Orton By Fochabers | 60 | 4.ix.2021 | Siskin | *Spinus spinus* | Alistair Young |
| 4247 | | O.fringillina | | | | UNITED KINGDOM |  | Moray | 3 | Orton By Fochabers | 60 | 2.x.2021 | Chaffinch | *Fringilla coelebs* | Alistair Young |
| 4250 | | O.fringillina | | | | UNITED KINGDOM | ♀ | Moray | 2 | Orton By Fochabers | 60 | 13.ix.2021 | Treecreeper | *Certhia familiaris* | Alistair Young |
| 4251 | | O.fringillina | | | | UNITED KINGDOM |  | Moray | 3 | Orton By Fochabers | 60 |  | Goldfinch | *Carduelis carduelis* | Alistair Young |
| 4253 | | O.fringillina | | | | UNITED KINGDOM | ♀ | Moray | 3 | Orton By Fochabers | 60 | 10.viii.2021 | Robin | *Erithacus rubecula* | Alastair Young |
| 4260 | | O.fringillina | | | | UNITED KINGDOM |  | Moray | 3 | Orton By Fochabers | 60 | 6.xi.2021 | Goldfinch | *Carduelis carduelis* | Alistair Young |
| 4321 | | O.fringillina | | | | UNITED KINGDOM | ♂ | Surrey | 2 | Leith Hill, Duke's Warren | 250 | 11.viii.2021 | Goldcrest | *Regulus regulus* | Paul Stevenson |
| 4481 | | O.fringillina | | | | UNITED KINGDOM |  | Dorset |  | Hogchester | 64 | 3.viii.2021 | Robin | *Erithacus rubecula* | Susan Murphy |
| 4482 | | O.fringillina | | | | UNITED KINGDOM |  | Devon | 2 | Mutters Moor | 183 | 24.viii.2021 | Whitethroat | *Curruca communis* | Susan Murphy |
| 4484 | | O.fringillina | | | | UNITED KINGDOM | ♀ | Devon | 2 | Bicton Common,Pebblebed Heaths | 121 | 23.ix.2021 | Dartford Warbler | *Curruca undata* | Susan Murphy |
| 4485 | | O.fringillina | | | | UNITED KINGDOM | ♀ | Devon | 2 | Seaton Wetlands, Black Hole Marsh | 5 | 14.xii.2021 | Blue Tit | *Cyanistes caeruleus* | Susan Murphy |
| 4722 | | O.fringillina | | | | UNITED KINGDOM |  | Derbyshire | 3 | Creswell. Elmton WTW | 117 | 11.vii.2021 | Blackbird | *Turdus merula* | Eleanor Wilkins |
| 4724 | | O.fringillina | | | | UNITED KINGDOM | ♀ | Derbyshire |  | Worksop, Bondhay | 123 | 26.ix.2021 | Blackcap | *Sylvia atricapilla* | Eleanor Wilkins |
| 4781 | | O.fringillina | | | | UNITED KINGDOM | ♀ | Argyll | 2 | Audindarroch | 30 | 2.x.2021 | Great Tit | *Parus major* | Kirstie Ross |
| 4842 | | O.fringillina | | | | UNITED KINGDOM | ♀ | Powys |  | Talgarth, Llangssty | 160 | 25.viii.2021 | Willow Warbler | *Phylloscopus trochilus* | Llangorse Ringing Group |
| 4844 | | O.fringillina | | | | UNITED KINGDOM | ♂ | Powys | 3 | Talgarth, Llangssty | 160 | 11.ix.2021 | Treecreeper | *Certhia familiaris* | Llangorse Ringing Group |
| 4845 | | O.fringillina | | | | UNITED KINGDOM |  | Powys | 3 | Talgarth, Llangssty | 160 | 21.ix.2021 | Goldcrest | *Regulus regulus* | Llangorse Ringing Group |
| 4904 | | O.fringillina | | | | UNITED KINGDOM | ♀ | East Sussex | 2 | Icklesham | 10 | 13.viii.2021 | Garden Warbler | *Sylvia borin* | Rye Bay Ringing Group |
| 4908 | | O.fringillina | | | | UNITED KINGDOM |  | East Sussex | 2 | Icklesham | 10 | 18.viii.2021 | Reed Warbler | *Acrocephalus scirpaceus* | Rye Bay Ringing Group |
| 4912 | | O.fringillina | | | | UNITED KINGDOM | ♀ | East Sussex | 2 | Icklesham | 10 | 28.viii.2021 | Whitethroat | *Curruca communis* | Rye Bay Ringing Group |
| 4913 | | O.fringillina | | | | UNITED KINGDOM | ♀ | East Sussex | 2 | Icklesham | 10 | 1.ix.2021 | Whitethroat | *Curruca communis* | Rye Bay Ringing Group |
| 4914 | | O.fringillina | | | | UNITED KINGDOM | ♀ | East Sussex | 2 | Icklesham | 10 | 2.ix.2021 | Reed Warbler | *Acrocephalus scirpaceus* | Rye Bay Ringing Group |
| 4915 | | O.fringillina | | | | UNITED KINGDOM | ♀ | East Sussex | 2 | Icklesham | 10 | 9.ix.2021 |  |  | Rye Bay Ringing Group |
| 4916 | | O.fringillina | | | | UNITED KINGDOM | ♀ | East Sussex | 3 | Icklesham | 10 | 24.ix.2021 | Blackcap | *Sylvia atricapilla* | Rye Bay Ringing Group |
| 4917 | | O.fringillina | | | | UNITED KINGDOM | ♀ | East Sussex |  | Icklesham | 10 | 29.ix.2021 | Whitethroat | *Curruca communis* | Rye Bay Ringing Group |
| 4918 | | O.fringillina | | | | UNITED KINGDOM | ♀ | East Sussex | 3 | Icklesham | 53 | 14.x.2021 | Goldfinch | *Carduelis carduelis* | Rye Bay Ringing Group |
| 4919 | | O.fringillina | | | | UNITED KINGDOM | ♀ | East Sussex | 3 | Icklesham | 53 | 14.x.2021 | Goldfinch | *Carduelis carduelis* | Rye Bay Ringing Group |
| 4920 | | O.fringillina | | | | UNITED KINGDOM | ♂ | East Sussex | 2 | Icklesham | 53 | 15.x.2021 |  |  | Rye Bay Ringing Group |
| 4950 | | O.fringillina | | | | UNITED KINGDOM | ♀ | Rutland | 3 | Rutland Water Nature Reserve | 86 | 25.viii.2021 | Robin | *Erithacus rubecula* | Luke Nelson |
| 4951 | | O.fringillina | | | | UNITED KINGDOM | ♂ | Rutland | 3 | Rutland Water Nature Reserve | 86 | 25.viii.2021 |  |  | Luke Nelson |
| 5126 | | O.fringillina | | | | UNITED KINGDOM | ♀ | Suffolk | 3 | Brandon | 15 | 20.ix.2021 | Robin | *Erithacus rubecula* | Greg Conway |
| 5139 | | O.fringillina | | | | UNITED KINGDOM | ♂ | Suffolk | 2 | Brandon | 15 | 30.viii.2021 | Robin | *Erithacus rubecula* | Greg Conway |
| 5261 | | O.fringillina | | | | UNITED KINGDOM |  | Essex | 3 | Leigh-on-Sea, Two Tree Island | 0 | 4.vii.2021 | Whitethroat | *Curruca communis* | David Wilkinson |
| 5262 | | O.fringillina | | | | UNITED KINGDOM | ♀ | Essex | 3 | Leigh-on-Sea, Two Tree Island | 0 | 11.vii.2021 | Whitethroat | *Curruca communis* | David Wilkinson |
| 5263 | | O.fringillina | | | | UNITED KINGDOM | ♀ | Essex | 3 | Leigh-on-Sea, Two Tree Island | 0 | 26.vii.2021 | Blackcap | *Sylvia atricapilla* | David Wilkinson |
| 5264 | | O.fringillina | | | | UNITED KINGDOM |  | Essex | 3 | Leigh-on-Sea, Two Tree Island | 0 | 26.vii.2021 | Reed Warbler | *Acrocephalus scirpaceus* | David Wilkinson |
| 5265 | | O.fringillina | | | | UNITED KINGDOM | ♀ | Essex | 3 | Leigh-on-Sea, Two Tree Island | 0 | 26.vii.2021 | Reed Warbler | *Acrocephalus scirpaceus* | David Wilkinson |
| 5267 | | O.fringillina | | | | UNITED KINGDOM | ♀ | Essex | 3 | Chelmsford | 55 | 8.viii.2021 | Goldfinch | *Carduelis carduelis* | David Wilkinson |
| 5268 | | O.fringillina | | | | UNITED KINGDOM |  | Essex | 2 | Gunners Park and Shoebury Ranges Nature Reserve | 0 | 16.viii.2021 | Greenfinch | *Chloris chloris* | David Wilkinson |
| 5269 | | O.fringillina | | | | UNITED KINGDOM | ♀ | Essex |  | Gunners Park and Shoebury Ranges Nature Reserve | 0 | 16.viii.2021 | Whitethroat | *Curruca communis* | David Wilkinson |
| 5270 | | O.fringillina | | | | UNITED KINGDOM | ♀ | Essex | 3 | Leigh-on-Sea, Two Tree Island | 0 | 29.viii.2021 | Whitethroat | *Curruca communis* | David Wilkinson |
| 5315 | | O.fringillina | | | | UNITED KINGDOM |  | Norfolk | 3 | Thetford | 13 | 26.vii.2021 | Goldfinch | *Carduelis carduelis* | Joanne Lashwood |
| 5319 | | O.fringillina | | | | UNITED KINGDOM | ♀ | Norfolk | 3 | Thetford | 13 | 1.viii.2021 | Robin | *Erithacus rubecula* | Joanne Lashwood |
| 5536 | | O.fringillina | | | | ISLE OF MAN |  |  |  | Calf of Man | 80 | 31.vii.2021 | Goldfinch | *Carduelis carduelis* | Calf of Man Bird Observatory |
| 5590 | | O.fringillina | | | | UNITED KINGDOM |  | Carmarthenshire | 3 | Bancyffordd | 205 | 12.vii.2021 | Dunnock | *Prunella modularis* | A. Turner |
| 5600 | | O.fringillina | | | | UNITED KINGDOM | ♂ | Carmarthenshire | 2 | Bancyffordd | 205 | 24.vii.2021 | Robin | *Erithacus rubecula* | A. Turner |
| 5605 | | O.fringillina | | | | UNITED KINGDOM |  | Skye | 3 | Portree | 40 | 3.viii.2021 | House Sparrow | *Passer domesticus* | Jonathan Jones |
| 5606 | | O.fringillina | | | | UNITED KINGDOM | ♂ | Skye | 3 | Edinbane | 5 | 7.viii.2021 | Robin | *Erithacus rubecula* | Jonathan Jones |
| 5610 | | O.fringillina | | | | UNITED KINGDOM |  | Skye | 3 | Edinbane | 5 | 7.viii.2021 | Chaffinch | *Fringilla coelebs* | Jonathan Jones |
| 5611 | | O.fringillina | | | | UNITED KINGDOM |  | Skye |  | Edinbane | 5 | 7.viii.2021 |  |  | Jonathan Jones |
| 5612 | | O.fringillina | | | | UNITED KINGDOM | ♂ | Skye | 3 | Edinbane | 5 | 7.viii.2021 | Robin | *Erithacus rubecula* | Jonathan Jones |
| 5617 | | O.fringillina | | | | UNITED KINGDOM |  | Skye | 3 | Edinbane | 5 | 7.viii.2021 |  |  | Jonathan Jones |
| 5618 | | O.fringillina | | | | UNITED KINGDOM |  | Skye | 3 | Edinbane | 5 | 7.viii.2021 |  |  | Jonathan Jones |
| 5784 | | O.fringillina | | | | UNITED KINGDOM | ♀ | Cheshire | 3 | Woolston Eyes | 10 | 3.viii.2021 | Reed Warbler | *Acrocephalus scirpaceus* | Kieran Foster |
| 5785 | | O.fringillina | | | | UNITED KINGDOM |  | Cheshire | 2 | Woolston Eyes | 10 | 4.ix.2021 | Reed Warbler | *Acrocephalus scirpaceus* | Kieran Foster |
| 5787 | | O.fringillina | | | | UNITED KINGDOM |  | Cheshire | 3 | Woolston Eyes | 10 | 24.viii.2021 | Reed Warbler | *Acrocephalus scirpaceus* | Kieran Foster |
| 5793 | | O.fringillina | | | | UNITED KINGDOM | ♂ | Cheshire | 3 | Woolston Eyes | 10 | 22.viii.2021 | Whitethroat | *Curruca communis* | Kieran Foster |
| 5794 | | O.fringillina | | | | UNITED KINGDOM |  | Cheshire | 3 | Woolston Eyes | 10 | 19.viii.2021 | Robin | *Erithacus rubecula* | Kieran Foster |
| 5796 | | O.fringillina | | | | UNITED KINGDOM |  | Cheshire | 3 | Woolston Eyes | 10 | 14.viii.2021 | Reed Warbler | *Acrocephalus scirpaceus* | Kieran Foster |
| 5797 | | O.fringillina | | | | UNITED KINGDOM |  | Cheshire | 2 | Woolston Eyes | 10 | 19.viii.2021 | Reed Warbler | *Acrocephalus scirpaceus* | Kieran Foster |
| 5800 | | O.fringillina | | | | UNITED KINGDOM |  | Cheshire | 3 | Woolston Eyes | 10 | 19.viii.2021 | Reed Warbler | *Acrocephalus scirpaceus* | Kieran Foster |
| 5801 | | O.fringillina | | | | UNITED KINGDOM | ♀ | Vale of Glamorgan | 2 | Kenfig NNR | 5 | 21.viii.2021 | Reed Warbler | *Acrocephalus scirpaceus* | Kenfig RG |
| 5802 | | O.fringillina | | | | UNITED KINGDOM | ♀ | Vale of Glamorgan | 2 | Kenfig NNR | 5 | 3.xi.2021 | Cetti's Warbler | *Cettia cetti* | Kenfig RG |
| 5823 | | O.fringillina | | | | ISLE OF MAN | ♂ |  | 3 | Calf of Man | 80 | 12.ix.2021 | Willow Warbler | *Phylloscopus trochilus* | Calf of Man Bird Observatory |
| 5828 | | O.fringillina | | | | ISLE OF MAN |  |  | 3 | Calf of Man | 80 | 28.ix.2021 | Goldcrest | *Regulus regulus* | Calf of Man Bird Observatory |
| 5829 | | O.fringillina | | | | ISLE OF MAN |  |  | 3 | Calf of Man | 80 | 4.x.2021 | Goldcrest | *Regulus regulus* | Calf of Man Bird Observatory |
| 5830 | | O.fringillina | | | | ISLE OF MAN |  |  | damaged | Calf of Man | 80 | 10.x.2021 | Goldfinch | *Carduelis carduelis* | Calf of Man Bird Observatory |
| 5831 | | O.fringillina | | | | ISLE OF MAN |  |  | 3 | Calf of Man | 80 | 11.x.2021 | Greenfinch | *Chloris chloris* | Calf of Man Bird Observatory |
| 5832 | | O.fringillina | | | | ISLE OF MAN |  |  | 3 | Calf of Man | 80 | 13.x.2021 | Dunnock | *Prunella modularis* | Calf of Man Bird Observatory |
| 5861 | | O.fringillina | | | | UNITED KINGDOM | ♀ | Suffolk | 3 | Felixstowe, Levington, Levington Reed Bed | 5 | 18.viii.2021 | Reed Warbler | *Acrocephalus scirpaceus* | Anna Alam/Mick Wright |
| 5862 | | O.fringillina | | | | UNITED KINGDOM | ♀ | Suffolk | 3 | Felixstowe, Levington, Levington Reed Bed | 5 | 2.ix.2021 | Reed Warbler | *Acrocephalus scirpaceus* | Anna Alam/Mick Wright |
| 5902 | | O.fringillina | | | | UNITED KINGDOM | ♂ | Norfolk | 3 | Thetford | 13 | 29.vi.2021 | Blue Tit | *Cyanistes caeruleus* | Joanne Lashwood |
| 5946 | | O.fringillina | | | | UNITED KINGDOM | ♀ | Skye | 3 | Varagill | 60 | 24.viii.2021 |  |  | Jonathan Jones |
| 5947 | | O.fringillina | | | | UNITED KINGDOM | ♂ | Skye | 3 | Varagill | 60 | 28.viii.2021 | Goldcrest | *Regulus regulus* | Jonathan Jones |
| 5955 | | O.fringillina | | | | UNITED KINGDOM |  | Skye | 3 | Hungladder | 40 | 28.viii.2021 | Robin | *Erithacus rubecula* | Jonathan Jones |
| 5956 | | O.fringillina | | | | UNITED KINGDOM | ♀ | Skye | 3 | Hungladder | 40 | 28.viii.2021 |  |  | Jonathan Jones |
| 5958 | | O.fringillina | | | | UNITED KINGDOM | ♀ | Skye | 3 | Hungladder | 40 | 2.ix.2021 | Chaffinch | *Fringilla coelebs* | Jonathan Jones |
| 5981 | | O.fringillina | | | | UNITED KINGDOM | ♀ | Skye | 3 | Hungladder | 40 | 8.ix.2021 | Great Tit | *Parus major* | Jonathan Jones |
| 5985 | | O.fringillina | | | | UNITED KINGDOM | ♂ | Skye | 2 | Hungladder | 40 | 8.ix.2021 | Chaffinch | *Fringilla coelebs* | Jonathan Jones |
| 5987 | | O.fringillina | | | | UNITED KINGDOM |  | Skye | 3 | Hungladder | 40 | 12.ix.2021 | Robin | *Erithacus rubecula* | Jonathan Jones |
| 5991 | | O.fringillina | | | | UNITED KINGDOM |  | Skye | 3 | Varagill | 60 | 14.ix.2021 | Siskin | *Spinus spinus* | Jonathan Jones |
| 5992 | | O.fringillina | | | | UNITED KINGDOM | ♂ | Skye | 3 | Varagill | 60 | 14.ix.2021 | Goldcrest | *Regulus regulus* | Jonathan Jones |
| 5993 | | O.fringillina | | | | UNITED KINGDOM | ♀ | Skye | 3 | Varagill | 60 | 16.ix.2021 | Greenfinch | *Chloris chloris* | Jonathan Jones |
| 5994 | | O.fringillina | | | | UNITED KINGDOM | ♀ | Skye | 3 | Varagill | 60 | 18.ix.2021 | Treecreeper | *Certhia familiaris* | Jonathan Jones |
| 5995 | | O.fringillina | | | | UNITED KINGDOM |  | Skye | 3 | Portree | 40 | 1.x.2021 | Chaffinch | *Fringilla coelebs* | Jonathan Jones |
| 5996 | | O.fringillina | | | | UNITED KINGDOM |  | Skye | 3 | Portree | 40 | 2.x.2021 | Greenfinch | *Chloris chloris* | Jonathan Jones |
| 5997 | | O.fringillina | | | | UNITED KINGDOM |  | Skye | 3 | Edinbane, Skye | 5 | 5.x.2021 | Chaffinch | *Fringilla coelebs* | Jonathan Jones |
| 6049 | | O.fringillina | | | | ISLE OF MAN | ♂ |  | 3 | Calf of Man | 80 | 25.viii.2021 | Robin | *Erithacus rubecula* | Calf of Man Bird Observatory |
| 6051 | | O.fringillina | | | | ISLE OF MAN | ♂ |  | 2 | Calf of Man | 80 | 27.viii.2021 | Goldcrest | *Regulus regulus* | Calf of Man Bird Observatory |
| 6054 | | O.fringillina | | | | ISLE OF MAN | ♂ |  | 3 | Calf of Man | 80 | 29.viii.2021 | Willow Warbler | *Phylloscopus trochilus* | Calf of Man Bird Observatory |
| 6055 | | O.fringillina | | | | ISLE OF MAN |  |  | 2 | Calf of Man | 80 | 31.viii.2021 | Goldcrest | *Regulus regulus* | Calf of Man Bird Observatory |
| 6057 | | O.fringillina | | | | ISLE OF MAN |  |  | 3 | Calf of Man | 80 | 8.ix.2021 | Willow Warbler | *Phylloscopus trochilus* | Calf of Man Bird Observatory |
| 6059 | | O.fringillina | | | | ISLE OF MAN |  |  | 3 | Calf of Man | 80 | 10.ix.2021 | Goldfinch | *Carduelis carduelis* | Calf of Man Bird Observatory |
| 6060 | | O.fringillina | | | | ISLE OF MAN | ♀ |  | 3 | Calf of Man | 80 | 10.ix.2021 |  |  | Calf of Man Bird Observatory |
| 7001 | | O.fringillina | | | | UNITED KINGDOM | ♂ | Carmarthenshire | 3 | Bancyffordd | 205 | 26.vii.2021 | Robin | *Erithacus rubecula* | A. Turner |
| 7003 | | O.fringillina | | | | UNITED KINGDOM | ♂ | Carmarthenshire | 3 | Bancyffordd | 205 | 26.vii.2021 | Dunnock | *Prunella modularis* | A. Turner |
| 7004 | | O.fringillina | | | | UNITED KINGDOM |  | Carmarthenshire | 3 | Bancyffordd | 205 | 27.vii.2021 | House Sparrow | *Passer domesticus* | A. Turner |
| 7011 | | O.fringillina | | | | UNITED KINGDOM | ♀ | Carmarthenshire | 3 | Bancyffordd | 205 | 30.vii.2021 | Chaffinch | *Fringilla coelebs* | A. Turner |
| 7013 | | O.fringillina | | | | UNITED KINGDOM | ♀ | Carmarthenshire | 3 | Bancyffordd | 205 | 30.vii.2021 |  |  | A. Turner |
| 7017 | | O.fringillina | | | | UNITED KINGDOM |  | Carmarthenshire | 3 | Bancyffordd | 205 | 3.viii.2021 | Dunnock | *Prunella modularis* | A. Turner |
| 7018 | | O.fringillina | | | | UNITED KINGDOM | ♂ | Carmarthenshire | 3 | Bancyffordd | 205 | 3.viii.2021 | Chaffinch | *Fringilla coelebs* | A. Turner |
| 7021 | | O.fringillina | | | | UNITED KINGDOM |  | Carmarthenshire | 3 | Bancyffordd | 205 | 5.viii.2021 |  |  | A. Turner |
| 7022 | | O.fringillina | | | | UNITED KINGDOM | ♂ | Carmarthenshire | 3 | Bancyffordd | 205 | 6.viii.2021 | House Sparrow | *Passer domesticus* | A. Turner |
| 7026 | | O.fringillina | | | | UNITED KINGDOM | ♂ | Carmarthenshire |  | Bancyffordd | 205 | 7.viii.2021 | Blue Tit | *Cyanistes caeruleus* | A. Turner |
| 7027 | | O.fringillina | | | | UNITED KINGDOM | ♀ | Carmarthenshire |  | Bancyffordd | 205 | 8.viii.2021 | Dunnock | *Prunella modularis* | A. Turner |
| 7029 | | O.fringillina | | | | UNITED KINGDOM |  | Carmarthenshire |  | Bancyffordd | 205 | 10.viii.2021 | House Sparrow | *Passer domesticus* | A. Turner |
| 7035 | | O.fringillina | | | | UNITED KINGDOM | ♂ | Carmarthenshire |  | Bancyffordd | 205 | 15.viii.2021 | Chaffinch | *Fringilla coelebs* | A. Turner |
| 7036 | | O.fringillina | | | | UNITED KINGDOM | ♂ | Carmarthenshire | 3 | Bancyffordd | 205 | 15.viii.2021 | Chaffinch | *Fringilla coelebs* | A. Turner |
| 7037 | | O.fringillina | | | | UNITED KINGDOM | ♀ | Carmarthenshire | 3 | Bancyffordd | 205 | 15.viii.2021 | House Sparrow | *Passer domesticus* | A. Turner |
| 7038 | | O.fringillina | | | | UNITED KINGDOM |  | Carmarthenshire | 2 | Bancyffordd | 205 | 15.viii.2021 | Dunnock | *Prunella modularis* | A. Turner |
| 7161 | | O.fringillina | | | | UNITED KINGDOM |  | Inverness-shire | 3 | Inverness, Drummond | 34 | 23.vii.2021 | Chaffinch | *Fringilla coelebs* | Hugh Insley |
| 7162 | | O.fringillina | | | | UNITED KINGDOM | ♀ | Inverness-shire | 3 | Inverness, Drummond | 34 | 23.vii.2021 | Chaffinch | *Fringilla coelebs* | Hugh Insley |
| 7164 | | O.fringillina | | | | UNITED KINGDOM |  | Inverness-shire | 3 | Carse of Ardersier | 5 | 24.vii.2021 | Wren | *Troglodytes troglodytes* | Hugh Insley |
| 7165 | | O.fringillina | | | | UNITED KINGDOM |  | Inverness-shire | 3 | Carse of Ardersier | 5 | 29.vii.2021 | Willow Warbler | *Phylloscopus trochilus* | Hugh Insley |
| 7167 | | O.fringillina | | | | UNITED KINGDOM |  | Inverness-shire | 3 | Carse of Ardersier | 5 | 29.vii.2021 | Willow Warbler | *Phylloscopus trochilus* | Hugh Insley |
| 7168 | | O.fringillina | | | | UNITED KINGDOM | ♂ | Inverness-shire | 3 | Carse of Ardersier | 5 | 26.vii.2021 | Willow Warbler | *Phylloscopus trochilus* | Hugh Insley |
| 7169 | | O.fringillina | | | | UNITED KINGDOM | ♂ | Inverness-shire | 3 | Carse of Ardersier | 5 | 29.vii.2021 | Willow Warbler | *Phylloscopus trochilus* | Hugh Insley |
| 7175 | | O.fringillina | | | | UNITED KINGDOM | ♂ | Inverness-shire | 3 | Carse of Ardersier | 5 | 1.viii.2021 | Willow Warbler | *Phylloscopus trochilus* | Hugh Insley |
| 7177 | | O.fringillina | | | | UNITED KINGDOM | ♂ | Inverness-shire | 2&3 | Carse of Ardersier | 5 | 30.vii.2021 | Willow Warbler | *Phylloscopus trochilus* | Hugh Insley |
| 7241 | | O.fringillina | | | | UNITED KINGDOM | ♀ | Inverness-shire | 3 | Carse of Ardersier | 5 | 1.viii.2021 | Willow Warbler | *Phylloscopus trochilus* | Hugh Insley |
| 7243 | | O.fringillina | | | | UNITED KINGDOM | ♂ | Inverness-shire |  | Carse of Ardersier | 5 | 1.viii.2021 | House Sparrow | *Passer domesticus* | Hugh Insley |
| 7246 | | O.fringillina | | | | UNITED KINGDOM | ♂ | Inverness-shire | 3 | Carse of Ardersier | 5 | 2.viii.2021 | Blackbird | *Turdus merula* | Hugh Insley |
| 7247 | | O.fringillina | | | | UNITED KINGDOM | ♂ | Inverness-shire | 3 | Carse of Ardersier | 5 | 3.viii.2021 | Willow Warbler | *Phylloscopus trochilus* | Hugh Insley |
| 7248 | | O.fringillina | | | | UNITED KINGDOM | ♂ | Inverness-shire |  | Carse of Ardersier | 5 | 5.viii.2021 | Robin | *Erithacus rubecula* | Hugh Insley |
| 7251 | | O.fringillina | | | | UNITED KINGDOM |  | Inverness-shire | 3 | Carse of Ardersier | 5 | 8.viii.2021 | Willow Warbler | *Phylloscopus trochilus* | Hugh Insley |
| 7252 | | O.fringillina | | | | UNITED KINGDOM |  | Inverness-shire | 3 | Carse of Ardersier | 5 | 9.viii.2021 | Willow Warbler | *Phylloscopus trochilus* | Hugh Insley |
| 7253 | | O.fringillina | | | | UNITED KINGDOM | ♂ | Inverness-shire | 3 | Carse of Ardersier | 5 | 9.viii.2021 | House Sparrow | *Passer domesticus* | Hugh Insley |
| 7255 | | O.fringillina | | | | UNITED KINGDOM | ♀ | Inverness-shire | 3 | Carse of Ardersier | 5 | 11.viii.2021 | Robin | *Erithacus rubecula* | Hugh Insley |
| 7256 | | O.fringillina | | | | UNITED KINGDOM | ♀ | Inverness-shire | 3 | Carse of Ardersier | 5 | 11.viii.2021 | Robin | *Erithacus rubecula* | Hugh Insley |
| 7257 | | O.fringillina | | | | UNITED KINGDOM | ♀ | Inverness-shire | 3 | Carse of Ardersier | 5 | 11.viii.2021 | Willow Warbler | *Phylloscopus trochilus* | Hugh Insley |
| 7258 | | O.fringillina | | | | UNITED KINGDOM | ♀ | Inverness-shire | 3 | Carse of Ardersier | 5 | 11.viii.2021 | Willow Warbler | *Phylloscopus trochilus* | Hugh Insley |
| 7259 | | O.fringillina | | | | UNITED KINGDOM | ♀ | Inverness-shire | 3 | Carse of Ardersier | 5 | 11.viii.2021 | Robin | *Erithacus rubecula* | Hugh Insley |
| 7261 | | O.fringillina | | | | UNITED KINGDOM | ♂ | Inverness-shire |  | Carse of Ardersier | 5 | 13.viii.2021 | Willow Warbler | *Phylloscopus trochilus* | Hugh Insley |
| 7262 | | O.fringillina | | | | UNITED KINGDOM | ♀ | Inverness-shire | 3 | Carse of Ardersier | 5 | 13.viii.2021 | Blackcap | *Sylvia atricapilla* | Hugh Insley |
| 7263 | | O.fringillina | | | | UNITED KINGDOM | ♀ | Inverness-shire | 3 | Carse of Ardersier | 5 | 13.viii.2021 | Willow Warbler | *Phylloscopus trochilus* | Hugh Insley |
| 7264 | | O.fringillina | | | | UNITED KINGDOM | ♂ | Inverness-shire |  | Carse of Ardersier | 5 | 13.viii.2021 | Willow Warbler | *Phylloscopus trochilus* | Hugh Insley |
| 7266 | | O.fringillina | | | | UNITED KINGDOM |  | Inverness-shire | 3 | Carse of Ardersier | 5 | 15.viii.2021 | Willow Warbler | *Phylloscopus trochilus* | Hugh Insley |
| 7267 | | O.fringillina | | | | UNITED KINGDOM |  | Inverness-shire | 3 | Carse of Ardersier | 5 | 15.viii.2021 | Willow Warbler | *Phylloscopus trochilus* | Hugh Insley |
| 7269 | | O.fringillina | | | | UNITED KINGDOM | ♀ | Inverness-shire | 3 | Carse of Ardersier | 5 | 15.viii.2021 | Robin | *Erithacus rubecula* | Hugh Insley |
| 7270 | | O.fringillina | | | | UNITED KINGDOM | ♀ | Inverness-shire | 3 | Carse of Ardersier | 5 | 17.viii.2021 | Robin | *Erithacus rubecula* | Hugh Insley |
| 7271 | | O.fringillina | | | | UNITED KINGDOM |  | Inverness-shire | 3 | Carse of Ardersier | 5 | 19.viii.2021 | Willow Warbler | *Phylloscopus trochilus* | Hugh Insley |
| 7272 | | O.fringillina | | | | UNITED KINGDOM | ♂ | Inverness-shire | 3 | Carse of Ardersier | 5 | 19.viii.2021 | Willow Warbler | *Phylloscopus trochilus* | Hugh Insley |
| 7273 | | O.fringillina | | | | UNITED KINGDOM | ♀ | Inverness-shire | 3 | Carse of Ardersier | 5 | 19.viii.2021 | Willow Warbler | *Phylloscopus trochilus* | Hugh Insley |
| 7274 | | O.fringillina | | | | UNITED KINGDOM | ♀ | Inverness-shire | 3 | Carse of Ardersier | 5 | 20.viii.2021 | Willow Warbler | *Phylloscopus trochilus* | Hugh Insley |
| 7275 | | O.fringillina | | | | UNITED KINGDOM | ♀ | Inverness-shire | 3 | Carse of Ardersier | 5 | 20.viii.2021 | Willow Warbler | *Phylloscopus trochilus* | Hugh Insley |
| 7276 | | O.fringillina | | | | UNITED KINGDOM | ♂ | Inverness-shire | 3 | Carse of Ardersier | 5 | 20.viii.2021 | Willow Warbler | *Phylloscopus trochilus* | Hugh Insley |
| 7277 | | O.fringillina | | | | UNITED KINGDOM |  | Inverness-shire | 3 | Carse of Ardersier | 5 | 24.viii.2021 | Bullfinch | *Pyrrhula pyrrhula* | Hugh Insley |
| 7278 | | O.fringillina | | | | UNITED KINGDOM |  | Inverness-shire | 3 | Carse of Ardersier | 5 | 25.viii.2021 | Willow Warbler | *Phylloscopus trochilus* | Hugh Insley |
| 7279 | | O.fringillina | | | | UNITED KINGDOM |  | Inverness-shire | 3 | Carse of Ardersier | 5 | 27.viii.2021 | Blackbird | *Turdus merula* | Hugh Insley |
| 7280 | | O.fringillina | | | | UNITED KINGDOM |  | Inverness-shire | 3 | Carse of Ardersier | 5 | 27.viii.2021 | Willow Warbler | *Phylloscopus trochilus* | Hugh Insley |
| 7281 | | O.fringillina | | | | UNITED KINGDOM | ♂ | Devon | 3 | Ilfracombe, West Down, Buttercombe Barton | 160 | 2.viii.2021 | Greenfinch | *Chloris chloris* | Chris Dee |
| 7287 | | O.fringillina | | | | UNITED KINGDOM | ♂ | Devon | 3 | Ilfracombe, West Down, Buttercombe Barton | 160 | 9.viii.2021 | Blackcap | *Sylvia atricapilla* | Chris Dee |
| 7289 | | O.fringillina | | | | UNITED KINGDOM |  | Devon | 3 | Ilfracombe, West Down, Buttercombe Barton | 160 | 9.viii.2021 | Goldfinch | *Carduelis carduelis* | Chris Dee |
| 7290 | | O.fringillina | | | | UNITED KINGDOM |  | Devon | 3 | Ilfracombe, West Down, Buttercombe Barton | 160 | 9.viii.2021 | Greenfinch | *Chloris chloris* | Chris Dee |
| 7291 | | O.fringillina | | | | UNITED KINGDOM |  | Devon | 3 | Ilfracombe, West Down, Buttercombe Barton | 160 | 9.viii.2021 |  |  | Chris Dee |
| 7292 | | O.fringillina | | | | UNITED KINGDOM |  | Devon | 3 | Ilfracombe, West Down, Buttercombe Barton | 160 | 9.viii.2021 |  |  | Chris Dee |
| 7293 | | O.fringillina | | | | UNITED KINGDOM | ♀ | Devon | 3 | Ilfracombe, West Down, Buttercombe Barton | 160 | 9.viii.2021 |  |  | Chris Dee |
| 7294 | | O.fringillina | | | | UNITED KINGDOM | ♀ | Devon |  | Ilfracombe, West Down, Buttercombe Barton | 160 | 9.viii.2021 |  |  | Chris Dee |
| 7295 | | O.fringillina | | | | UNITED KINGDOM | ♂ | Devon | 3 | Ilfracombe, West Down, Buttercombe Barton | 160 | 9.viii.2021 |  |  | Chris Dee |
| 7297 | | O.fringillina | | | | UNITED KINGDOM | ♂ | Devon | 3 | Ilfracombe, West Down, Buttercombe Barton | 160 | 14.viii.2021 |  |  | Chris Dee |
| 7298 | | O.fringillina | | | | UNITED KINGDOM |  | Devon | 3 | Ilfracombe, West Down, Buttercombe Barton | 160 | 15.viii.2021 | Greenfinch | *Chloris chloris* | Chris Dee |
| 7299 | | O.fringillina | | | | UNITED KINGDOM |  | Devon | 3 | Ilfracombe, West Down, Buttercombe Barton | 160 | 25.viii.2021 | Chaffinch | *Fringilla coelebs* | Chris Dee |
| 7300 | | O.fringillina | | | | UNITED KINGDOM |  | Devon | 3 | Ilfracombe, West Down, Buttercombe Barton | 160 | 28.viii.2021 | Robin | *Erithacus rubecula* | Chris Dee |
| 7303 | | O.fringillina | | | | UNITED KINGDOM | ♂ | Carmarthenshire | 3 | Bancyffordd | 205 | 17.viii.2021 | House Sparrow | *Passer domesticus* | A. Turner |
| 7304 | | O.fringillina | | | | UNITED KINGDOM | ♀ | Carmarthenshire | 3 | Bancyffordd | 205 | 17.viii.2021 |  |  | A. Turner |
| 7305 | | O.fringillina | | | | UNITED KINGDOM | ♂ | Carmarthenshire |  | Bancyffordd | 205 | 17.viii.2021 |  |  | A. Turner |
| 7306 | | O.fringillina | | | | UNITED KINGDOM |  | Carmarthenshire | 3 | Bancyffordd | 205 | 17.viii.2021 | Robin | *Erithacus rubecula* | A. Turner |
| 7307 | | O.fringillina | | | | UNITED KINGDOM |  | Carmarthenshire | 3 | Bancyffordd | 205 | 18.viii.2021 | House Sparrow | *Passer domesticus* | A. Turner |
| 7308 | | O.fringillina | | | | UNITED KINGDOM |  | Carmarthenshire | 3 | Bancyffordd | 205 | 20.viii.2021 | Chaffinch | *Fringilla coelebs* | A. Turner |
| 7312 | | O.fringillina | | | | UNITED KINGDOM | ♂ | Carmarthenshire | 3 | Bancyffordd | 205 | 22.viii.2021 |  |  | A. Turner |
| 7313 | | O.fringillina | | | | UNITED KINGDOM |  | Carmarthenshire | 3 | Bancyffordd | 205 | 29.viii.2021 | Dunnock | *Prunella modularis* | A. Turner |
| 7314 | | O.fringillina | | | | UNITED KINGDOM |  | Carmarthenshire | 3 | Bancyffordd | 205 | 29.viii.2021 |  |  | A. Turner |
| 7315 | | O.fringillina | | | | UNITED KINGDOM |  | Carmarthenshire | 3 | Bancyffordd | 205 | 29.viii.2021 | Dunnock | *Prunella modularis* | A. Turner |
| 7316 | | O.fringillina | | | | UNITED KINGDOM | ♀ | Carmarthenshire | 3 | Bancyffordd | 205 | 31.viii.2021 | Wren | *Troglodytes troglodytes* | A. Turner |
| 7318 | | O.fringillina | | | | UNITED KINGDOM |  | Carmarthenshire | 3 | Bancyffordd | 205 | 14.ix.2021 | Dunnock | *Prunella modularis* | A. Turner |
| 7319 | | O.fringillina | | | | UNITED KINGDOM |  | Carmarthenshire | 3 | Bancyffordd | 205 | 14.ix.2021 | Dunnock | *Prunella modularis* | A. Turner |
| 7320 | | O.fringillina | | | | UNITED KINGDOM | ♀ | Carmarthenshire | 3 | Bancyffordd | 205 | 18.ix.2021 | House Sparrow | *Passer domesticus* | A. Turner |
| 7321 | | O.fringillina | | | | UNITED KINGDOM | ♂ | Devon | 2 | Ilfracombe, West Down, Buttercombe Barton | 160 | 28.viii.2021 | Greenfinch | *Chloris chloris* | Chris Dee |
| 7323 | | O.fringillina | | | | UNITED KINGDOM | ♂ | Devon | 2 | Ilfracombe, West Down, Buttercombe Barton | 160 | 28.viii.2021 |  |  | Chris Dee |
| 7324 | | O.fringillina | | | | UNITED KINGDOM | ♀ | Devon | 3 | Ilfracombe, West Down, Buttercombe Barton | 160 | 28.viii.2021 | Greenfinch | *Chloris chloris* | Chris Dee |
| 7325 | | O.fringillina | | | | UNITED KINGDOM | ♂ | Devon | 3 | Ilfracombe, West Down, Buttercombe Barton | 160 | 29.viii.2021 | Robin | *Erithacus rubecula* | Chris Dee |
| 7326 | | O.fringillina | | | | UNITED KINGDOM | ♀ | Devon | 2&3 | Ilfracombe, West Down, Buttercombe Barton | 160 | 29.viii.2021 |  |  | Chris Dee |
| 7327 | | O.fringillina | | | | UNITED KINGDOM | ♀ | Devon | 3 | Ilfracombe, West Down, Buttercombe Barton | 160 | 3.ix.2021 | Chaffinch | *Fringilla coelebs* | Chris Dee |
| 7328 | | O.fringillina | | | | UNITED KINGDOM | ♀ | Devon | 3 | Ilfracombe, West Down, Buttercombe Barton | 160 | 3.ix.2021 | Goldfinch | *Carduelis carduelis* | Chris Dee |
| 7329 | | O.fringillina | | | | UNITED KINGDOM |  | Devon | 3 | Ilfracombe, West Down, Buttercombe Barton | 160 | 3.ix.2021 |  |  | Chris Dee |
| 7381 | | O.fringillina | | | | UNITED KINGDOM | ♂ | Carmarthenshire | 2 | Bancyffordd | 205 | 18.ix.2021 | Great Tit | *Parus major* | A. Turner |
| 7382 | | O.fringillina | | | | UNITED KINGDOM | ♀ | Carmarthenshire | 2 | Bancyffordd | 205 | 21.ix.2021 | Chaffinch | *Fringilla coelebs* | A. Turner |
| 7383 | | O.fringillina | | | | UNITED KINGDOM | ♀ | Carmarthenshire | 2 | Bancyffordd | 205 | 22.ix.2021 | Robin | *Erithacus rubecula* | A. Turner |
| 7384 | | O.fringillina | | | | UNITED KINGDOM | ♀ | Carmarthenshire | 2 | Bancyffordd | 205 | 23.ix.2021 | Dunnock | *Prunella modularis* | A. Turner |
| 7443 | | O.fringillina | | | | UNITED KINGDOM | ♀ | Warwickshire | 3 | Priors Marston | 143 | 28.vii.2021 | Dunnock | *Prunella modularis* | Northants RG |
| 7449 | | O.fringillina | | | | UNITED KINGDOM |  | Northamptonshire | 2 | Northhampton, Harrington Airfield | 161 | 2.viii.2021 | Willow Warbler | *Phylloscopus trochilus* | Northants RG |
| 7745 | | O.fringillina | | | | UNITED KINGDOM | ♀ | Inverness-shire | 3 | Carse of Ardersier | 5 | 17.vii.2022 | Willow Warbler | *Phylloscopus trochilus* | Hugh Insley |
| 7747 | | O.fringillina | | | | UNITED KINGDOM | ♀ | Inverness-shire | 3 | Carse of Ardersier | 5 | 23.vii.2022 | Willow Warbler | *Phylloscopus trochilus* | Hugh Insley |
| 7748 | | O.fringillina | | | | UNITED KINGDOM | ♀ | Inverness-shire | 3 | Carse of Ardersier | 5 | 23.vii.2022 | Willow Warbler | *Phylloscopus trochilus* | Hugh Insley |
| 7750 | | O.fringillina | | | | UNITED KINGDOM | ♀ | Inverness-shire | 2 | Carse of Ardersier | 5 | 22.vii.2022 | Dunnock | *Prunella modularis* | Hugh Insley |
| 7752 | | O.fringillina | | | | UNITED KINGDOM | ♂ | Inverness-shire | 3 | Carse of Ardersier | 5 | 22.vii.2022 | Willow Warbler | *Phylloscopus trochilus* | Hugh Insley |
| 7753 | | O.fringillina | | | | UNITED KINGDOM | ♂ | Inverness-shire | 3 | Carse of Ardersier | 5 | 17.vii.2022 | Willow Warbler | *Phylloscopus trochilus* | Hugh Insley |
| 7755 | | O.fringillina | | | | UNITED KINGDOM | ♂ | Inverness-shire | 2 | Carse of Ardersier | 5 | 17.vii.2022 | Goldcrest | *Regulus regulus* | Hugh Insley |
| 7758 | | O.fringillina | | | | UNITED KINGDOM | ♂ | Inverness-shire | 3 | Carse of Ardersier | 5 | 23.vii.2022 | Willow Warbler | *Phylloscopus trochilus* | Hugh Insley |
| 7759 | | O.fringillina | | | | UNITED KINGDOM | ♂ | Inverness-shire | 3 | Carse of Ardersier | 5 | 23.vii.2022 | Coal Tit | *Periparus ater* | Hugh Insley |
| 7961 | | O.fringillina | | | | UNITED KINGDOM | ♀ | Inverness-shire | 3 | Carse of Ardersier | 5 | 30.viii.2021 | Willow Warbler | *Phylloscopus trochilus* | Hugh Insley |
| 7963 | | O.fringillina | | | | UNITED KINGDOM | ♀ | Inverness-shire | 3 | Carse of Ardersier | 5 | 3.ix.2021 | Blackcap | *Sylvia atricapilla* | Hugh Insley |
| 7964 | | O.fringillina | | | | UNITED KINGDOM | ♀ | Inverness-shire | 3 | Carse of Ardersier | 5 | 4.ix.2021 | Dunnock | *Prunella modularis* | Hugh Insley |
| 7965 | | O.fringillina | | | | UNITED KINGDOM | ♀ | Inverness-shire | 3 | Carse of Ardersier | 5 | 4.ix.2021 | Blackbird | *Turdus merula* | Hugh Insley |
| 7967 | | O.fringillina | | | | UNITED KINGDOM | ♀ | Inverness-shire | 3 | Carse of Ardersier | 5 | 8.ix.2021 | Blackcap | *Sylvia atricapilla* | Hugh Insley |
| 7969 | | O.fringillina | | | | UNITED KINGDOM |  | Inverness-shire | 3 | Carse of Ardersier | 5 | 8.ix.2021 | Chaffinch | *Fringilla coelebs* | Hugh Insley |
| 7972 | | O.fringillina | | | | UNITED KINGDOM |  | Inverness-shire | 3 | Carse of Ardersier | 5 | 20.ix.2021 | Dunnock | *Prunella modularis* | Hugh Insley |
| 7973 | | O.fringillina | | | | UNITED KINGDOM | ♂ | Inverness-shire | 3 | Carse of Ardersier | 5 | 25.ix.2021 | Robin | *Erithacus rubecula* | Hugh Insley |
| 7974 | | O.fringillina | | | | UNITED KINGDOM |  | Inverness-shire | 3 | Carse of Ardersier | 5 | 17.x.2021 | Goldcrest | *Regulus regulus* | Hugh Insley |
| 7977 | | O.fringillina | | | | UNITED KINGDOM | ♀ | Inverness-shire | 3 | Carse of Ardersier | 5 | 12.ix.2021 | Meadow Pipit | *Anthus pratensis* | Hugh Insley |
| 123A | | O.fringillina | | | | UNITED KINGDOM |  | Cumbria | 2 | Kirkbride | 12 | 8.vii.2020 | House Sparrow | *Passer domesticus* | Watchtree RG |
| 123B | | O.fringillina | | | | UNITED KINGDOM | ♂ | Cumbria | 2 | Kirkbride | 12 | 8.vii.2020 | House Sparrow | *Passer domesticus* | Watchtree RG |
| 172A | | O.fringillina | | | | UNITED KINGDOM | ♂ | County Durham | 3 | Butterknowle | 208 | 24.vii.2020 | Robin | *Erithacus rubecula* | John Black |
| 172B | | O.fringillina | | | | UNITED KINGDOM | ♂ | County Durham |  | Butterknowle | 208 | 24.vii.2020 | Robin | *Erithacus rubecula* | John Black |
| 2995A | | O.fringillina | | | | UNITED KINGDOM | ♀ | Norfolk | 3 | Walsey Hill | 8 | 14.ix.2021 | Reed Warbler | *Acrocephalus scirpaceus* | Norfolk Ornithologists Association |
| 5123A | | O.fringillina | | | | UNITED KINGDOM |  | Suffolk | 3 | Brandon | 15 | 20.viii.2021 | Robin | *Erithacus rubecula* | Greg Conway |
| 5123B | | O.fringillina | | | | UNITED KINGDOM |  | Suffolk | 3 | Brandon | 15 | 20.viii.2021 | Robin | *Erithacus rubecula* | Greg Conway |
| 5123C | | O.fringillina | | | | UNITED KINGDOM | ♀ | Suffolk | 3 | Brandon | 15 | 20.viii.2021 | Robin | *Erithacus rubecula* | Greg Conway |
| 5323B | | O.fringillina | | | | ISLE OF MAN | ♂ |  | 3 | Calf of Man | 80 | 4.vii.2021 |  |  | Calf of Man Bird Observatory |
| 5528A | | O.fringillina | | | | ISLE OF MAN | ♂ |  | 2 | Calf of Man | 80 | 22.vii.2021 | Meadow Pipit | *Anthus pratensis* | Calf of Man Bird Observatory |
| 5531A | | O.fringillina | | | | ISLE OF MAN | ♀ |  | 3 | Calf of Man | 80 | 24.vii.2021 |  |  | Calf of Man Bird Observatory |
| 5531B | | O.fringillina | | | | ISLE OF MAN | ♀ |  | 3 | Calf of Man | 80 | 24.vii.2021 |  |  | Calf of Man Bird Observatory |
| 5833A | | O.fringillina | | | | ISLE OF MAN | ♀ |  | 3 | Calf of Man | 80 | 15.x.2021 |  |  | Calf of Man Bird Observatory |
| 5833B | | O.fringillina | | | | ISLE OF MAN | ♀ |  | 3 | Calf of Man | 80 | 15.x.2021 |  |  | Calf of Man Bird Observatory |
| 7007A | | O.fringillina | | | | UNITED KINGDOM |  | Carmarthenshire | 2 | Bancyffordd | 205 | 27.vii.2021 | Robin | *Erithacus rubecula* | A. Turner |
| 7007B | | O.fringillina | | | | UNITED KINGDOM |  | Carmarthenshire | 3 | Bancyffordd | 205 | 27.vii.2021 | Robin | *Erithacus rubecula* | A. Turner |
| 7007C | | O.fringillina | | | | UNITED KINGDOM | ♂ | Carmarthenshire | 3 | Bancyffordd | 205 | 27.vii.2021 | Robin | *Erithacus rubecula* | A. Turner |
| 7023A | | O.fringillina | | | | UNITED KINGDOM |  | Carmarthenshire | 3 | Bancyffordd | 205 | 6.viii.2021 | House Sparrow | *Passer domesticus* | A. Turner |
| 7023B | | O.fringillina | | | | UNITED KINGDOM |  | Carmarthenshire | 3 | Bancyffordd | 205 | 6.viii.2021 | House Sparrow | *Passer domesticus* | A. Turner |
| 7039A | | O.fringillina | | | | UNITED KINGDOM |  | Carmarthenshire | 3 | Bancyffordd | 205 | 15.viii.2021 | House Sparrow | *Passer domesticus* | A. Turner |
| 7039B | | O.fringillina | | | | UNITED KINGDOM |  | Carmarthenshire | 3 | Bancyffordd | 205 | 15.viii.2021 | House Sparrow | *Passer domesticus* | A. Turner |
| 7040A | | O.fringillina | | | | UNITED KINGDOM | ♂ | Carmarthenshire | 3 | Bancyffordd | 205 | 15.viii.2021 | House Sparrow | *Passer domesticus* | A. Turner |
| 7040B | | O.fringillina | | | | UNITED KINGDOM | ♀ | Carmarthenshire | 3 | Bancyffordd | 205 | 15.viii.2021 | House Sparrow | *Passer domesticus* | A. Turner |
| 7254A | | O.fringillina | | | | UNITED KINGDOM |  | Inverness-shire | 3 | Carse of Ardersier | 5 | 11.viii.2021 | Robin | *Erithacus rubecula* | Hugh Insley |
| 7254B | | O.fringillina | | | | UNITED KINGDOM |  | Inverness-shire | 3 | Carse of Ardersier | 5 | 11.viii.2021 | Robin | *Erithacus rubecula* | Hugh Insley |
| 7254C | | O.fringillina | | | | UNITED KINGDOM |  | Inverness-shire | 3 | Carse of Ardersier | 5 | 11.viii.2021 | Robin | *Erithacus rubecula* | Hugh Insley |
| 7260B- small | | O.fringillina | | | | UNITED KINGDOM | ♂ | Inverness-shire | 3 | Carse of Ardersier | 5 | 12.viii.2021 | Willow Warbler | *Phylloscopus trochilus* | Hugh Insley |
| 7317A | | O.fringillina | | | | UNITED KINGDOM | ♀ | Carmarthenshire | 3 | Bancyffordd | 205 | 1.ix.2021 | Dunnock | *Prunella modularis* | A. Turner |
| 7317B | | O.fringillina | | | | UNITED KINGDOM |  | Carmarthenshire | 3 | Bancyffordd | 205 | 1.ix.2021 | Dunnock | *Prunella modularis* | A. Turner |
| 7317C | | O.fringillina | | | | UNITED KINGDOM |  | Carmarthenshire | 3 | Bancyffordd | 205 | 1.ix.2021 | Dunnock | *Prunella modularis* | A. Turner |
| A74 | | O.fringillina | | | | UNITED KINGDOM | ♀ | Northamptonshire | 3 | Northampton, Harrington Airfield | 150 | 7.ix.2020 | Yellowhammer | *Emberiza citrinella* | Helen Franklin |
| C23 | | O.fringillina | | | | UNITED KINGDOM |  | Northamptonshire | 3 | Greens Norton | 95 | 27.viii.2020 | Chiffchaff | *Phylloscopus collybita* | Chris Payne |
| D03 | | O.fringillina | | | | UNITED KINGDOM | ♀ | East Riding of Yorkshire | 2 | Flamborough | 45 | 29.ix.2020 | Yellowhammer | *Emberiza citrinella* | Andy Hood |
| E06 | | O.fringillina | | | | UNITED KINGDOM |  | Cumbria | 2 | Kirkbride | 14 | 20.viii.2021 | Treecreeper | *Certhia familiaris* | Watchtree RG |
| E12 | | O.fringillina | | | | UNITED KINGDOM |  | Cheshire | 3 | Mollington | 33 | 16.ix.2020 | Great Tit | *Parus major* | Phil Woollen |
| E14 | | O.fringillina | | | | UNITED KINGDOM |  | Cheshire | 3 | Mollington | 33 | 18.ix.2020 | Goldfinch | *Carduelis carduelis* | Phil Woollen |
| FF101 | | O.fringillina | | | | UNITED KINGDOM | ♀ | Somerset | 3 | Minehead, Bratton | 64 | 23.vii.2021 | Robin | *Erithacus rubecula* | Denise Wawman |
| FF102 | | O.fringillina | | | | UNITED KINGDOM | ♂ | Somerset | 3 | Minehead, Bratton | 64 | 23.vii.2021 | Robin | *Erithacus rubecula* | Denise Wawman |
| FF105 | | O.fringillina | | | | UNITED KINGDOM |  | Somerset | 2 | Minehead, Bratton | 64 | 25.vii.2021 | Blue Tit | *Cyanistes caeruleus* | Denise Wawman |
| FF108 | | O.fringillina | | | | UNITED KINGDOM | ♂ | Somerset | 3 | Minehead, Bratton | 64 | 2.viii.2021 | Dunnock | *Prunella modularis* | Denise Wawman |
| FF112 | | O.fringillina | | | | UNITED KINGDOM | ♀ | Somerset | 3 | Minehead, Bratton | 64 | 2.viii.2021 |  |  | Denise Wawman |
| FF113 | | O.fringillina | | | | UNITED KINGDOM | ♂ | Somerset | 2 | Minehead, Bratton | 64 | 2.viii.2021 |  |  | Denise Wawman |
| FF114 | | O.fringillina | | | | UNITED KINGDOM |  | Somerset | 3 | Minehead, Bratton | 64 | 2.viii.2021 | Robin | *Erithacus rubecula* | Denise Wawman |
| FF118 | | O.fringillina | | | | UNITED KINGDOM | ♂ | Somerset | 3 | Minehead, Bratton | 64 | 2.viii.2021 |  |  | Denise Wawman |
| FF119 | | O.fringillina | | | | UNITED KINGDOM | ♂ | Somerset | 3 | Minehead, Bratton | 64 | 2.viii.2021 |  |  | Denise Wawman |
| FF120 | | O.fringillina | | | | UNITED KINGDOM |  | Somerset | 3 | Minehead, Bratton | 64 | 2.viii.2021 |  |  | Denise Wawman |
| FF122 | | O.fringillina | | | | UNITED KINGDOM | ♂ | Somerset | 2 | Minehead, Bratton | 64 | 4.viii.2021 | Dunnock | *Prunella modularis* | Denise Wawman |
| FF124 | | O.fringillina | | | | UNITED KINGDOM | ♂ | Somerset | 3 | Minehead, Bratton | 64 | 3.viii.2021 |  |  | Denise Wawman |
| FF125 | | O.fringillina | | | | UNITED KINGDOM |  | Somerset | 3 | Minehead, Bratton | 64 | 10.viii.2021 | Robin | *Erithacus rubecula* | Denise Wawman |
| FF127 | | O.fringillina | | | | UNITED KINGDOM |  | Somerset | 2 | Minehead, Bratton | 64 | 12.viii.2021 | Robin | *Erithacus rubecula* | Denise Wawman |
| FF128 | | O.fringillina | | | | UNITED KINGDOM | ♀ | Somerset | 3 | Minehead, Bratton | 64 | 12.viii.2021 | Robin | *Erithacus rubecula* | Denise Wawman |
| FF130 | | O.fringillina | | | | UNITED KINGDOM |  | Somerset | 2 | Minehead, Bratton | 64 | 12.viii.2021 |  |  | Denise Wawman |
| FF131 | | O.fringillina | | | | UNITED KINGDOM |  | Somerset | 3 | Minehead, Bratton | 64 | 19.viii.2021 | Siskin | *Spinus spinus* | Denise Wawman |
| FF132 | | O.fringillina | | | | UNITED KINGDOM | ♂ | Somerset | 3 | Minehead, Bratton | 64 | 19.viii.2021 | Blue Tit | *Cyanistes caeruleus* | Denise Wawman |
| FF133 | | O.fringillina | | | | UNITED KINGDOM | ♂ | Somerset | 3 | Minehead, Bratton | 64 | 22.viii.2021 | Robin | *Erithacus rubecula* | Denise Wawman |
| FF134 | | O.fringillina | | | | UNITED KINGDOM | ♀ | Somerset | 3 | Minehead, Bratton | 64 | 22.viii.2021 | Robin | *Erithacus rubecula* | Denise Wawman |
| FF135 | | O.fringillina | | | | UNITED KINGDOM | ♂ | Somerset | 3 | Minehead, Bratton | 64 | 22.viii.2021 |  |  | Denise Wawman |
| FF136 | | O.fringillina | | | | UNITED KINGDOM |  | Somerset | 2 | Minehead, Bratton | 64 | 22.viii.2021 |  |  | Denise Wawman |
| FF139 | | O.fringillina | | | | UNITED KINGDOM | ♀ | Somerset | 3 | Minehead, Bratton | 64 | 3.ix.2021 | Greenfinch | *Chloris chloris* | Denise Wawman |
| FF140 | | O.fringillina | | | | UNITED KINGDOM | ♂ | Somerset |  | Minehead, Bratton | 64 | 5.ix.2021 | Robin | *Erithacus rubecula* | Denise Wawman |
| FF141 | | O.fringillina | | | | UNITED KINGDOM | ♂ | Somerset |  | Minehead, Bratton | 64 | 21.ix.2021 | Robin | *Erithacus rubecula* | Denise Wawman |
| FF142 | | O.fringillina | | | | UNITED KINGDOM | ♀ | Somerset | 3 | Minehead, Bratton | 64 | 21.ix.2021 | Great Tit | *Parus major* | Denise Wawman |
| FF143 | | O.fringillina | | | | UNITED KINGDOM | ♂ | Somerset | 3 | Minehead, Bratton | 64 | 21.ix.2021 | Great Tit | *Parus major* | Denise Wawman |
| FF144 | | O.fringillina | | | | UNITED KINGDOM | ♂ | Somerset | 2 | Minehead, Bratton | 64 | 25.ix.2021 | Blue Tit | *Cyanistes caeruleus* | Denise Wawman |
| FF145 | | O.fringillina | | | | UNITED KINGDOM | ♀ | Somerset | 3 | Minehead, Bratton | 64 | 23.x.2021 | Goldfinch | *Carduelis carduelis* | Denise Wawman |
| FF152 | | O.fringillina | | | | UNITED KINGDOM | ♂ | Somerset |  | Minehead, Bratton | 64 | 12.vi.2022 | Blue Tit | *Cyanistes caeruleus* | Denise Wawman |
| FF154 | | O.fringillina | | | | UNITED KINGDOM | ♂ | Somerset | 2 | Minehead, Bratton | 64 | 16.vi.2022 | Great Tit | *Parus major* | Denise Wawman |
| FF158 | | O.fringillina | | | | UNITED KINGDOM |  | Somerset | 3 | Minehead, Bratton | 64 | 15.vii.2022 | Dunnock | *Prunella modularis* | Denise Wawman |
| FF162 | | O.fringillina | | | | UNITED KINGDOM |  | Somerset | 2 | Webbers Post | 246 | 20.vii.2022 | Robin | *Erithacus rubecula* | Denise Wawman |
| FF165 | | O.fringillina | | | | UNITED KINGDOM | ♀ | Somerset | 3 | Webbers Post | 245 | 29.vii.2022 | Robin | *Erithacus rubecula* | Denise Wawman |
| FF166 | | O.fringillina | | | | UNITED KINGDOM | ♀ | Somerset | 3 | Minehead, Bratton | 64 | 30.vii.2022 | Robin | *Erithacus rubecula* | Denise Wawman |
| FF169 | | O.fringillina | | | | UNITED KINGDOM | ♂ | Somerset | 2 | Minehead, Bratton | 64 | 30.vii.2022 |  |  | Denise Wawman |
| FF22 | | O.fringillina | | | | UNITED KINGDOM |  | Somerset | 3 | Minehead, Bratton | 64 | 7.viii.2013 | Dunnock | *Prunella modularis* | Denise Wawman |
| FF24 | | O.fringillina | | | | UNITED KINGDOM |  | Somerset | 3 | Minehead, Bratton | 64 | 16.viii.2014 | Chiffchaff | *Phylloscopus collybita* | Denise Wawman |
| FF39 | | O.fringillina | | | | UNITED KINGDOM |  | Somerset | 3 | Minehead, Bratton | 64 | 20.vii.2020 |  |  | Denise Wawman |
| FF41 | | O.fringillina | | | | UNITED KINGDOM |  | Somerset | 3 | Minehead, Bratton | 64 | 21.vii.2020 |  |  | Denise Wawman |
| FF44 | | O.fringillina | | | | UNITED KINGDOM |  | Somerset |  | Minehead, Bratton | 64 | 24.vii.2020 | Goldfinch | *Carduelis carduelis* | Denise Wawman |
| FF46 | | O.fringillina | | | | UNITED KINGDOM | ♂ | Somerset | 3 | Minehead, Bratton | 64 | 24.vii.2020 |  |  | Denise Wawman |
| FF47 | | O.fringillina | | | | UNITED KINGDOM | ♀ | Somerset | 3 | Minehead, Bratton | 64 | 24.vii.2020 |  |  | Denise Wawman |
| FF51 | | O.fringillina | | | | UNITED KINGDOM | ♂ | Somerset |  | Minehead, Bratton | 64 | 2.viii.2020 | Goldfinch | *Carduelis carduelis* | Denise Wawman |
| FF57 | | O.fringillina | | | | UNITED KINGDOM | ♂ | Somerset | 3 | Minehead, Bratton | 64 | 8.viii.2020 |  |  | Denise Wawman |
| FF58 | | O.fringillina | | | | UNITED KINGDOM |  | Somerset | 3 | Minehead, Bratton | 64 | 8.viii.2020 |  |  | Denise Wawman |
| FF61 | | O.fringillina | | | | UNITED KINGDOM | ♀ | Somerset | 2 | Minehead, Bratton | 64 | 17.viii.2020 | Goldfinch | *Carduelis carduelis* | Denise Wawman |
| FF64 | | O.fringillina | | | | UNITED KINGDOM |  | Somerset | 2 | Minehead, Bratton | 64 | 31.viii.2020 | Dunnock | *Prunella modularis* | Denise Wawman |
| FF65 | | O.fringillina | | | | UNITED KINGDOM |  | Somerset | 3 | Minehead, Bratton | 64 | 31.viii.2020 |  |  | Denise Wawman |
| FF66 | | O.fringillina | | | | UNITED KINGDOM | ♂ | Somerset | 3 | Minehead, Bratton | 64 | 31.viii.2020 | Goldfinch | *Carduelis carduelis* | Denise Wawman |
| FF67 | | O.fringillina | | | | UNITED KINGDOM |  | Somerset | 3 | Minehead, Bratton | 64 | 31.viii.2020 | Goldfinch | *Carduelis carduelis* | Denise Wawman |
| FF68 | | O.fringillina | | | | UNITED KINGDOM |  | Somerset | 3 | Minehead, Bratton | 64 | 31.viii.2020 | Goldfinch | *Carduelis carduelis* | Denise Wawman |
| FF69 | | O.fringillina | | | | UNITED KINGDOM |  | Somerset | 3 | Minehead, Bratton | 64 | 10.ix.2020 | Goldfinch | *Carduelis carduelis* | Denise Wawman |
| FF70 | | O.fringillina | | | | UNITED KINGDOM | ♀ | Somerset | 3 | Minehead, Bratton | 64 | 10.ix.2020 | Blue Tit | *Cyanistes caeruleus* | Denise Wawman |
| MG02 | | O.fringillina | | | | UNITED KINGDOM | ♂ | Norfolk | 3 | Hindolveston | 66 | 4.viii.2018 | Dunnock | *Prunella modularis* | Charles Dewhurst |
| MG16 | | O.fringillina | | | | UNITED KINGDOM | ♂ | Norfolk | 3 | Field Dalling | 50 | 1.ix.2019 | Long-tailed Tit | *Aegithalos caudatus* | Charles Dewhurst |
| MG18A | | O.fringillina | | | | UNITED KINGDOM | ♂ | Norfolk | 3 | Field Dalling | 50 | 26.vii.2020 | Goldfinch | *Carduelis carduelis* | Charles Dewhurst |
| MG18B | | O.fringillina | | | | UNITED KINGDOM |  | Norfolk | 2 | Field Dalling | 50 | 26.vii.2020 | Goldfinch | *Carduelis carduelis* | Charles Dewhurst |
| MG22 | | O.fringillina | | | | UNITED KINGDOM | ♀ | Norfolk | 2 | Field Dalling | 50 | 16.viii.2020 | Goldfinch | *Carduelis carduelis* | Charles Dewhurst |
| MG23 | | O.fringillina | | | | UNITED KINGDOM |  | Norfolk | 3 | Field Dalling | 50 | 24.viii.2020 | Goldfinch | *Carduelis carduelis* | Charles Dewhurst |
| NW01 | | O.fringillina | | | | UNITED KINGDOM | ♀ | Devon |  | Slapton Ley | 4 | 6.ix.2020 | Blackcap | *Sylvia atricapilla* | Nik Ward |
| NW05 | | O.fringillina | | | | UNITED KINGDOM | ♂ | Devon | 2 | Slapton | 10 | 5.ix.2021 | Robin | *Erithacus rubecula* | Nik Ward |
| NW06 | | O.fringillina | | | | UNITED KINGDOM | ♀ | Devon | 2 | Slapton | 10 | 14.viii.2021 | Linnet | *Linaria cannabina* | Nik Ward |
| NW07 | | O.fringillina | | | | UNITED KINGDOM |  | Devon | 3 | Ide | 20 | 24.ix.2021 | Greenfinch | *Chloris chloris* | Nik Ward |
| NW08 | | O.fringillina | | | | UNITED KINGDOM | ♂ | Devon | 3 | Ide | 20 | 17.viii.2021 |  |  | Nik Ward |
| NW09 | | O.fringillina | | | | UNITED KINGDOM | ♂ | Devon | 2 | Slapton | 10 | 23.viii.2021 | Cetti's Warbler | *Cettia cetti* | Nik Ward |
| SB24 | | O.fringillina | | | | IRELAND | ♂ | Cork | 2 | Enniskeane | 99 | 15.vii.2021 | Goldcrest | *Regulus regulus* | Sam Bayley |
| SB29 | | O.fringillina | | | | IRELAND |  | Cork | 3 | Owenahincha, Little Island Wetland | 0 | 26.viii.2021 | Sedge Warbler | *Acrocephalus schoenobaenus* | Sam Bayley |
| SB36 | | O.fringillina | | | | IRELAND | ♂ | Cork | 3 | Owenahincha, Little Island Wetland | 0 | 26.viii.2021 | Chiffchaff | *Phylloscopus collybita* | Sam Bayley |
| SB39 | | O.fringillina | | | | IRELAND | ♀ | Cork | 3 | Owenahincha, Little Island Wetland | 0 | 17.viii.2021 | Robin | *Erithacus rubecula* | Sam Bayley |
| SB40 | | O.fringillina | | | | IRELAND | ♀ | Cork | 3 | Owenahincha, Little Island Wetland | 0 | 26.viii.2021 | Chiffchaff | *Phylloscopus collybita* | Sam Bayley |
| SB59 | | O.fringillina | | | | IRELAND | ♀ | Cork | 3 | Enniskeane | 99 | 31.viii.2021 | Chaffinch | *Fringilla coelebs* | Sam Bayley |
| SB61 | | O.fringillina | | | | IRELAND | ♂ | Cork | 3 | Enniskeane | 99 | 24.viii.2021 | Robin | *Erithacus rubecula* | Sam Bayley |
| SB62 | | O.fringillina | | | | IRELAND | ♂ | Cork | 3 | Enniskeane | 99 | 31.viii.2021 | Robin | *Erithacus rubecula* | Sam Bayley |
| SB63 | | O.fringillina | | | | IRELAND | ♀ | Cork | 3 | Enniskeane | 99 | 6.ix.2021 | Great Tit | *Parus major* | Sam Bayley |
| SB64 | | O.fringillina | | | | IRELAND |  | Cork | 3 | Enniskeane | 99 | 22.xi.2021 | Goldfinch | *Carduelis carduelis* | Sam Bayley |
| SB65 | | O.fringillina | | | | IRELAND | ♀ | Cork | 3 | Enniskeane | 99 | 24.viii.2021 |  |  | Sam Bayley |
| SB66 | | O.fringillina | | | | IRELAND | ♀ | Cork | 3 | Enniskeane | 99 | 24.viii.2021 | Chaffinch | *Fringilla coelebs* | Sam Bayley |
| SB67 | | O.fringillina | | | | IRELAND | ♀ | Cork |  | Enniskeane | 99 | 6.ix.2021 | Chiffchaff | *Phylloscopus collybita* | Sam Bayley |
| SB69 | | O.fringillina | | | | IRELAND |  | Cork |  | Enniskeane | 99 | 5.xi.2021 | Goldcrest | *Regulus regulus* | Sam Bayley |
| SB70 | | O.fringillina | | | | IRELAND | ♀ | Cork | 3 | Enniskeane | 99 | 19.viii.2021 | House Sparrow | *Passer domesticus* | Sam Bayley |
| SB71 | | O.fringillina | | | | IRELAND |  | Cork | 3 | Enniskeane | 99 | 31.viii.2021 | Goldcrest | *Regulus regulus* | Sam Bayley |
| SB73 | | O.fringillina | | | | IRELAND |  | Cork | 3 | Enniskeane | 99 | 31.viii.2021 | Willow Warbler | *Phylloscopus trochilus* | Sam Bayley |
| SB78 | | O.fringillina | | | | IRELAND | ♂ | Cork |  | Enniskeane | 99 | 8.viii.2021 | Robin | *Erithacus rubecula* | Sam Bayley |
| SB80 | | O.fringillina | | | | IRELAND | ♂ | Cork | 3 | Enniskeane | 99 | 19.viii.2021 | House Sparrow | *Passer domesticus* | Sam Bayley |
| SB81 | | O.fringillina | | | | IRELAND | ♂ | Cork | 3 | Enniskeane | 99 | 8.viii.2021 | Robin | *Erithacus rubecula* | Sam Bayley |
| SB85 | | O.fringillina | | | | IRELAND | ♀ | Cork | 3 | Enniskeane | 99 | 31.viii.2021 | Blackcap | *Sylvia atricapilla* | Sam Bayley |
| SB86 | | O.fringillina | | | | IRELAND | ♀ | Cork | 3 | Enniskeane | 99 | 10.viii.2021 |  |  | Sam Bayley |
| SB88 | | O.fringillina | | | | IRELAND |  | Cork | 3 | Enniskeane | 99 | 24.viii.2021 | Dunnock | *Prunella modularis* | Sam Bayley |
| SB89 | | O.fringillina | | | | IRELAND | ♀ | Cork | 3 | Enniskeane | 99 | 31.viii.2021 | Treecreeper | *Certhia familiaris* | Sam Bayley |
| SB91 | | O.fringillina | | | | IRELAND | ♀ | Cork | 3 | Enniskeane | 99 | 31.vii.2021 | Dunnock | *Prunella modularis* | Sam Bayley |
| SB92 | | O.fringillina | | | | IRELAND | ♀ | Cork | 3 | Enniskeane | 99 | 31.viii.2021 | Treecreeper | *Certhia familiaris* | Sam Bayley |
| SB93 | | O.fringillina | | | | IRELAND | ♀ | Cork | 3 | Enniskeane | 99 | 24.viii.2021 | Song Thrush | *Turdus philomelos* | Sam Bayley |
| SB95 | | O.fringillina | | | | IRELAND | ♂ | Cork | 3 | Enniskeane | 99 | 23.vii.2021 |  |  | Sam Bayley |
| FF170 | | O.fringillina | | | | UNITED KINGDOM | ♂ | Somerset | 3 | Minehead, Bratton | 64 | 1.viii.2022 | Great Tit | *Parus major* | Denise Wawman |
| FF174 | | O.fringillina | | | | UNITED KINGDOM | ♀ | Somerset | BSmart | Minehead, Bratton | 64 | 1.viii.2022 | Blue Tit | *Cyanistes caeruleus* | Denise Wawman |
| 7188 | | O.fringillina | | | | UNITED KINGDOM | ♀ | Norfolk | BSmart | Cranwich | 10 | 23.vii.2022 | Reed Warbler | *Acrocephalus scirpaceus* | Lee Barber |
| H181 | | O.fringillina | | | | UNITED KINGDOM | ♀ | Inverness-shire | ASmart | Carse of Ardersier | 5 | 23.vii.2022 | Willow Warbler | *Phylloscopus trochilus* | Hugh Insley |
| H182 | | O.fringillina | | | | UNITED KINGDOM | ♀ | Inverness-shire | ASmart | Carse of Ardersier | 5 | 27.vii.2022 | Willow Warbler | *Phylloscopus trochilus* | Hugh Insley |
| H183 | | O.fringillina | | | | UNITED KINGDOM | ♂ | Inverness-shire | BSmart | Carse of Ardersier | 5 | 28.vii.2022 | Willow Warbler | *Phylloscopus trochilus* | Hugh Insley |
| H184 | | O.fringillina | | | | UNITED KINGDOM | ♂ | Inverness-shire | BSmart | Carse of Ardersier | 5 | 27.vii.2022 | Willow Warbler | *Phylloscopus trochilus* | Hugh Insley |
| H185 | | O.fringillina | | | | UNITED KINGDOM | ♀ | Inverness-shire | BSmart | Carse of Ardersier | 5 | 28.vii.2022 |  |  | Hugh Insley |
| H186 | | O.fringillina | | | | UNITED KINGDOM |  | Inverness-shire | ASmart | Carse of Ardersier | 5 | 27.vii.2022 | Robin | *Erithacus rubecula* | Hugh Insley |
| H189 | | O.fringillina | | | | UNITED KINGDOM |  | Inverness-shire | ASmart | Carse of Ardersier | 5 | 27.vii.2022 | Willow Warbler | *Phylloscopus trochilus* | Hugh Insley |
| H191 | | O.fringillina | | | | UNITED KINGDOM |  | Inverness-shire | BSmart | Carse of Ardersier | 5 | 28.vii.2022 | Willow Warbler | *Phylloscopus trochilus* | Hugh Insley |
| H194 | | O.fringillina | | | | UNITED KINGDOM | ♂ | Inverness-shire | BSmart | Carse of Ardersier | 5 | 27.vii.2022 | Willow Warbler | *Phylloscopus trochilus* | Hugh Insley |
| H195 | | O.fringillina | | | | UNITED KINGDOM | ♀ | Inverness-shire | BSmart | Carse of Ardersier | 5 | 28.vii.2022 | Chiffchaff | *Phylloscopus collybita* | Hugh Insley |
| H198 | | O.fringillina | | | | UNITED KINGDOM | ♂ | Inverness-shire | Hutson | Carse of Ardersier | 5 | 28.vii.2022 | Willow Warbler | *Phylloscopus trochilus* | Hugh Insley |
| H200A | | O.fringillina | | | | UNITED KINGDOM | ♂ | Inverness-shire | BSmart | Carse of Ardersier | 5 | 28.vii.2022 | Willow Warbler | *Phylloscopus trochilus* | Hugh Insley |
| H200B | | O.fringillina | | | | UNITED KINGDOM | ♂ | Inverness-shire | ASmart | Carse of Ardersier | 5 | 28.vii.2022 | Willow Warbler | *Phylloscopus trochilus* | Hugh Insley |
| 7715 | | O.fringillina | | | | UNITED KINGDOM | ♀ | Inverness-shire | ASmart | Carse of Ardersier | 5 | 31.vii.2022 | Willow Warbler | *Phylloscopus trochilus* | Hugh Insley |
| 7704 | | O.fringillina | | | | UNITED KINGDOM |  | Inverness-shire | ASmart | Carse of Ardersier | 5 | 31.vii.2022 | Goldcrest | *Regulus regulus* | Hugh Insley |
| 7708 | | O.fringillina | | | | UNITED KINGDOM |  | Inverness-shire | BSmart | Carse of Ardersier | 5 | 31.vii.2022 | Willow Warbler | *Phylloscopus trochilus* | Hugh Insley |
| 7709 | | O.fringillina | | | | UNITED KINGDOM |  | Inverness-shire | ASmart | Carse of Ardersier | 5 | 31.vii.2022 | Willow Warbler | *Phylloscopus trochilus* | Hugh Insley |
| 7713 | | O.fringillina | | | | UNITED KINGDOM | ♂ | Inverness-shire | BSmart | Carse of Ardersier | 5 | 31.vii.2022 | Willow Warbler | *Phylloscopus trochilus* | Hugh Insley |
| FF175 | | O.fringillina | | | | UNITED KINGDOM | ♀ | Somerset | ASmart | Minehead, North Hill | 251 | 10.viii.2022 | Stonechat | *Saxicola rubicola* | Denise Wawman |
| FF178 | | O.fringillina | | | | UNITED KINGDOM | ♀ | Somerset | ASmart | Minehead, Selworthy Beacon | 290 | 27.viii.2022 |  |  | Denise Wawman |
| 7702 | | O.fringillina | | | | UNITED KINGDOM | ♂ | Inverness-shire | BSmart | Carse of Ardersier | 5 | 12.viii.2022 | Blackcap | *Sylvia atricapilla* | Hugh Insley |
| 7705 | | O.fringillina | | | | UNITED KINGDOM | ♀ | Inverness-shire | BSmart | Carse of Ardersier | 5 | 13.viii.2022 | Willow Warbler | *Phylloscopus trochilus* | Hugh Insley |
| 7706 | | O.fringillina | | | | UNITED KINGDOM | ♂ | Inverness-shire | Hutson | Carse of Ardersier | 5 | 13.viii.2022 | Willow Warbler | *Phylloscopus trochilus* | Hugh Insley |
| 7707 | | O.fringillina | | | | UNITED KINGDOM | ♂ | Inverness-shire | BSmart | Carse of Ardersier | 5 | 13.viii.2022 | Willow Warbler | *Phylloscopus trochilus* | Hugh Insley |
| 7712 | | O.fringillina | | | | UNITED KINGDOM |  | Inverness-shire | ASmart | Carse of Ardersier | 5 | 13.viii.2022 | Willow Warbler | *Phylloscopus trochilus* | Hugh Insley |
| 7928 | | O.fringillina | | | | UNITED KINGDOM | ♀ | Inverness-shire | BSmart | Inverness, Drummond | 34 | 15.viii.2022 | Blue Tit | *Cyanistes caeruleus* | Hugh Insley |
| 7716 | | O.fringillina | | | | UNITED KINGDOM | ♀ | Inverness-shire | ASmart | Carse of Ardersier | 5 | 22.viii.2022 | Goldcrest | *Regulus regulus* | Hugh Insley |
| 7717 | | O.fringillina | | | | UNITED KINGDOM | ♀ | Inverness-shire | BSmart | Carse of Ardersier | 5 | 22.viii.2022 | Goldcrest | *Regulus regulus* | Hugh Insley |
| 7719 | | O.fringillina | | | | UNITED KINGDOM | ♀ | Inverness-shire | ASmart | Carse of Ardersier | 5 | 24.viii.2022 | Robin | *Erithacus rubecula* | Hugh Insley |
| 7718 | | O.fringillina | | | | UNITED KINGDOM | ♂ | Inverness-shire | Hutson | Carse of Ardersier | 5 | 24.viii.2022 | Song Thrush | *Turdus philomelos* | Hugh Insley |
| H187 | | O.fringillina | | | | UNITED KINGDOM |  | Inverness-shire | ASmart | Carse of Ardersier | 5 | 11.viii.2022 | Willow Warbler | *Phylloscopus trochilus* | Hugh Insley |
| H190 | | O.fringillina | | | | UNITED KINGDOM |  | Inverness-shire | ASmart | Carse of Ardersier | 5 | 11.viii.2022 | Chiffchaff | *Phylloscopus collybita* | Hugh Insley |
| H192 | | O.fringillina | | | | UNITED KINGDOM |  | Inverness-shire |  | Carse of Ardersier | 5 | 4.viii.2022 | Willow Warbler | *Phylloscopus trochilus* | Hugh Insley |
| H193 | | O.fringillina | | | | UNITED KINGDOM | ♂ | Inverness-shire | ASmart | Carse of Ardersier | 5 | 4.viii.2022 | Chiffchaff | *Phylloscopus collybita* | Hugh Insley |
| H196 | | O.fringillina | | | | UNITED KINGDOM | ♀ | Inverness-shire | ASmart | Carse of Ardersier | 5 | 11.viii.2022 | Willow Warbler | *Phylloscopus trochilus* | Hugh Insley |
| H197 | | O.fringillina | | | | UNITED KINGDOM |  | Inverness-shire | ASmart | Carse of Ardersier | 5 | 11.viii.2022 | Willow Warbler | *Phylloscopus trochilus* | Hugh Insley |
| H199 | | O.fringillina | | | | UNITED KINGDOM | ♀ | Inverness-shire | BSmart | Carse of Ardersier | 5 | 11.viii.2022 | Willow Warbler | *Phylloscopus trochilus* | Hugh Insley |
| FF184 | | O.fringillina | | | | UNITED KINGDOM | ♂ | Somerset | ASmart | Minehead, Bratton | 64 | 1.ix.2022 | Blue Tit | *Cyanistes caeruleus* | Denise Wawman |
| 7621 | | O.fringillina | | | | UNITED KINGDOM | ♀ | Skye | Hutson | Portree | 40 | 25.viii.2022 | Siskin | *Spinus spinus* | Jonathan Jones |
| 7622 | | O.fringillina | | | | UNITED KINGDOM | ♂ | Skye | Hutson | Portree | 40 | 25.viii.2022 | Goldfinch | *Carduelis carduelis* | Jonathan Jones |
| 7627 | | O.fringillina | | | | UNITED KINGDOM | ♂ | Skye | BSmart | Portree | 40 | 3.ix.2022 | Siskin | *Spinus spinus* | Jonathan Jones |
| 7628 | | O.fringillina | | | | UNITED KINGDOM | ♂ | Skye | Hutson | Varagill | 60 | 6.ix.2022 | Goldfinch | *Carduelis carduelis* | Jonathan Jones |
| 7629 | | O.fringillina | | | | UNITED KINGDOM | ♀ | Skye | BSmart | Varagill | 60 | 6.ix.2022 | Robin | *Erithacus rubecula* | Jonathan Jones |
| 7630 | | O.fringillina | | | | UNITED KINGDOM | ♀ | Skye | BSmart | Varagill | 60 | 6.ix.2022 | Great Tit | *Parus major* | Jonathan Jones |
| 7633 | | O.fringillina | | | | UNITED KINGDOM | ♀ | Skye | Hutson | Hungladder | 40 | 28.viii.2022 | Chaffinch | *Fringilla coelebs* | Jonathan Jones |
| 7636 | | O.fringillina | | | | UNITED KINGDOM | ♂ | Skye | ASmart | Varagill | 60 | 4.ix.2022 | Goldcrest | *Regulus regulus* | Jonathan Jones |
| 7637 | | O.fringillina | | | | UNITED KINGDOM | ♀ | Skye | ASmart | Varagill | 60 | 4.ix.2022 | Great Tit | *Parus major* | Jonathan Jones |
| 7638 | | O.fringillina | | | | UNITED KINGDOM | ♂ | Skye | BSmart | Varagill | 60 | 4.ix.2022 | Bullfinch | *Pyrrhula pyrrhula* | Jonathan Jones |
| 7639 | | O.fringillina | | | | UNITED KINGDOM |  | Skye | BSmart | Varagill | 60 | 4.ix.2022 | Great Tit | *Parus major* | Jonathan Jones |
| 7640 | | O.fringillina | | | | UNITED KINGDOM | ♂ | Skye | Hutson | Varagill | 60 | 4.ix.2022 | Goldfinch | *Carduelis carduelis* | Jonathan Jones |
| 7649 | | O.fringillina | | | | UNITED KINGDOM | ♂ | Skye | BSmart | Hungladder | 40 | 31.vii.2022 | Meadow Pipit | *Anthus pratensis* | Jonathan Jones |
| 7652 | | O.fringillina | | | | UNITED KINGDOM |  | Skye | damaged | Portree | 40 | 9.viii.2022 | Goldfinch | *Carduelis carduelis* | Jonathan Jones |
| 7653 | | O.fringillina | | | | UNITED KINGDOM | ♀ | Skye | ASmart | Portree | 40 | 11.viii.2022 | Siskin | *Spinus spinus* | Jonathan Jones |
| 7658 | | O.fringillina | | | | UNITED KINGDOM | ♂ | Skye | Hutson | Portree | 40 | 11.viii.2022 | Greenfinch | *Chloris chloris* | Jonathan Jones |
| 7659 | | O.fringillina | | | | UNITED KINGDOM |  | Skye | BSmart | Portree | 40 | 20.viii.2022 | Goldfinch | *Carduelis carduelis* | Jonathan Jones |
| 7541 | | O.fringillina | | | | UNITED KINGDOM | ♀ | Skye | damaged | Varagill | 60 | 2.xi.2021 | Chaffinch | *Fringilla coelebs* | Jonathan Jones |
| 360 | | O.fringillina | | | | UNITED KINGDOM | ♀ | Staffordshire | ASmart | Walsall, Daisybank | 148 | 23.vii.2022 | Long-tailed Tit | *Aegithalos caudatus* | David Clifton |
| 348 | | O.fringillina | | | | UNITED KINGDOM | ♀ | Warwickshire | ASmart | Hams Hall | 72 | 27.vii.2022 | Reed Warbler | *Acrocephalus scirpaceus* | David Clifton |
| 352 | | O.fringillina | | | | UNITED KINGDOM | ♀ | Warwickshire | ASmart | Hams Hall | 72 | 5.viii.2022 | Blackcap | *Sylvia atricapilla* | David Clifton |
| 349 | | O.fringillina | | | | UNITED KINGDOM | ♂ | Warwickshire | BSmart | Hams Hall | 74 | 10.viii.2022 |  |  | David Clifton |
| P33A | | O.fringillina | | | | UNITED KINGDOM | ♂ | Warwickshire | BSmart | Hams Hall | 75 | 26.viii.2022 | Reed Warbler | *Acrocephalus scirpaceus* | David Clifton |
| P33B | | O.fringillina | | | | UNITED KINGDOM | ♀ | Warwickshire | ASmart | Hams Hall | 75 | 26.viii.2022 | Reed Warbler | *Acrocephalus scirpaceus* | David Clifton |
| 357 | | O.fringillina | | | | UNITED KINGDOM | ♀ | Warwickshire | Hutson | Coleshill | 76 | 24.ix.2022 | Dunnock | *Prunella modularis* | David Clifton |
| 344 | | O.fringillina | | | | UNITED KINGDOM | ♀ | Staffordshire | ASmart | Walsall, Daisybank | 148 | 11.x.2022 | Goldfinch | *Carduelis carduelis* | David Clifton |
| 7714 | | O.fringillina | | | | UNITED KINGDOM | ♀ | Inverness-shire | ASmart | Carse of Ardersier | 3 | 22.viii.2022 | Blackcap | *Sylvia atricapilla* | Hugh Insley |
| 7711 | | O.fringillina | | | | UNITED KINGDOM | ♂ | Inverness-shire | ASmart | Carse of Ardersier | 3 | 30.viii.2022 | Robin | *Erithacus rubecula* | Hugh Insley |
| 7720 | | O.fringillina | | | | UNITED KINGDOM | ♂ | Inverness-shire | BSmart | Carse of Ardersier | 3 | 10.ix.2022 | Great Tit | *Parus major* | Hugh Insley |
| H141 | | O.fringillina | | | | UNITED KINGDOM | ♀ | Inverness-shire | BSmart | Carse of Ardersier | 3 | 14.ix.2022 | Goldcrest | *Regulus regulus* | Hugh Insley |
| H064 | | O.fringillina | | | | UNITED KINGDOM | ♀ | Dumfries and Galloway | BSmart | Stranraer, Leswalt | 35 | 8.viii.2022 | Wren | *Troglodytes troglodytes* | Geoff & Jean Sheppard |
| H065 | | O.fringillina | | | | UNITED KINGDOM | ♀ | Dumfries and Galloway | BSmart | Stranraer, Leswalt | 35 | 8.viii.2022 | Wren | *Troglodytes troglodytes* | Geoff & Jean Sheppard |
| H067 | | O.fringillina | | | | UNITED KINGDOM | ♂ | Dumfries and Galloway | Hutson | Stranraer, Leswalt | 35 | 12.viii.2022 | Chaffinch | *Fringilla coelebs* | Geoff & Jean Sheppard |
| H069 | | O.fringillina | | | | UNITED KINGDOM | ♀ | Dumfries and Galloway | BSmart | Stranraer, Leswalt | 35 | 26.viii.2022 | Lesser Redpoll | *Acanthis cabaret* | Geoff & Jean Sheppard |
| H070 | | O.fringillina | | | | UNITED KINGDOM | ♀ | Dumfries and Galloway | BSmart | Stranraer, Leswalt | 35 | 26.viii.2022 | Siskin | *Spinus spinus* | Geoff & Jean Sheppard |
| H073 | | O.fringillina | | | | UNITED KINGDOM | ♀ | Dumfries and Galloway | ASmart | Stranraer, Leswalt | 35 | 2.ix.2022 | Greenfinch | *Chloris chloris* | Geoff & Jean Sheppard |
| H074 | | O.fringillina | | | | UNITED KINGDOM | ♀ | Dumfries and Galloway | BSmart | Stranraer, Leswalt | 35 | 8.ix.2022 | Great Tit | *Parus major* | Geoff & Jean Sheppard |
| H075 | | O.fringillina | | | | UNITED KINGDOM | ♀ | Dumfries and Galloway | ASmart | Stranraer, Leswalt | 35 | 8.ix.2022 | Robin | *Erithacus rubecula* | Geoff & Jean Sheppard |
| 3239 | | O.fringillina | | | | UNITED KINGDOM | ♂ | Argyll | ASmart | Inveraray, Lagganbeg | 93 | 10.viii.2022 | House Sparrow | *Passer domesticus* | Rob Lightfoot |
| 7332 | | O.fringillina | | | | UNITED KINGDOM | ♀ | Devon | ASmart | Ilfracombe, West Down, Buttercombe Barton | 160 | 1.vii.2022 | Chaffinch | *Fringilla coelebs* | Chris Dee |
| 7336 | | O.fringillina | | | | UNITED KINGDOM | ♂ | Devon | ASmart | Ilfracombe, West Down, Buttercombe Barton | 160 | 7.vii.2022 | Greenfinch | *Chloris chloris* | Chris Dee |
| 7581 | | O.fringillina | | | | UNITED KINGDOM | ♀ | Devon | BSmart | Ilfracombe, West Down, Buttercombe Barton | 160 | 1.viii.2022 | Robin | *Erithacus rubecula* | Chris Dee |
| 7583 | | O.fringillina | | | | UNITED KINGDOM | ♀ | Devon | damaged | Ilfracombe, West Down, Buttercombe Barton | 160 | 20.viii.2022 |  |  | Chris Dee |
| 547 | | O.fringillina | | | | UNITED KINGDOM | ♀ | West Sussex | BSmart | Ladywell Valley | 15 | 9.viii.2022 |  |  | Clare Buckle |
| 550 | | O.fringillina | | | | UNITED KINGDOM | ♀ | West Sussex | ASmart | Ladywell Valley | 15 |  | Goldcrest | *Regulus regulus* | Clare Buckle |
| H005 | | O.fringillina | | | | UNITED KINGDOM | ♀ | Suffolk | ASmart | Brandon | 25 | 7.ix.2022 | Goldfinch | *Carduelis carduelis* | Dr Greg Conway |
| 5122 | | O.fringillina | | | | UNITED KINGDOM | ♀ | Suffolk | ASmart | Brandon | 25 | 4.x.2022 | Siskin | *Spinus spinus* | Dr Greg Conway |
| 7206 | | O.fringillina | | | | UNITED KINGDOM | ♀ | Norfolk | BSmart | Rosedene | 10 | 19.viii.2022 | Reed Warbler | *Acrocephalus scirpaceus* | Lee Barber |
| 7201 | | O.fringillina | | | | UNITED KINGDOM | ♀ | Norfolk | BSmart | Cranwich | 10 | 21.viii.2022 | Reed Warbler | *Acrocephalus scirpaceus* | Lee Barber |
| 7209 | | O.fringillina | | | | UNITED KINGDOM | ♀ | Denibighshire | ASmart | Garth | 200 | 28.viii.2022 | Dunnock | *Prunella modularis* | Lee Barber |
| 7203 | | O.fringillina | | | | UNITED KINGDOM | ♀ | Norfolk | BSmart | Cranwich | 10 | 4.ix.2022 | Reed Warbler | *Acrocephalus scirpaceus* | Lee Barber |
| 7212 | | O.fringillina | | | | UNITED KINGDOM | ♀ | Norfolk | Hutson | Croxton Park | 20 | 7.x.2022 | Skylark | *Alauda arvensis* | Lee Barber |
| 2942 | | O.fringillina | | | | UNITED KINGDOM | ♂ | Somerset | BSmart | Camelby | 120 | 1.ix.2022 | Dunnock | *Prunella modularis* | Bob Medland |
| 1566 | | O.fringillina | | | | UNITED KINGDOM | ♀ | Northamptonshire | ASmart | Stanford Reservoir | 113 | 16.vii.2022 | Dunnock | *Prunella modularis* | Stanford Ringing Group |
| 1570 | | O.fringillina | | | | UNITED KINGDOM | ♀ | Northamptonshire | ASmart | Stanford Reservoir | 113 | 6.viii.2022 | Reed Warbler | *Acrocephalus scirpaceus* | Stanford Ringing Group |
| 1571 | | O.fringillina | | | | UNITED KINGDOM | ♀ | Northamptonshire | Hutson | Stanford Reservoir | 113 | 6.viii.2022 | Whitethroat | *Curruca communis* | Stanford Ringing Group |
| 1572 | | O.fringillina | | | | UNITED KINGDOM | ♀ | Leicestershire | ASmart | Lutterworth, North Kilworth, Hanglands | 151 | 16.viii.2022 | Robin | *Erithacus rubecula* | Stanford Ringing Group |
| 5294 | | O.fringillina | | | | UNITED KINGDOM | ♀ | Derbyshire | ASmart | Markham Vale | 97 | 29.viii.2022 | Goldcrest | *Regulus regulus* | Bryn Roberts |
| H930 | | O.fringillina | | | | UNITED KINGDOM | ♀ | East Sussex | BSmart | Icklesham | 10 | 16.viii.2022 | Whitethroat | *Curruca communis* | Rye Bay Ringing Group |
| H937 | | O.fringillina | | | | UNITED KINGDOM | ♀ | East Sussex | damaged | Icklesham | 10 | 12.ix.2022 | Whitethroat | *Curruca communis* | Rye Bay Ringing Group |
| H940 | | O.fringillina | | | | UNITED KINGDOM | ♀ | East Sussex | Hutson | Icklesham | 10 | 27.ix.2022 | Cetti's Warbler | *Cettia cetti* | Rye Bay Ringing Group |
| A30 | | O.fringillina | | | | UNITED KINGDOM | ♂ | Dumfries and Galloway | BSmart | RSPB Mersehead | 0 | 6.viii.2022 | Wren | *Troglodytes troglodytes* | ANON |
| 1073 | | O.fringillina | | | | UNITED KINGDOM | ♀ | Lincolnshire | BSmart | Owmby | 19 | 15.vii.2022 | Whitethroat | *Curruca communis* | Jenny Dunn |
| 1069 | | O.fringillina | | | | UNITED KINGDOM | ♀ | Lincolnshire | ASmart | Moorlands | 23 | 22.vii.2022 | Whitethroat | *Curruca communis* | Jenny Dunn |
| 7587 | | O.fringillina | | | | UNITED KINGDOM | ♀ | Devon | ASmart | Ilfracombe, West Down, Buttercombe Barton | 160 | 22.x.2022 | Goldcrest | *Regulus regulus* | Chris Dee |
| SB37 | | O.fringillina | | | | IRELAND | ♀ | Kerry | ASmart | Killarney National Park | 50 | 11.vii.2022 | Robin | *Erithacus rubecula* | Sam Bayley |
| SB83 | | O.fringillina | | | | IRELAND | ♀ | Cork | Hutson | Owenahincha, Little Island Wetland | 0 | 29.vii.2022 | Willow Warbler | *Phylloscopus trochilus* | Sam Bayley |
| SB68 | | O.fringillina | | | | IRELAND | ♀ | Cork | BSmart | Owenahincha, Little Island Wetland | 0 | 29.vii.2022 | Treecreeper | *Certhia familiaris* | Sam Bayley |
| SB41 | | O.fringillina | | | | IRELAND | ♂ | Kerry | BSmart | Killarney National Park | 50 | 16.viii.2022 | Goldcrest | *Regulus regulus* | Sam Bayley |
| SB30 | | O.fringillina | | | | IRELAND |  | Kerry |  | Killarney National Park | 50 | 25.xi.2022 | Firecrest | *Regulus ignicapilla* | Sam Bayley |
| H081 | | O.fringillina | | | | UNITED KINGDOM | ♀ | Dumfries and Galloway | ASmart | Toll | 58 | 18.ix.2022 | Robin | *Erithacus rubecula* | Tony Gibson |
| 5202 | | O.fringillina | | | | UNITED KINGDOM | ♀ | Derbyshire | ASmart | Moss Valley Meadows | 189 | 9.viii.2022 | Nuthatch | *Sitta europaea* | Ava Teasdale |
| H162 | | O.fringillina | | | | UNITED KINGDOM | ♀ | Cumbria | ASmart | Kirkbride | 10 | 28.vii.2022 | Dunnock | *Prunella modularis* | Frank Mawby |
| H164 | | O.fringillina | | | | UNITED KINGDOM |  | Cumbria | ASmart | Watchtree | 70 | 13.viii.2022 | Chaffinch | *Fringilla coelebs* | Frank Mawby |
| H165 | | O.fringillina | | | | UNITED KINGDOM |  | Cumbria | BSmart | Watchtree | 70 | 27.viii.2022 |  |  | Frank Mawby |
| H166 | | O.fringillina | | | | UNITED KINGDOM | ♀ | Cumbria | BSmart | Watchtree | 70 | 27.viii.2022 |  |  | Frank Mawby |
| 432 | | O.fringillina | | | | UNITED KINGDOM | ♀ | Norfolk | damaged | Burnham Deepdale | 10 | 4.ix.2022 | Robin | *Erithacus rubecula* | Denise Lamsdell |
| 439 | | O.fringillina | | | | UNITED KINGDOM | ♀ | Norfolk | BSmart | Little Snoring | 50 | 16.ix.2022 | Coal Tit | *Periparus ater* | Denise Lamsdell |
| H121 | | O.fringillina | | | | UNITED KINGDOM | ♂ | Skye | Hutson | Varagill | 60 | 4.ix.2022 |  |  | Jonathan Jones |
| H122 | | O.fringillina | | | | UNITED KINGDOM | ♂ | Skye | BSmart | Varagill | 60 | 4.ix.2022 |  |  | Jonathan Jones |
| H123 | | O.fringillina | | | | UNITED KINGDOM | ♀ | Skye | ASmart | Varagill | 60 | 4.ix.2022 |  |  | Jonathan Jones |
| H124 | | O.fringillina | | | | UNITED KINGDOM | ♂ | Skye | BSmart | Varagill | 60 | 4.ix.2022 |  |  | Jonathan Jones |
| H125 | | O.fringillina | | | | UNITED KINGDOM | ♂ | Skye | Hutson | Varagill | 60 | 8.ix.2022 | Goldcrest | *Regulus regulus* | Jonathan Jones |
| H126 | | O.fringillina | | | | UNITED KINGDOM | ♀ | Skye | BSmart | Portree | 40 | 15.ix.2022 | Chaffinch | *Fringilla coelebs* | Jonathan Jones |
| H127 | | O.fringillina | | | | UNITED KINGDOM | ♀ | Skye | BSmart | Portree | 40 | 15.ix.2022 | Chaffinch | *Fringilla coelebs* | Jonathan Jones |
| H128 | | O.fringillina | | | | UNITED KINGDOM | ♂ | Skye | BSmart | Portree | 40 | 15.ix.2022 | Siskin | *Spinus spinus* | Jonathan Jones |
| H129 | | O.fringillina | | | | UNITED KINGDOM | ♀ | Skye | BSmart | Portree | 40 | 16.ix.2022 | Siskin | *Spinus spinus* | Jonathan Jones |
| H130 | | O.fringillina | | | | UNITED KINGDOM | ♀ | Skye | ASmart | Portree | 40 | 17.ix.2022 | Chaffinch | *Fringilla coelebs* | Jonathan Jones |
| H131 | | O.fringillina | | | | UNITED KINGDOM | ♀ | Skye | BSmart | Portree | 40 | 17.ix.2022 | Siskin | *Spinus spinus* | Jonathan Jones |
| H132 | | O.fringillina | | | | UNITED KINGDOM | ♀ | Skye | damaged | Portree | 40 | 17.ix.2022 | Siskin | *Spinus spinus* | Jonathan Jones |
| H133 | | O.fringillina | | | | UNITED KINGDOM | ♀ | Skye | BSmart | Portree | 40 | 18.ix.2022 | Siskin | *Spinus spinus* | Jonathan Jones |
| H134 | | O.fringillina | | | | UNITED KINGDOM | ♂ | Skye | ASmart | Varagill | 60 | 19.ix.2022 | Long-tailed Tit | *Aegithalos caudatus* | Jonathan Jones |
| H135 | | O.fringillina | | | | UNITED KINGDOM | ♂ | Skye | BSmart | Edinbane | 5 | 24.ix.2022 | Great Tit | *Parus major* | Jonathan Jones |
| H136 | | O.fringillina | | | | UNITED KINGDOM | ♀ | Skye | ASmart | Portree | 40 | 24.ix.2022 | Siskin | *Spinus spinus* | Jonathan Jones |
| H137 | | O.fringillina | | | | UNITED KINGDOM | ♀ | Skye | BSmart | Varagill | 60 | 8.x.2022 | Goldcrest | *Regulus regulus* | Jonathan Jones |
| H139 | | O.fringillina | | | | UNITED KINGDOM | ♀ | Skye | ASmart | Varagill | 60 | 8.x.2022 | Goldcrest | *Regulus regulus* | Jonathan Jones |
| H140 | | O.fringillina | | | | UNITED KINGDOM | ♀ | Skye | ASmart | Varagill | 60 | 8.x.2022 | Goldcrest | *Regulus regulus* | Jonathan Jones |
| 7385 | | O.fringillina | | | | UNITED KINGDOM | ♀ | Carmarthenshire | BSmart | Bancyffordd | 205 |  |  |  | A. Turner |
| 7400A | | O.fringillina | | | | UNITED KINGDOM | ♂ | Carmarthenshire | ASmart | Bancyffordd | 205 | 18.vii.2022 | Dunnock | *Prunella modularis* | A. Turner |
| 7400B | | O.fringillina | | | | UNITED KINGDOM | ♀ | Carmarthenshire | BSmart | Bancyffordd | 205 | 18.vii.2022 | Dunnock | *Prunella modularis* | A. Turner |
| H841 | | O.fringillina | | | | UNITED KINGDOM | ♀ | Carmarthenshire | Hutson | Bancyffordd | 205 | 22.ix.2022 | Greenfinch | *Chloris chloris* | A. Turner |
| H645 | | O.fringillina | | | | UNITED KINGDOM | ♂ | Carmarthenshire |  | Bancyffordd | 205 | 18.vii.2022 | Dunnock | *Prunella modularis* | A. Turner |
| H650 | | O.fringillina | | | | UNITED KINGDOM | ♀ | Carmarthenshire | ASmart | Bancyffordd | 205 | 22.vii.2022 | Wren | *Troglodytes troglodytes* | A. Turner |
| H651 | | O.fringillina | | | | UNITED KINGDOM | ♂ | Carmarthenshire | ASmart | Bancyffordd | 205 | 22.vii.2022 |  |  | A. Turner |
| H652 | | O.fringillina | | | | UNITED KINGDOM | ♂ | Carmarthenshire | BSmart | Bancyffordd | 205 | 22.vii.2022 |  |  | A. Turner |
| H660 | | O.fringillina | | | | UNITED KINGDOM | ♂ | Carmarthenshire | BSmart | Bancyffordd | 205 | 26.viii.2022 | Robin | *Erithacus rubecula* | A. Turner |
| 22NW02 | | O.fringillina | | | | UNITED KINGDOM | ♀ | Devon | Hutson | Slapton Ley | 4 | 3.ix.2022 | Reed Warbler | *Acrocephalus scirpaceus* | Nik Ward |
| 22NW03 | | O.fringillina | | | | UNITED KINGDOM | ♀ | Devon | ASmart | Slapton Ley | 4 | 23.vii.2022 | Reed Warbler | *Acrocephalus scirpaceus* | Nik Ward |
| 22NW04 | | O.fringillina | | | | UNITED KINGDOM |  | Devon | BSmart | Slapton Ley | 4 | 16.vii.2022 | Whitethroat | *Curruca communis* | Nik Ward |
| 1581 | | O.fringillina | | | | UNITED KINGDOM | ♀ | Isle of Wight | damaged | Bembridge | 16 | 3.x.2022 | Goldfinch | *Carduelis carduelis* | Isle of Wight Ringing Group |
| 1888 | | O.fringillina | | | | UNITED KINGDOM | ♀ | Isle of Wight | damaged | Bembridge | 16 | 7.x.2022 | Goldfinch | *Carduelis carduelis* | Isle of Wight Ringing Group |
| 1885 | | O.fringillina | | | | UNITED KINGDOM | ♀ | Isle of Wight | BSmart | Bembridge | 16 | 27.x.2022 | Goldfinch | *Carduelis carduelis* | Isle of Wight Ringing Group |
| 7122 | | O.fringillina | | | | UNITED KINGDOM | ♀ | Shropshire |  | Brownheath | 90 | 18.ix.2022 | Reed Bunting | *Emberiza schoeniclus* | Martin George |
| 4323 | | O.fringillina | | | | UNITED KINGDOM | ♂ | Surrey | ASmart | Leith Hill, Duke's Warren | 258 | 1.viii.2022 | Robin | *Erithacus rubecula* | Paul Stevenson |
| 1449 | | O.fringillina | | | | UNITED KINGDOM | ♀ | Pembrokeshire | ASmart | Skokholm Island | 35 | 17.vi.2022 | Wheatear | *Oenanthe oenanthe* | Skokholm Bird Observatory |
| 1451 | | O.fringillina | | | | UNITED KINGDOM | ♀ | Pembrokeshire | ASmart | Skokholm Island | 35 | 12.vii.2022 | Sedge Warbler | *Acrocephalus schoenobaenus* | Skokholm Bird Observatory |
| H311 | | O.fringillina | | | | UNITED KINGDOM | ♀ | Devon | BSmart | Bridford | 244 | 8.x.2022 | Chiffchaff | *Phylloscopus collybita* | Samuel Gray |
| H302 | | O.fringillina | | | | UNITED KINGDOM | ♀ | Devon | ASmart | Bridford | 244 | 22.x.2022 | Robin | *Erithacus rubecula* | Samuel Gray |
| H305 | | O.fringillina | | | | UNITED KINGDOM | ♀ | Devon | BSmart | Bridford | 244 | 14.xi.2022 | Blue Tit | *Cyanistes caeruleus* | Samuel Gray |
| 4958 | | O.fringillina | | | | UNITED KINGDOM | ♀ | Rutland | BSmart | Rutland Water Nature Reserve | 85 | 13.vii.2022 | Goldcrest | *Regulus regulus* | Luke Nelson |
| B | | O.fringillina | | | | UNITED KINGDOM | ♂ | Rutland | BSmart | Rutland Water Nature Reserve | 85 | 25.viii.2022 | Blackcap | *Sylvia atricapilla* | Luke Nelson |
| A | | O.fringillina | | | | UNITED KINGDOM |  | Rutland | ASmart | Rutland Water Nature Reserve | 84 | 26.viii.2022 | Sedge Warbler | *Acrocephalus schoenobaenus* | Luke Nelson |
| 4128 | | O.fringillina | | | | UNITED KINGDOM | ♂ | Pembrokeshire | ASmart | Teifi | 0 | 31.viii.2022 | Goldcrest | *Regulus regulus* | Wendy James |
| 4129 | | O.fringillina | | | | UNITED KINGDOM | ♂ | Pembrokeshire | Hutson | Teifi Marshes, Pentood Marsh | 0 | 3.x.2022 | Blue Tit | *Cyanistes caeruleus* | Wendy James |
| RP01 | | O.fringillina | | | | UNITED KINGDOM | ♀ | Devon | ASmart | Exeter, Marsh Green | 70 | 5.xii.2022 | Blue Tit | *Cyanistes caeruleus* | Robin Pearson |
| 3103 | | O.fringillina | | | | UNITED KINGDOM | ♀ | Somerset | ASmart | Portishead, Gordano Valley | 7 | 15.ix.2022 | Wren | *Troglodytes troglodytes* | Gordano Valley RG |
| 2115 | | O.fringillina | | | | UNITED KINGDOM | ♂ | Gwynedd | Hutson | Rhostryfan | 150 | 17.vii.2022 | Willow Warbler | *Phylloscopus trochilus* | Adrienne Stratford |
| 2116 | | O.fringillina | | | | UNITED KINGDOM | ♂ | Gwynedd | Hutson | Rhostryfan | 150 | 21.vii.2022 | Robin | *Erithacus rubecula* | Adrienne Stratford |
| 2117 | | O.fringillina | | | | UNITED KINGDOM | ♀ | Gwynedd | Hutson | Rhostryfan | 150 | 27.vii.2022 | Whitethroat | *Curruca communis* | Adrienne Stratford |
| 2120 | | O.fringillina | | | | UNITED KINGDOM | ♂ | Gwynedd | Hutson | Rhostryfan | 150 | 5.viii.2022 | Goldcrest | *Regulus regulus* | Adrienne Stratford |
| H802 | | O.fringillina | | | | UNITED KINGDOM | ♀ | Gwynedd | Hutson | Rhostryfan | 150 | 25.viii.2002 | Blackcap | *Sylvia atricapilla* | Adrienne Stratford |
| H804 | | O.fringillina | | | | UNITED KINGDOM | ♂ | Gwynedd | Hutson | Rhostryfan | 150 | 9.ix.2022 | Chiffchaff | *Phylloscopus collybita* | Adrienne Stratford |
| H805 | | O.fringillina | | | | UNITED KINGDOM | ♀ | Gwynedd | Hutson | Rhostryfan | 150 | 14.ix.2022 | Meadow Pipit | *Anthus pratensis* | Adrienne Stratford |
| H806 | | O.fringillina | | | | UNITED KINGDOM | ♀ | Gwynedd | damaged | Rhostryfan | 150 | 8.x.2022 | Treecreeper | *Certhia familiaris* | Adrienne Stratford |
| 2266 | | O.fringillina | | | | UNITED KINGDOM | ♂ | Norfolk | BSmart | Holme, Holme, Holme Bird Observatory | 2 | 14.vi.2022 | Whitethroat | *Curruca communis* | Norfolk Ornithologists' Association |
| 2265 | | O.fringillina | | | | UNITED KINGDOM | ♀ | Norfolk | Hutson | Holme, Holme, Holme Bird Observatory | 2 | 23.vii.2022 | Whitethroat | *Curruca communis* | Norfolk Ornithologists' Association |
| 2267 | | O.fringillina | | | | UNITED KINGDOM |  | Norfolk | damaged | Holme, Holme, Holme Bird Observatory | 2 | 29.ix.2022 | Blue Tit | *Cyanistes caeruleus* | Norfolk Ornithologists' Association |
| FF194 | | O.fringillina | | | | UNITED KINGDOM | ♂ | Somerset | BSmart | Minehead, Bratton | 64 | 7.vii.2023 | House Sparrow | *Passer domesticus* | Denise Wawman |
| FF213 | | O.fringillina | | | | UNITED KINGDOM | ♂ | Somerset | BSmart | Minehead, Bratton | 64 | 15.vii.2023 | Robin | *Erithacus rubecula* | Denise Wawman |
| FF215 | | O.fringillina | | | | UNITED KINGDOM | ♀ | Somerset | ASmart | Minehead, Bratton | 64 | 18.vii.2023 |  |  | Denise Wawman |
| FF216 | | O.fringillina | | | | UNITED KINGDOM | ♂ | Somerset | BSmart | Minehead, Bratton | 64 | 18.vii.2023 | Dunnock | *Prunella modularis* | Denise Wawman |
| FF217 | | O.fringillina | | | | UNITED KINGDOM | ♀ | Somerset | Hutson | Minehead, Bratton | 64 | 18.vii.2023 | Dunnock | *Prunella modularis* | Denise Wawman |
| FF218 | | O.fringillina | | | | UNITED KINGDOM | ♀ | Somerset | ASmart | Minehead, Bratton | 64 | 18.vii.2023 | House Sparrow | *Passer domesticus* | Denise Wawman |
| FF225 | | O.fringillina | | | | UNITED KINGDOM | ♂ | Somerset | ASmart | Minehead, Bratton | 64 | 18.vii.2023 | Blue Tit | *Cyanistes caeruleus* | Denise Wawman |
| FF232 | | O.fringillina | | | | UNITED KINGDOM | ♂ | Somerset | ASmart | Minehead, Bratton | 64 | 20.vii.2023 | Dunnock | *Prunella modularis* | Denise Wawman |
| FF233 | | O.fringillina | | | | UNITED KINGDOM | ♂ | Somerset | Hutson | Minehead, Bratton | 64 | 20.vii.2023 | Blue Tit | *Cyanistes caeruleus* | Denise Wawman |
| H306 | | O.fringillina | | | | UNITED KINGDOM | ♀ | Devon | ASmart | Bridford | 244 | 23.vi.2023 | Blackcap | *Sylvia atricapilla* | Samuel Gray |
| FF240 | | O.fringillina | | | | UNITED KINGDOM | ♀ | Somerset |  | Minehead, Bratton | 64 | 25.vii.2023 | Robin | *Erithacus rubecula* | Denise Wawman |
| 7976 | | O.fringillina | | | | UNITED KINGDOM | ♂ | Inverness-shire | BSmart | Carse of Ardersier | 11 | 14.vii.2023 | Willow Warbler | *Phylloscopus trochilus* | Hugh Insley |
| 7938 | | O.fringillina | | | | UNITED KINGDOM | ♂ | Inverness-shire | BSmart | Carse of Ardersier | 11 | 14.vii.2023 | Willow Warbler | *Phylloscopus trochilus* | Hugh Insley |
| H147A | | O.fringillina | | | | UNITED KINGDOM | ♀ | Inverness-shire | BSmart | Inverness, Drummond | 29 | 15.vii.2023 | Dunnock | *Prunella modularis* | Hugh Insley |
| H147B | | O.fringillina | | | | UNITED KINGDOM | ♀ | Inverness-shire | BSmart | Inverness, Drummond | 29 | 15.vii.2023 | Dunnock | *Prunella modularis* | Hugh Insley |
| H154 | | O.fringillina | | | | UNITED KINGDOM | ♂ | Inverness-shire | BSmart | Inverness, Drummond | 29 | 15.vii.2023 | Dunnock | *Prunella modularis* | Hugh Insley |
| H152 | | O.fringillina | | | | UNITED KINGDOM | ♀ | Inverness-shire | BSmart | Inverness, Drummond | 29 | 15.vii.2023 | Dunnock | *Prunella modularis* | Hugh Insley |
| H155 | | O.fringillina | | | | UNITED KINGDOM | ♀ | Inverness-shire | BSmart | Inverness, Drummond | 29 | 15.vii.2023 | Siskin | *Spinus spinus* | Hugh Insley |
| H151 | | O.fringillina | | | | UNITED KINGDOM | ♂ | Inverness-shire | BSmart | Carse of Ardersier | 11 | 17.vii.2023 | Willow Warbler | *Phylloscopus trochilus* | Hugh Insley |
| H148 | | O.fringillina | | | | UNITED KINGDOM | ♂ | Inverness-shire | BSmart | Carse of Ardersier | 11 | 19.vii.2023 | Willow Warbler | *Phylloscopus trochilus* | Hugh Insley |
| H149 | | O.fringillina | | | | UNITED KINGDOM | ♂ | Inverness-shire | BSmart | Carse of Ardersier | 11 | 19.vii.2023 | Robin | *Erithacus rubecula* | Hugh Insley |
| H159 | | O.fringillina | | | | UNITED KINGDOM | ♂ | Inverness-shire | BSmart | Carse of Ardersier | 11 | 19.vii.2023 | Willow Warbler | *Phylloscopus trochilus* | Hugh Insley |
| H142 | | O.fringillina | | | | UNITED KINGDOM | ♀ | Inverness-shire | BSmart | Carse of Ardersier | 11 | 19.vii.2023 | Song Thrush | *Turdus philomelos* | Hugh Insley |
| H157 | | O.fringillina | | | | UNITED KINGDOM | ♂ | Inverness-shire | BSmart | Inverness, Drummond | 29 | 20.vii.2023 | Siskin | *Spinus spinus* | Hugh Insley |
| X395 | | O.fringillina | | | | UNITED KINGDOM | ♀ | Inverness-shire | BSmart | Carse of Ardersier | 11 | 21.vii.2023 | Blackcap | *Sylvia atricapilla* | Hugh Insley |
| FF421 | | O.fringillina | | | | UNITED KINGDOM | ♂ | Somerset | BSmart | Minehead, Bratton | 64 | 26.vii.2023 | Dunnock | *Prunella modularis* | Denise Wawman |
| A50 | | O.fringillina | | | | UNITED KINGDOM | ♀ | Norfolk | ASmart | Anmer, Crow Hill Cottage | 70 | 21.vii.2023 | Tree Sparrow | *Passer montanus* | Denise Lamsdell |
| FF242 | | O.fringillina | | | | UNITED KINGDOM | ♀ | Somerset | ASmart | Minehead, Bratton | 64 | 27.vii.2023 | Blue Tit | *Cyanistes caeruleus* | Denise Wawman |
| X278 | | O.fringillina | | | | UNITED KINGDOM | ♂ | Northamptonshire | BSmart | Stanford Reservoir | 113 | 26.vii.2023 | Chiffchaff | *Phylloscopus collybita* | Stanford Ringing Group |
| X280 | | O.fringillina | | | | UNITED KINGDOM | ♂ | Northamptonshire | ASmart | Corby | 90 | 28.vii.2023 | Dunnock | *Prunella modularis* | Adam Homer |
| FF246 | | O.fringillina | | | | UNITED KINGDOM | ♀ | Somerset | BSmart | Minehead, Bratton | 64 | 9.viii.2023 | Siskin | *Spinus spinus* | Denise Wawman |
| FF247 | | O.fringillina | | | | UNITED KINGDOM | ♂ | Somerset | ASmart | Minehead, Bratton | 64 | 11.viii.2023 | Robin | *Erithacus rubecula* | Denise Wawman |
| FF248 | | O.fringillina | | | | UNITED KINGDOM | ♂ | Somerset | BSmart | Minehead, Bratton | 64 | 11.viii.2023 | Robin | *Erithacus rubecula* | Denise Wawman |
| FF249 | | O.fringillina | | | | UNITED KINGDOM | ♀ | Somerset | BSmart | Minehead, Bratton | 64 | 11.viii.2023 | Robin | *Erithacus rubecula* | Denise Wawman |
| FF250 | | O.fringillina | | | | UNITED KINGDOM | ♂ | Somerset | ASmart | Minehead, Bratton | 64 | 11.viii.2023 | Robin | *Erithacus rubecula* | Denise Wawman |
| FF251 | | O.fringillina | | | | UNITED KINGDOM | ♂ | Somerset | BSmart | Minehead, Bratton | 64 | 16.viii.2023 | Robin | *Erithacus rubecula* | Denise Wawman |
| FF252 | | O.fringillina | | | | UNITED KINGDOM | ♀ | Somerset | ASmart | Minehead, Bratton | 64 | 16.viii.2023 | Robin | *Erithacus rubecula* | Denise Wawman |
| FF253 | | O.fringillina | | | | UNITED KINGDOM | ♀ | Somerset | BSmart | Minehead, Bratton | 64 | 16.viii.2023 | Robin | *Erithacus rubecula* | Denise Wawman |
| FF254 | | O.fringillina | | | | UNITED KINGDOM | ♂ | Somerset | BSmart | Minehead, Bratton | 64 | 16.viii.2023 | Robin | *Erithacus rubecula* | Denise Wawman |
| FF256 | | O.fringillina | | | | UNITED KINGDOM | ♀ | Somerset | BSmart | Minehead, Bratton | 64 | 16.viii.2023 | Robin | *Erithacus rubecula* | Denise Wawman |
| X467 | | O.fringillina | | | | UNITED KINGDOM | ♀ | Inverness-shire | ASmart | Carse of Ardersier | 11 | 31.vii.2023 | Blue Tit | *Cyanistes caeruleus* | Hugh Insley |
| X462 | | O.fringillina | | | | UNITED KINGDOM | ♂ | Inverness-shire | BSmart | Carse of Ardersier | 11 | 31.vii.2023 | Robin | *Erithacus rubecula* | Hugh Insley |
| X461 | | O.fringillina | | | | UNITED KINGDOM | ♀ | Inverness-shire | ASmart | Inverness, Drummond | 29 | 31.vii.2023 | Dunnock | *Prunella modularis* | Hugh Insley |
| H146 | | O.fringillina | | | | UNITED KINGDOM | ♀ | Inverness-shire | BSmart | Carse of Ardersier | 11 | 1.viii.2023 | Goldcrest | *Regulus regulus* | Hugh Insley |
| X464 | | O.fringillina | | | | UNITED KINGDOM | ♀ | Inverness-shire | BSmart | Inverness, Drummond | 29 | 2.viii.2023 | Chaffinch | *Fringilla coelebs* | Hugh Insley |
| X468 | | O.fringillina | | | | UNITED KINGDOM | ♂ | Inverness-shire | BSmart | Inverness, Drummond | 29 | 2.viii.2023 | Siskin | *Spinus spinus* | Hugh Insley |
| H158 | | O.fringillina | | | | UNITED KINGDOM | ♂ | Inverness-shire | BSmart | Carse of Ardersier | 11 | 3.viii.2023 | Dunnock | *Prunella modularis* | Hugh Insley |
| X459 | | O.fringillina | | | | UNITED KINGDOM | ♂ | Inverness-shire | BSmart | Carse of Ardersier | 11 | 3.viii.2023 | Blackcap | *Sylvia atricapilla* | Hugh Insley |
| X455 | | O.fringillina | | | | UNITED KINGDOM | ♀ | Inverness-shire | BSmart | Inverness, Drummond | 29 | 5.viii.2023 | Chaffinch | *Fringilla coelebs* | Hugh Insley |
| X456 | | O.fringillina | | | | UNITED KINGDOM | ♂ | Inverness-shire | BSmart | Inverness, Drummond | 29 | 5.viii.2023 | Chaffinch | *Fringilla coelebs* | Hugh Insley |
| X470 | | O.fringillina | | | | UNITED KINGDOM | ♂ | Inverness-shire | BSmart | Inverness, Drummond | 29 | 5.viii.2023 | Chaffinch | *Fringilla coelebs* | Hugh Insley |
| X458 | | O.fringillina | | | | UNITED KINGDOM | ♂ | Inverness-shire | BSmart | Inverness, Drummond | 29 | 5.viii.2023 | Chaffinch | *Fringilla coelebs* | Hugh Insley |
| X453 | | O.fringillina | | | | UNITED KINGDOM | ♂ | Inverness-shire | BSmart | Carse of Ardersier | 11 | 7.viii.2023 | Goldcrest | *Regulus regulus* | Hugh Insley |
| X460 | | O.fringillina | | | | UNITED KINGDOM | ♀ | Inverness-shire | damaged | Carse of Ardersier | 11 | 7.viii.2023 | Willow Warbler | *Phylloscopus trochilus* | Hugh Insley |
| X451 | | O.fringillina | | | | UNITED KINGDOM | ♀ | Inverness-shire | ASmart | Carse of Ardersier | 11 | 10.viii.2023 | Blackbird | *Turdus merula* | Hugh Insley |
| X452 | | O.fringillina | | | | UNITED KINGDOM | ♂ | Inverness-shire | Hutson | Carse of Ardersier | 11 | 13.viii.2023 | Goldcrest | *Regulus regulus* | Hugh Insley |
| X454 | | O.fringillina | | | | UNITED KINGDOM | ♀ | Inverness-shire | BSmart | Carse of Ardersier | 11 | 13.viii.2023 | Robin | *Erithacus rubecula* | Hugh Insley |
| X465 | | O.fringillina | | | | UNITED KINGDOM | ♂ | Inverness-shire | ASmart | Carse of Ardersier | 11 | 13.viii.2023 | Robin | *Erithacus rubecula* | Hugh Insley |
| X466 | | O.fringillina | | | | UNITED KINGDOM | ♀ | Inverness-shire | damaged | Carse of Ardersier | 11 | 14.viii.2023 | Goldcrest | *Regulus regulus* | Hugh Insley |
| X706 | | O.fringillina | | | | UNITED KINGDOM | ♀ | Inverness-shire | ASmart | Inverness, Drummond | 29 | 16.viii.2023 | Blue Tit | *Cyanistes caeruleus* | Hugh Insley |
| H255 | | O.fringillina | | | | UNITED KINGDOM | ♂ | Skye | BSmart | Portree | 40 | 19.vii.2023 | Blue Tit | *Cyanistes caeruleus* | Jonathan Jones |
| H284 | | O.fringillina | | | | UNITED KINGDOM | ♀ | Skye |  | Portree | 40 | 3.viii.2023 | Chaffinch | *Fringilla coelebs* | Jonathan Jones |
| H286 | | O.fringillina | | | | UNITED KINGDOM | ♂ | Skye | BSmart | Portree | 40 | 25.vii.2023 | House Sparrow | *Passer domesticus* | Jonathan Jones |
| H294 | | O.fringillina | | | | UNITED KINGDOM | ♂ | Skye | Hutson | Portree | 40 | 14.viii.2023 | Chaffinch | *Fringilla coelebs* | Jonathan Jones |
| H295 | | O.fringillina | | | | UNITED KINGDOM | ♂ | Skye | BSmart | Portree | 40 | 15.viii.2023 | Chaffinch | *Fringilla coelebs* | Jonathan Jones |
| H297 | | O.fringillina | | | | UNITED KINGDOM | ♀ | Skye | BSmart | Portree | 40 | 15.viii.2023 | Blue Tit | *Cyanistes caeruleus* | Jonathan Jones |
| H299 | | O.fringillina | | | | UNITED KINGDOM | ♀ | Skye | BSmart | Varagill | 60 | 17.viii.2023 | Robin | *Erithacus rubecula* | Jonathan Jones |
| H300 | | O.fringillina | | | | UNITED KINGDOM | ♀ | Skye | BSmart | Varagill | 60 | 17.viii.2023 | Robin | *Erithacus rubecula* | Jonathan Jones |
| FF257 | | O.fringillina | | | | UNITED KINGDOM | ♀ | Somerset | BSmart | Minehead, Bratton | 64 | 20.viii.2023 | House Sparrow | *Passer domesticus* | Denise Wawman |
| FF258 | | O.fringillina | | | | UNITED KINGDOM | ♀ | Somerset | BSmart | Minehead, Bratton | 64 | 20.viii.2023 | Goldfinch | *Carduelis carduelis* | Denise Wawman |
| FF260 | | O.fringillina | | | | UNITED KINGDOM | ♀ | Somerset | Hutson | Withiel Florey | 265 | 21.viii.2023 | Great Tit | *Parus major* | Denise Wawman |
| FF265 | | O.fringillina | | | | UNITED KINGDOM | ♀ | Somerset | BSmart | Minehead, Bratton | 64 | 25.viii.2023 |  |  | Denise Wawman |
| FF261 | | O.fringillina | | | | UNITED KINGDOM | ♀ | Somerset | ASmart | Minehead, Bratton | 64 | 25.viii.2023 | Dunnock | *Prunella modularis* | Denise Wawman |
| FF262 | | O.fringillina | | | | UNITED KINGDOM | ♀ | Somerset | BSmart | Minehead, Bratton | 64 | 25.viii.2023 | Dunnock | *Prunella modularis* | Denise Wawman |
| FF263 | | O.fringillina | | | | UNITED KINGDOM | ♀ | Somerset | BSmart | Minehead, Bratton | 64 | 25.viii.2023 | Dunnock | *Prunella modularis* | Denise Wawman |
| FF264 | | O.fringillina | | | | UNITED KINGDOM | ♂ | Somerset | BSmart | Minehead, Bratton | 64 | 25.viii.2023 | Robin | *Erithacus rubecula* | Denise Wawman |
| FF266 | | O.fringillina | | | | UNITED KINGDOM | ♂ | Somerset | BSmart | Minehead, Bratton | 64 | 25.viii.2023 |  |  | Denise Wawman |
| X362 | | O.fringillina | | | | UNITED KINGDOM | ♀ | Devon | BSmart | Bridford | 244 | 21.vii.2023 | Robin | *Erithacus rubecula* | Samuel Gray |
| X369 | | O.fringillina | | | | UNITED KINGDOM | ♂ | Devon | BSmart | Bridford | 244 | 21.vii.2023 | Chiffchaff | *Phylloscopus collybita* | Samuel Gray |
| X365 | | O.fringillina | | | | UNITED KINGDOM | ♀ | Devon | ASmart | Bridford | 244 | 25.vii.2023 | Whitethroat | *Curruca communis* | Samuel Gray |
| X361 | | O.fringillina | | | | UNITED KINGDOM | ♀ | Devon | Hutson | Bridford | 244 | 25.vii.2023 | Blackcap | *Sylvia atricapilla* | Samuel Gray |
| X367 | | O.fringillina | | | | UNITED KINGDOM | ♀ | Devon | Hutson | Bridford | 244 | 26.vii.2023 | Linnet | *Linaria cannabina* | Samuel Gray |
| X368 | | O.fringillina | | | | UNITED KINGDOM | ♂ | Devon | Hutson | Bridford | 244 | 28.vii.2023 | Chiffchaff | *Phylloscopus collybita* | Samuel Gray |
| X355 | | O.fringillina | | | | UNITED KINGDOM | ♀ | Devon | BSmart | Bridford | 244 | 8.viii.2023 | Wren | *Troglodytes troglodytes* | Samuel Gray |
| X352 | | O.fringillina | | | | UNITED KINGDOM | ♂ | Devon | BSmart | Bridford | 244 | 10.viii.2023 | Robin | *Erithacus rubecula* | Samuel Gray |
| X360 | | O.fringillina | | | | UNITED KINGDOM | ♀ | Devon | BSmart | Bridford | 244 | 15.viii.2023 | Willow Warbler | *Phylloscopus trochilus* | Samuel Gray |
| X358 | | O.fringillina | | | | UNITED KINGDOM | ♀ | Devon | damaged | Bridford | 244 | 15.viii.2023 | Chiffchaff | *Phylloscopus collybita* | Samuel Gray |
| X357 | | O.fringillina | | | | UNITED KINGDOM | ♀ | Devon | BSmart | Bridford | 244 | 22.viii.2023 | Robin | *Erithacus rubecula* | Samuel Gray |
| FF268 | | O.fringillina | | | | UNITED KINGDOM | ♀ | Somerset | damaged | Minehead, Bratton | 64 | 7.ix.2023 | Blue Tit | *Cyanistes caeruleus* | Denise Wawman |
| X571 | | O.fringillina | | | | UNITED KINGDOM | ♀ | Devon | BSmart | Ilfracombe, West Down, Buttercombe Barton | 160 | 8.viii.2023 | Goldfinch | *Carduelis carduelis* | Chris Dee |
| X573 | | O.fringillina | | | | UNITED KINGDOM | ♀ | Devon | damaged | Ilfracombe, West Down, Buttercombe Barton | 160 | 1.ix.2023 | Goldfinch | *Carduelis carduelis* | Chris Dee |
| 7595 | | O.fringillina | | | | UNITED KINGDOM | ♀ | Devon | BSmart | Ilfracombe, West Down, Buttercombe Barton | 160 | 21.vii.2023 |  |  | Chris Dee |
| 7598 | | O.fringillina | | | | UNITED KINGDOM | ♀ | Devon | Hutson | Ilfracombe, West Down, Buttercombe Barton | 160 | 29.vii.2023 | Dunnock | *Prunella modularis* | Chris Dee |
| 3286 | | O.fringillina | | | | UNITED KINGDOM | ♀ | Kirkcudbrightshire | BSmart | Mossdale | 55 | 25.vii.2023 |  |  | Tony Gibson |
| FF269 | | O.fringillina | | | | UNITED KINGDOM | ♀ | Somerset | BSmart | Minehead, Bratton | 64 | 21.ix.2023 | Robin | *Erithacus rubecula* | Denise Wawman |
| FF270 | | O.fringillina | | | | UNITED KINGDOM | ♀ | Somerset | ASmart | Minehead, Bratton | 64 | 21.ix.2023 | Robin | *Erithacus rubecula* | Denise Wawman |
| H274 | | O.fringillina | | | | UNITED KINGDOM | ♀ | Inverness-shire | BSmart | Carse of Ardersier | 11 | 29.viii.2023 | Coal Tit | *Periparus ater* | Hugh Insley |
| H268 | | O.fringillina | | | | UNITED KINGDOM | ♀ | Inverness-shire | damaged | Carse of Ardersier | 11 | 31.viii.2023 | Sparrowhawk | *Accipiter nisus* | Hugh Insley |
| X601 | | O.fringillina | | | | UNITED KINGDOM | ♀ | Inverness-shire | BSmart | Carse of Ardersier | 11 | 15.ix.2023 | Goldcrest | *Regulus regulus* | Hugh Insley |
| H541 | | O.fringillina | | | | UNITED KINGDOM | ♂ | Skye | Hutson | Varagill | 60 | 17.viii.2023 | Goldcrest | *Regulus regulus* | Jonathan Jones |
| H542 | | O.fringillina | | | | UNITED KINGDOM | ♀ | Skye | Hutson/BSmart | Varagill | 60 | 17.viii.2023 | Goldcrest | *Regulus regulus* | Jonathan Jones |
| H543 | | O.fringillina | | | | UNITED KINGDOM | ♂ | Skye | BSmart | Varagill | 60 | 17.viii.2023 |  |  | Jonathan Jones |
| H544 | | O.fringillina | | | | UNITED KINGDOM | ♀ | Skye | ASmart | Varagill | 60 | 17.viii.2023 | Robin | *Erithacus rubecula* | Jonathan Jones |
| H545 | | O.fringillina | | | | UNITED KINGDOM | ♂ | Skye | BSmart | Varagill | 60 | 17.viii.2023 |  |  | Jonathan Jones |
| H546 | | O.fringillina | | | | UNITED KINGDOM | ♂ | Skye | BSmart | Varagill | 60 | 17.viii.2023 | Goldcrest | *Regulus regulus* | Jonathan Jones |
| H549 | | O.fringillina | | | | UNITED KINGDOM | ♀ | Skye | ASmart | Portree | 40 | 24.viii.2023 | Chaffinch | *Fringilla coelebs* | Jonathan Jones |
| H550 | | O.fringillina | | | | UNITED KINGDOM | ♀ | Ross and Cromarty |  | Avoch | 115 | 20.viii.2023 |  |  | Jonathan Jones |
| H551 | | O.fringillina | | | | UNITED KINGDOM | ♀ | Skye | BSmart | Portree | 40 | 24.viii.2023 | Chaffinch | *Fringilla coelebs* | Jonathan Jones |
| H552 | | O.fringillina | | | | UNITED KINGDOM | ♂ | Skye | Hutson | Portree | 40 | 24.viii.2023 | Chaffinch | *Fringilla coelebs* | Jonathan Jones |
| H553 | | O.fringillina | | | | UNITED KINGDOM | ♀ | Skye | BSmart | Portree | 40 | 24.viii.2023 | Chaffinch | *Fringilla coelebs* | Jonathan Jones |
| H554 | | O.fringillina | | | | UNITED KINGDOM | ♂ | Skye | BSmart | Portree | 40 | 24.viii.2023 | House Sparrow | *Passer domesticus* | Jonathan Jones |
| H555 | | O.fringillina | | | | UNITED KINGDOM | ♂ | Skye | Hutson | Portree | 40 | 24.viii.2023 | Chaffinch | *Fringilla coelebs* | Jonathan Jones |
| H556 | | O.fringillina | | | | UNITED KINGDOM | ♂ | Skye | Hutson | Varagill | 60 | 27.viii.2023 | Goldcrest | *Regulus regulus* | Jonathan Jones |
| H557 | | O.fringillina | | | | UNITED KINGDOM | ♀ | Skye | BSmart | Varagill | 60 | 27.viii.2023 | Great Tit | *Parus major* | Jonathan Jones |
| H558 | | O.fringillina | | | | UNITED KINGDOM | ♀ | Skye | BSmart | Varagill | 60 | 27.viii.2023 | Coal Tit | *Periparus ater* | Jonathan Jones |
| H560 | | O.fringillina | | | | UNITED KINGDOM | ♀ | Skye | BSmart | Portree | 40 | 27.viii.2023 | Chaffinch | *Fringilla coelebs* | Jonathan Jones |
| H981 | | O.fringillina | | | | UNITED KINGDOM | ♀ | Skye | BSmart | Portree | 40 | 28.viii.2023 | Dunnock | *Prunella modularis* | Jonathan Jones |
| H982 | | O.fringillina | | | | UNITED KINGDOM | ♀ | Skye | BSmart | Portree | 40 | 28.viii.2023 | Dunnock | *Prunella modularis* | Jonathan Jones |
| H983 | | O.fringillina | | | | UNITED KINGDOM | ♂ | Skye | BSmart | Portree | 40 | 29.viii.2023 | Chaffinch | *Fringilla coelebs* | Jonathan Jones |
| H984 | | O.fringillina | | | | UNITED KINGDOM | ♂ | Skye | Hutson | Portree | 40 | 30.viii.2023 | Chaffinch | *Fringilla coelebs* | Jonathan Jones |
| H985 | | O.fringillina | | | | UNITED KINGDOM | ♂ | Skye | BSmart | Portree | 40 | 30.viii.2023 |  |  | Jonathan Jones |
| H986 | | O.fringillina | | | | UNITED KINGDOM | ♀ | Skye | BSmart | Portree | 40 | 5.ix.2023 | Chaffinch | *Fringilla coelebs* | Jonathan Jones |
| H987 | | O.fringillina | | | | UNITED KINGDOM | ♀ | Skye | BSmart | Portree | 40 | 5.ix.2023 | Chaffinch | *Fringilla coelebs* | Jonathan Jones |
| H988 | | O.fringillina | | | | UNITED KINGDOM | ♀ | Skye | ASmart | Portree | 40 | 7.ix.2023 | Chaffinch | *Fringilla coelebs* | Jonathan Jones |
| 2419 | | O.fringillina | | | | UNITED KINGDOM | ♀ | Cheshire | ASmart | Hoylake | 6 | 21.vi.2023 | Robin | *Erithacus rubecula* | Jane Turner |
| X037 | | O.fringillina | | | | UNITED KINGDOM | ♀ | Cheshire | BSmart | Hoylake | 6 | 26.vii.2023 | Goldfinch | *Carduelis carduelis* | Jane Turner |
| X044 | | O.fringillina | | | | UNITED KINGDOM | ♀ | Cheshire | ASmart | Hoylake | 6 | 26.vii.2023 | Robin | *Erithacus rubecula* | Jane Turner |
| X045 | | O.fringillina | | | | UNITED KINGDOM | ♀ | Cheshire | Hutson/BSmart | Hoylake | 6 | 1.ix.2023 | Robin | *Erithacus rubecula* | Jane Turner |
| 5257 | | O.fringillina | | | | UNITED KINGDOM | ♀ | East Sussex | BSmart | Icklesham | 10 | 16.viii.2023 | Whitethroat | *Curruca communis* | Rye Bay Ringing Group |
| 5250 | | O.fringillina | | | | UNITED KINGDOM | ♂ | East Sussex | BSmart | Icklesham | 10 | ..2023 |  |  | Rye Bay Ringing Group |
| X783 | | O.fringillina | | | | UNITED KINGDOM | ♀ | East Sussex | BSmart | Icklesham | 10 | 19.viii.2023 | Whitethroat | *Curruca communis* | Rye Bay Ringing Group |
| X800 | | O.fringillina | | | | UNITED KINGDOM | ♀ | East Sussex | BSmart | Icklesham | 10 | 7.ix.2023 | Reed Warbler | *Acrocephalus scirpaceus* | Rye Bay Ringing Group |
| X791 | | O.fringillina | | | | UNITED KINGDOM | ♀ | East Sussex | BSmart | Icklesham | 10 | 14.ix.2023 |  |  | Rye Bay Ringing Group |
| X795 | | O.fringillina | | | | UNITED KINGDOM | ♀ | East Sussex | ASmart | Icklesham | 10 | 17.ix.2023 | Cetti's Warbler | *Cettia cetti* | Rye Bay Ringing Group |
| X780 | | O.fringillina | | | | UNITED KINGDOM | ♀ | East Sussex | BSmart | Icklesham | 10 | 28.ix.2023 | Chiffchaff | *Phylloscopus collybita* | Rye Bay Ringing Group |
| X764 | | O.fringillina | | | | UNITED KINGDOM | ♀ | East Sussex | ASmart | Icklesham | 10 | 19.x.2023 | Goldfinch | *Carduelis carduelis* | Rye Bay Ringing Group |
| X532 | | O.fringillina | | | | UNITED KINGDOM | ♂ | Devon | BSmart | Bridford | 244 | 22.viii.2023 | Chiffchaff | *Phylloscopus collybita* | Samuel Gray |
| X543 | | O.fringillina | | | | UNITED KINGDOM | ♀ | Devon | ASmart | Bridford | 244 | 23.viii.2023 | Willow Warbler | *Phylloscopus trochilus* | Samuel Gray |
| X541 | | O.fringillina | | | | UNITED KINGDOM | ♂ | Devon | Hutson | Bridford | 244 | 23.viii.2023 | Chiffchaff | *Phylloscopus collybita* | Samuel Gray |
| X533 | | O.fringillina | | | | UNITED KINGDOM | ♂ | Devon | BSmart | Bridford | 244 | 24.viii.2023 | Goldcrest | *Regulus regulus* | Samuel Gray |
| X526 | | O.fringillina | | | | UNITED KINGDOM | ♂ | Devon | BSmart | Bridford | 244 | 12.ix.2023 | Goldcrest | *Regulus regulus* | Samuel Gray |
| X550 | | O.fringillina | | | | UNITED KINGDOM | ♀ | Devon | BSmart | Bridford | 244 | 12.ix.2023 | Chiffchaff | *Phylloscopus collybita* | Samuel Gray |
| X548 | | O.fringillina | | | | UNITED KINGDOM | ♀ | Devon | BSmart | Bridford | 244 | 13.ix.2023 | Blackcap | *Sylvia atricapilla* | Samuel Gray |
| X535 | | O.fringillina | | | | UNITED KINGDOM | ♀ | Devon | BSmart | Bridford | 244 | 15.ix.2023 | Blackcap | *Sylvia atricapilla* | Samuel Gray |
| X527 | | O.fringillina | | | | UNITED KINGDOM | ♂ | Devon | BSmart | Bridford | 244 | 15.ix.2023 | Coal Tit | *Periparus ater* | Samuel Gray |
| X536 | | O.fringillina | | | | UNITED KINGDOM | ♀ | Devon | BSmart | Bridford | 244 | 25.ix.2023 | Chiffchaff | *Phylloscopus collybita* | Samuel Gray |
| X538 | | O.fringillina | | | | UNITED KINGDOM | ♂ | Devon | BSmart | Bridford | 244 | 1.x.2023 | Chiffchaff | *Phylloscopus collybita* | Samuel Gray |
| X545 | | O.fringillina | | | | UNITED KINGDOM | ♂ | Devon | Hutson | Bridford | 244 | 1.x.2023 | Long-tailed Tit | *Aegithalos caudatus* | Samuel Gray |
| X524 | | O.fringillina | | | | UNITED KINGDOM | ♂ | Devon | ASmart | Bridford | 244 | 6.x.2023 | Firecrest | *Regulus ignicapilla* | Samuel Gray |
| 701 | | O.fringillina | | | | UNITED KINGDOM | ♀ | Norfolk |  | Little Snoring | 55 | 1.xi.2023 | Blue Tit | *Cyanistes caeruleus* | Denise Lamsdell |
| 197 | | O.fringillina | | | | UNITED KINGDOM | ♀ | East Riding of Yorkshire | damaged | Flamborough | 45 | 28.vii.2023 | Whitethroat | *Curruca communis* | Andy Hood |
| 198 | | O.fringillina | | | | UNITED KINGDOM | ♂ | East Riding of Yorkshire | ASmart | Flamborough | 45 | 28.vii.2023 | Whitethroat | *Curruca communis* | Andy Hood |
| D05 | | O.fringillina | | | | UNITED KINGDOM | ♂ | East Riding of Yorkshire | Hutson | Flamborough | 45 | 15.viii.2023 | Robin | *Erithacus rubecula* | Andy Hood |
| D07 | | O.fringillina | | | | UNITED KINGDOM | ♀ | East Riding of Yorkshire | BSmart | Flamborough | 45 | 15.viii.2023 | Willow Warbler | *Phylloscopus trochilus* | Andy Hood |
| D08 | | O.fringillina | | | | UNITED KINGDOM | ♀ | East Riding of Yorkshire | ASmart | Flamborough | 45 | 21.viii.2023 | Tree Sparrow | *Passer montanus* | Andy Hood |
| 1464 | | O.fringillina | | | | UNITED KINGDOM | ♂ | Pembrokeshire | Hutson | Skokholm Island | 30 | 23.viii.2023 | Pied Wagtail | *Motacilla alba* | Skokholm Bird Observatory |
| 551 | | O.fringillina | | | | UNITED KINGDOM | ♀ | East Sussex | BSmart | Wivelsfield | 40 | 25.x.2023 | Blue Tit | *Cyanistes caeruleus* | Clare Buckle |
| H275 | | O.fringillina | | | | UNITED KINGDOM | ♀ | Inverness-shire | BSmart | Carse of Ardersier | 11 | 26.x.2023 | Chaffinch | *Fringilla coelebs* | Hugh Insley |
| X503 | | O.fringillina | | | | UNITED KINGDOM | ♀ | East Riding of Yorkshire | ASmart | Bempton Cliffs | 92 | 7.ix.2023 | Goldfinch | *Carduelis carduelis* | David Aitken |
| X519 | | O.fringillina | | | | UNITED KINGDOM | ♀ | East Riding of Yorkshire | ASmart | Bempton Cliffs | 92 | 7.ix.2023 | Goldfinch | *Carduelis carduelis* | David Aitken |
| X512 | | O.fringillina | | | | UNITED KINGDOM | ♀ | East Riding of Yorkshire | ASmart | Bempton Cliffs | 92 | 17.ix.2023 | Great Tit | *Parus major* | David Aitken |
| X471 | | O.fringillina | | | | UNITED KINGDOM | ♀ | Northamptonshire | ASmart | Stanford Reservoir | 113 | 29.vii.2023 | Reed Warbler | *Acrocephalus scirpaceus* | Stanford Ringing Group |
| X472 | | O.fringillina | | | | UNITED KINGDOM | ♀ | Northamptonshire | BSmart | Corby | 90 | 31.vii.2023 | Dunnock | *Prunella modularis* | Adam Homer |
| X475 | | O.fringillina | | | | UNITED KINGDOM | ♀ | Northamptonshire | BSmart | Stanford Reservoir | 113 | 12.viii.2023 | Whitethroat | *Curruca communis* | Stanford Ringing Group |
| X481 | | O.fringillina | | | | UNITED KINGDOM | ♀ | Northamptonshire | BSmart | Stanford Reservoir | 113 | 20.viii.2023 | Willow Warbler | *Phylloscopus trochilus* | Stanford Ringing Group |
| X482 | | O.fringillina | | | | UNITED KINGDOM | ♀ | Northamptonshire | BSmart | Stanford Reservoir | 113 | 25.viii.2023 | Whitethroat | *Curruca communis* | Stanford Ringing Group |
| X483 | | O.fringillina | | | | UNITED KINGDOM | ♂ | Northamptonshire | ASmart | Stanford Reservoir | 113 | 26.viii.2023 | Reed Warbler | *Acrocephalus scirpaceus* | Stanford Ringing Group |
| 1544 | | O.fringillina | | | | UNITED KINGDOM | ♀ | Huntingdonshire | BSmart | Woodwalton Fen | 2 | 31.viii.2023 | Sedge Warbler | *Acrocephalus schoenobaenus* | Stanford Ringing Group |
| X487 | | O.fringillina | | | | UNITED KINGDOM | ♀ | Northamptonshire | ASmart | Stanford Reservoir | 113 | 9.ix.2023 | Blackcap | *Sylvia atricapilla* | Stanford Ringing Group |
| X488 | | O.fringillina | | | | UNITED KINGDOM | ♀ | Northamptonshire | BSmart | Stanford Reservoir | 113 | 9.ix.2023 | Blackcap | *Sylvia atricapilla* | Stanford Ringing Group |
| 3664 | | O.fringillina | | | | UNITED KINGDOM | ♀ | Suffolk | ASmart | Languard Bird Observatory | 0 | 30.ix.2023 | Blue Tit | *Cyanistes caeruleus* | Languard Bird Observatory |
| 7693 | | O.fringillina | | | | UNITED KINGDOM | ♂ | Devon | BSmart | Buckfastleigh, Brook Manor, | 100 | 2.ix.2023 | Goldcrest | *Regulus regulus* | Ellie Ness & Robbie Phillips |
| 7466 | | O.fringillina | | | | UNITED KINGDOM | ♀ | Devon | BSmart | Buckfastleigh, Brook Manor, | 100 | 3.ix.2023 | Chiffchaff | *Phylloscopus collybita* | Ellie Ness & Robbie Phillips |
| 7470 | | O.fringillina | | | | UNITED KINGDOM | ♀ | Devon | BSmart | Buckfastleigh, Brook Manor, | 100 | 30.ix.2023 | Blue Tit | *Cyanistes caeruleus* | Ellie Ness & Robbie Phillips |
| 7472 | | O.fringillina | | | | UNITED KINGDOM | ♂ | Devon | BSmart | Buckfastleigh, Brook Manor, | 100 | 1.x.2023 | Goldcrest | *Regulus regulus* | Ellie Ness & Robbie Phillips |
| 7479 | | O.fringillina | | | | UNITED KINGDOM | ♀ | Devon | Hutson | Buckfastleigh, Brook Manor, | 100 | 1.x.2023 | Goldcrest | *Regulus regulus* | Ellie Ness & Robbie Phillips |
| H049 | | O.fringillina | | | | UNITED KINGDOM | ♀ | Hertfordshire |  | Benington | 105 | 8.x.2023 | Yellowhammer | *Emberiza citrinella* | Paul Roper |
| X057 | | O.fringillina | | | | UNITED KINGDOM | ♀ | Argyll | BSmart | Glen Euchar | 104 | 10.ix.2023 | Robin | *Erithacus rubecula* | Rob Lightfoot |
| 2010 | | O.fringillina | | | | UNITED KINGDOM | ♀ | Angus |  | Montrose, Wellington Park | 8 | 5.x.2023 | Goldfinch | *Carduelis carduelis* | Ben Herschell |
| X346 | | O.fringillina | | | | UNITED KINGDOM | ♀ | Northamptonshire | Hutson | Stanford Reservoir | 113 | 16.ix.2023 | Blackcap | *Sylvia atricapilla* | Stanford Ringing Group |
| H171 | | O.fringillina | | | | UNITED KINGDOM | ♀ | Cumbria | ASmart | Kirkbride | 13 | 10.vii.2023 | Blackbird | *Turdus merula* | Frank Mawby |
| 2124 | | O.fringillina | | | | UNITED KINGDOM | ♀ | Stirlingshire | BSmart | Dunblane, Kinbuck | 98 | 13.viii.2023 | Chaffinch | *Fringilla coelebs* | Liam Reid |
| H202 | | O.fringillina | | | | UNITED KINGDOM | ♀ | Northamptonshire | BSmart | Pitsford Reservoir | 88 | 31.viii.2023 | Whitethroat | *Curruca communis* | Northants Ringing Group |
| H689 | | O.fringillina | | | | UNITED KINGDOM | ♂ | East Riding of Yorkshire | Hutson | Flamborough, South Landing | 34 | 17.viii.2023 | Tree Sparrow | *Passer montanus* | Jo Hood |
| H682 | | O.fringillina | | | | UNITED KINGDOM | ♀ | East Riding of Yorkshire | BSmart | Flamborough, South Landing | 34 | 27.viii.2023 | Lesser Redpoll | *Acanthis cabaret* | Jo Hood |
| GRIEV1 | | O.fringillina | | | | UNITED KINGDOM | ♂ | Lanarkshire | BSmart | Biggar, Symington | 220 | .ix.2023 | Dunnock | *Prunella modularis* | David Grieve |
| 1110A | | O.fringillina | | | | UNITED KINGDOM | ♀ | Lincolnshire | ASmart | Nocton Fen, Wasps Nest | 10 | 31.vii.2023 | Linnet | *Linaria cannabina* | Carl Soulsbury |
| 1110B | | O.fringillina | | | | UNITED KINGDOM | ♀ | Lincolnshire | BSmart | Nocton Fen, Wasps Nest | 10 | 31.vii.2023 | Linnet | *Linaria cannabina* | Carl Soulsbury |
| H812 | | O.fringillina | | | | UNITED KINGDOM | ♂ | Gwynedd | Hutson | Rhostryfan | 150 | 29.vii.2023 | Chiffchaff | *Phylloscopus collybita* | Adrienne Stratford |
| H813 | | O.fringillina | | | | UNITED KINGDOM | ♀ | Gwynedd | BSmart | Rhostryfan | 150 | 7.viii.2023 | Blackcap | *Sylvia atricapilla* | Adrienne Stratford |
| 4860 | | O.fringillina | | | | UNITED KINGDOM | ♀ | Powys | BSmart | Brecon, Llangorse Lake | 160 | 28.vii.2023 | Reed Warbler | *Acrocephalus scirpaceus* | Llangorse Ringing Group |
| 4846 | | O.fringillina | | | | UNITED KINGDOM | ♀ | Powys | ASmart | Brecon, Llangorse Lake | 160 | 27.viii.2023 | Spotted Flycatcher | *Muscicapa striata* | Llangorse Ringing Group |
| 4848 | | O.fringillina | | | | UNITED KINGDOM | ♀ | Powys | BSmart | Brecon, Llangorse Lake | 160 | 14.ix.2023 | Blackcap | *Sylvia atricapilla* | Llangorse Ringing Group |
| 4849 | | O.fringillina | | | | UNITED KINGDOM | ♀ | Powys | BSmart | Brecon, Llangorse Lake | 160 | 6.x.2023 | Blue Tit | *Cyanistes caeruleus* | Llangorse Ringing Group |
| 4850 | | O.fringillina | | | | UNITED KINGDOM | ♀ | Powys | BSmart | Brecon, Llangorse Lake | 160 | 6.x.2023 | Goldcrest | *Regulus regulus* | Llangorse Ringing Group |
| 5129 | | O.fringillina | | | | UNITED KINGDOM | ♀ | Suffolk | damaged | Brandon | 25 | 2.xii.2022 | Blue Tit | *Cyanistes caeruleus* | Dr Greg Conway |
| 5138 | | O.fringillina | | | | UNITED KINGDOM | ♀ | Suffolk | damaged | Brandon | 25 | 20.xi.2022 | Blue Tit | *Cyanistes caeruleus* | Dr Greg Conway |
| H018 | | O.fringillina | | | | UNITED KINGDOM | ♀ | Suffolk | ASmart | Brandon | 25 | 5.viii.2023 | Blue Tit | *Cyanistes caeruleus* | Dr Greg Conway |
| SB26 | | O.fringillina | | | | IRELAND | ♂ | Cork | BSmart | Clonakilty, Clogheen Marsh | 5 | 25.vii.2023 | Wren | *Troglodytes troglodytes* | Sam Bayley |
| H989 | | O.fringillina | | | | UNITED KINGDOM | ♂ | Skye | BSmart | Portree | 40 | 17.x.2023 |  |  | Jonathan Jones |
| WWFF01 | | O.fringillina | | | | UNITED KINGDOM | ♀ | Berkshire | ASmart | Oxford, Wytham Woods |  | 15.i.2024 | Great Tit | *Parus major* | Kyu Min Huh & Keith Mc Mahon |
|  |  |  |  |  |  |  |  |  |  |  |  |  |  |  |  |
|  |  |  |  |  |  |  |  |  |  |  |  |  |  |  |  |
|  |  |  |  |  |  |  |  |  |  |  |  |  |  |  |  |
|  |  |  |  |  |  |  |  |  |  |  |  |  |  |  |  |
