# Supplementary material for: Clarifying the Taxonomy of the Finch Louse Fly Ornithomya Fringillina (Curtis) (Diptera: Hippoboscidae) – An Analysis of Morphotypes
Source: Acta Parasitol. 2025 Aug 8;70(4):175. doi: 10.1007/s11686-025-01113-z (PMC12334518; doi:10.1007/s11686-025-01113-z)

**Online Resource 2:**

Fig. S1: Photographs of the wings of the three *Ornithomya fringillina* morphotypes: a) Smart A; b) Smart B; c) Hutson. These photographs were taken using a Leica M165 C microscope with a Leica DFC490 camera mount at Oxford University Museum of Natural History. The flies were placed on their backs, between two microscope slides, with ethanol, to hold the wings in place. The microscope stage was backlit. The darker artifacts amongst the microtrichia are mainly caused by droplets of ethanol.


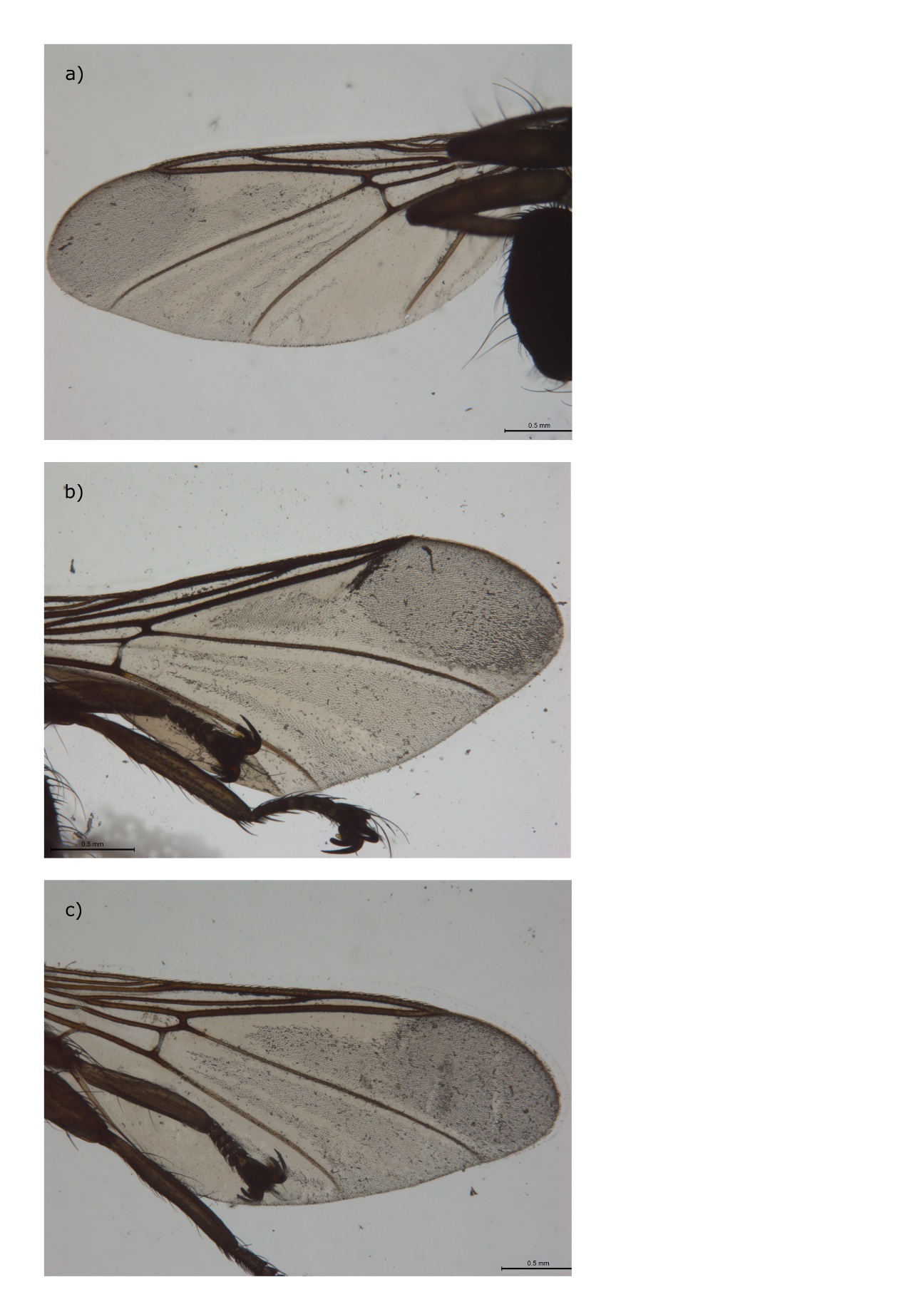

Supplement: Supplementary file 2 — Supplementary Material 2 [file 11686_2025_1113_MOESM2_ESM.docx]
