# Supplementary material for: Clarifying the Taxonomy of the Finch Louse Fly Ornithomya Fringillina (Curtis) (Diptera: Hippoboscidae) – An Analysis of Morphotypes
Source: Acta Parasitol. 2025 Aug 8;70(4):175. doi: 10.1007/s11686-025-01113-z (PMC12334518; doi:10.1007/s11686-025-01113-z)
Supplement: Supplementary file 3 — Supplementary Material 3 [file 11686_2025_1113_MOESM3_ESM.docx]

Table S2: A table showing the host species associations of the three *Ornithomya fringillina* morphotypes. The category mixed includes louse flies with wings of more than one morphotype. Flies were excluded if their wings were too damaged to categorise or if the data were missing. The host species are arranged in taxonomic order, following the British Ornithological Union (BOU) list.
