# Supplementary material for: Clarifying the Taxonomy of the Finch Louse Fly Ornithomya Fringillina (Curtis) (Diptera: Hippoboscidae) – An Analysis of Morphotypes
Source: Acta Parasitol. 2025 Aug 8;70(4):175. doi: 10.1007/s11686-025-01113-z (PMC12334518; doi:10.1007/s11686-025-01113-z)

**Online Resource 4:**

Table S3. Contributions of the variables to the principal components (loadings), with values over 0.5 highlighted in bold typeface, for the analysis of two morphotypes of *Ornithomya fringillina*

|  | **PC1** | **PC2** | **PC3** | **PC4** | **PC5** | **PC6** | **PC7** | **PC8** |
| --- | --- | --- | --- | --- | --- | --- | --- | --- |
| Fly wing | -0.19688 | 0.290685 | -0.61492 | -0.13575 | 0.026845 | -0.61596 | 0.313605 | -0.04136 |
| Scutellar bristles | 0.038708 | -0.17843 | -0.25172 | -0.1607 | **0.899187** | 0.106544 | -0.23971 | 0.010969 |
| Fly sex | 0.318961 | **-0.55652** | -0.18567 | 0.295966 | 0.044378 | 0.102641 | **0.672535** | -0.04092 |
| Latitude | **0.633488** | 0.273895 | 0.014031 | -0.13291 | 0.019847 | -0.08264 | 0.042753 | **0.704812** |
| Longitude | -0.4249 | 0.272585 | -0.35607 | 0.276818 | -0.01672 | **0.602872** | 0.140257 | 0.397913 |
| Altitude | -0.45929 | -0.55462 | 0.140913 | -0.05817 | -0.05272 | -0.32878 | -0.0875 | **0.582814** |
| Julian Day | -0.25476 | 0.290205 | **0.590708** | -0.1775 | 0.359869 | -0.02122 | **0.583053** | 0.022985 |
| Host mass | -0.01742 | -0.18112 | -0.1681 | **-0.85963** | -0.23625 | 0.346234 | 0.150925 | -0.03463 |

Figure S2. PCA Scree plot for the two morphotypes analysis showing the percentage contribution of the eigenvalue of each principal component.


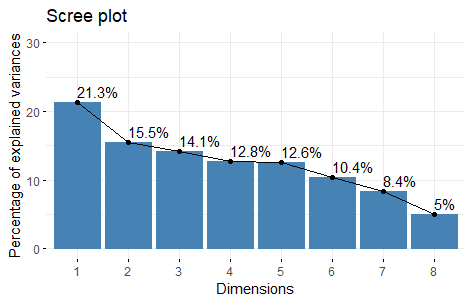


Table S4. Contributions of the variables to the principal components (loadings), with values over 0.5 highlighted in bold typeface, for the analysis of three morphotypes of *Ornithomya fringillina*

|  | **PC1** | **PC2** | **PC3** | **PC4** | **PC5** | **PC6** | **PC7** | **PC8** |
| --- | --- | --- | --- | --- | --- | --- | --- | --- |
| **Fly wing** | -0.18512 | **0.594653** | -0.00992 | 0.204191 | 0.224839 | **0.663969** | -0.28091 | -0.00298 |
| **Scutellar bristles** | 0.048662 | -0.0316 | -0.27951 | **0.836367** | 0.290779 | -0.18391 | 0.316687 | 0.018134 |
| **Latitude** | **0.628791** | 0.145387 | -0.2264 | -0.09226 | 0.043162 | 0.048709 | -0.00835 | **-0.7207** |
| **Longitude** | -0.44598 | 0.303067 | 0.197818 | -0.04102 | 0.322081 | **-0.58989** | -0.23355 | -0.40273 |
| **Altitude** | -0.46519 | -0.45001 | 0.129226 | 0.210677 | -0.29056 | 0.340544 | 0.080832 | **-0.55953** |
| **Julian Day** | -0.1992 | -0.20506 | **-0.79322** | -0.05744 | -0.05949 | -0.06804 | **-0.52542** | 0.03929 |
| **Host Mass** | 0.117181 | 0.329418 | 0.122548 | 0.369097 | **-0.77106** | -0.23099 | -0.2793 | 0.024391 |
| **Fly sex** | 0.315072 | -0.42348 | 0.412883 | 0.254336 | 0.277111 | 0.032032 | **-0.63685** | 0.053343 |

Figure S3. PCA Scree plot for the three morphotypes analysis showing the percentage contribution of the eigenvalue of each principal component.


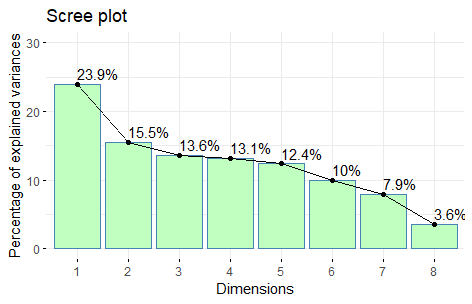

Supplement: Supplementary file 4 — Supplementary Material 4 [file 11686_2025_1113_MOESM4_ESM.docx]
